# Potential therapeutic applications of stem cells in animal models of ocular affections

**DOI:** 10.1186/s41232-025-00380-7

**Published:** 2025-07-21

**Authors:** Taghreed A. Hassan, Yara S. Abouelela, Hamdy Rizk, Ayman Tolba

**Affiliations:** Anatomy and Embryology Department, Faculty of Veterinary Medicine, Cairo University, Giza, 12211 Egypt

**Keywords:** Limbal deficiency, Glaucoma, Retinopathy, Macular degeneration, Stem cells, Animals, Ocular regeneration

## Abstract

**Background:**

Ocular affections are serious damage to the ocular tissue that results in impaired vision or blindness. Cell-based therapies are a potentially effective therapeutic technique that entails using stem-like precursor cells to induce differentiation of specific cell types and implanting the cells to improve vision in the affected tissue area.

**Methods:**

Numerous clinical trials were started to investigate the potential benefits of stem cells for treating ocular affections, based on several encouraging findings from the preclinical research. Following our review, data were collected from various databases, “Google Scholar, Springer, Elsevier, Egyptian Knowledge Bank, ProQuest, and PubMed” using different keywords such as corneal ulcer, retinopathy, glaucoma, ocular regeneration, and stem cells to investigate the various methods for regeneration of ocular affections. The data were obtained and analyzed.

**Results:**

This review includes tables that show all types of stem cells that were used to treat ocular diseases, such as mesenchymal stem cells (MSCs), hematopoietic stem cells, neural stem cells, embryonic stem cells, and induced pluripotent stem cells. The several characteristics of MSCs that aid in the restoration and regeneration of injured ocular tissue are outlined in this paper, along with their potential applications in the management of ocular degenerative diseases, as determined by physical, histological, immunohistochemical, and biochemical evaluations. Finally, our review highlights the most effective regenerative strategies that assist in rapid ocular regeneration in a variety of animal models, including mice, rats, rabbits, and goats.

**Conclusion:**

With the promising results of multiple preclinical studies, stem cell therapy is still a great choice for treating ocular degenerative illnesses. To improve the clinical outcomes, co-transplantation of two or more cell types may be a possibility for future treatment alternatives.

## Introduction

Vision loss is a big issue; over 285 million people worldwide are believed to be visually impaired, and 39 million are blind; additionally, over 7 million more blind people occur annually in the world. Macular degeneration, diabetic retinopathy, diabetic macular oedema, cataracts, uveitis, keratitis, and glaucoma are the leading causes of visual loss [1, 2].

Many cases of blindness are brought on by ocular surface disorders. Allogenic corneal transplantation is frequently used in these situations to restore vision. However, due to the severity of some patients’ ocular surface conditions, this approach may not be appropriate for them; also, rejection frequently occurs, necessitating repeat grafting in order to achieve the best possible visual rehabilitation [3].

Topical medicine administration is the most widely used form and the typical way of giving medication to the eyes. Unfortunately, the relatively low ocular drug bioavailability of this form of administration poses significant challenges to the efficient treatment of several ocular disorders. The prevalent therapies for diseases of the posterior eye face several obstacles, such as the need for regular intraocular injections, potential side effects, and elevated treatment expenses [4]. Therefore, traditional therapies for ocular disorders, such as surgery and ocular drugs, are only able to slow the progression of ocular diseases and are not able to reverse the permanent vision loss associated with degenerative retinal diseases [5, 6].

Novel approaches like cell-based therapeutics and gene therapy are now being developed for ocular diseases to regenerate the damaged corneal and retinal architecture. Cell treatments for corneal diseases are the most often studied anterior segment diseases [7, 8]. Mesenchymal stem cells (MSCs) and limbal stem cells (LSCs) are the sources of corneal treatments. Retinal tissue is the other ocular component for which cell therapies are being studied. Retinal pigmented epithelium (RPE) and retinal cell types can be generated in vivo by using neural stem cells (NSCs), induced pluripotent stem cells (iPSCs), or embryonic-like stem cells (ESCs) [9].

Mesenchymal stem cells (MSCs) can be easily identified and grown from bone marrow, adipose tissue, amniotic fluid, and Wharton’s jelly of the umbilical cord. Due to their immunomodulatory, anti-inflammatory, and anti-angiogenic effects, as well as their capacity to prevent corneal scarring, mesenchymal stem cells have attracted a lot of attention in the field of ocular regeneration [10–12].

In this review, we focused on the various types of stem cells and their potential therapeutic effects. We also highlighted the potential role of MSCs in treating common ocular diseases such as glaucoma, retinal dystrophy, macular degeneration, uveitis, and diabetic retinopathy, as well as corneal ulcers and limbal stem cell deficiency.

## Materials and methods

The databases MEDLINE, Embase, PubMed, Google Scholar, ProQuest, BMC, Elsevier, and Egyptian Knowledge Bank were searched for publications published between 2000 and late 2024. We also screened the references from retrieved papers to identify additional related pre-clinical studies. The search strategy was developed utilizing the PRESS checklist and compared to the PRISMA-S criteria. Databases were searched independently, rather than simultaneously on the same platform.

### Search strategy

We searched the literature using the following terms: (“Stem cell” OR “Stem cells and Eye” OR “Adipose Tissue” OR “Bone Marrow” OR “Embryonic stem cells” OR “Induced pluripotent stem cells” OR “Limbal stem cells”) AND (“Corneal ulcers” OR “Limbal stem cell deficiency” OR “Retinal diseases” OR “Retinal degeneration” OR “Retinitis pigmentosa” OR “Glaucoma” OR “Diabetic retinopathy”). We also used Google search to find current and relevant publications about the role of stem cells and their mechanism in corneal and retinal regeneration. Finally, we looked for ocular obstacles and the characteristics of these barriers.

### Inclusion and exclusion criteria

The inclusion criteria were the following: (1) Animals with ocular diseases, such as corneal and retinal disorders. (2) Animals that have received stem cell treatment. (3) The research was published in English. The following were the exclusion criteria: (1) stem cell in vitro research; (2) case reports, editorials, letters to the editor, clinical studies, and abstracts from conferences; and (3) studies of languages other than English.

### Data extraction

Two researchers independently reviewed titles and abstracts based on the eligibility criteria. All differences were settled through adjudication by a third researcher. Extracted data includes the author’s animal type, age, gender, study design, type of ocular disease, number of treated eyes, follow-up period, diagnosis, stem cells, administration routes, and the most significant finding. For research with similar findings, only the most complete publication was considered.

## Results

Only papers with abstracts were examined. One hundred seventy-nine publications were analyzed after being selected based on their title and abstract. The results were then split and categorized by ocular disorders, which included LSCD (24), corneal ulcer (41), macular degeneration (11), retinitis pigmentosa (9), glaucoma (17), diabetic retinopathy (16), retinal ischemia (8), uveitis (3), retinal detachment (1), optic neuropathy (14), and other retinal diseases (35). Each study was evaluated by at least two reviewers, and ratings were based on the reviewing authors’ consensus. A summary of the most significant studies is reported in Tables 1, 2, 3, 4, 5, 6, 7, 8, and 9.

**Table 1:** Summary of stem cells used in the treatment of limbal stem cell deficiency (LSCD) in different animals

Animal	Type of therapy	Source of stem cells	Type of injury	Site of injection	Clinical evaluation	Clinical results	References
Rabbit	Limbal epithelial stem cells expanded on rabbit amniotic membrane (AM)	Rabbit limbus	Total limbal stem cell deficiency	The membrane sutures through the edge of the membrane to the conjunctiva	Clinical and phenotypic analysis Histology Immune Histo-chemistry	After a 1-year follow-up, the control group exhibited 100% failure, and the limbal stem cell-treated group showed 26% success (*p* = 0.001). Clinical failure connected with the J phenotype (*p* = 0.001), whereas clinical success correlated with the K phenotype (*p* = 0.01)	[13]
Rabbit	Cryopreserved corneal limbal stem cells	Rabbit limbal lamellar	Limbal stem cell deficiency	The membrane is fixed with a suture on the sclera	Clinical observation Histological Examination Electron microscope Immunohistochemical examination	The corneal transparency of the experimental rabbits was significantly improved Cryopreserved corneal LSCs can repair damaged rabbit corneas	[14]
Rabbit	Corneal limbal epithelial cells in a Thermoreversible Polymer	Rabbit	Limbal stem cell deficiency	Cultured LSCs were placed on the surface of the cornea	Clinical evaluation Immunocytochemistry quantitative RT-PCR	The corneal epithelium had grown successfully in each of the seven eyes with a positive histology The limbus of the seven successful eyes displayed the corneal phenotype and stem cell-associated markers by immunohistochemistry and reverse transcriptase polymerase chain reaction, demonstrating the homing of these cells into the limbus	[15]
Rabbit	Rabbit limbal epithelial cell sheets were cultivated with human bone marrow–stem cells	Human bone marrow	Total limbal stem cell deficient by 1-n-heptanol and mechanical debridement of the corneal epithelium	Cell sheets were transplanted to the ocular surface	Slit lamp microscopy Histology Immunohistochemistry Real-time qPCR	N-cadherin, hepatocyte growth factor, and keratinocyte growth factor were all expressed by bone marrow stem cells In rabbits lacking limbal tissue, transplanted epithelial sheets restored the corneal phenotype	[16]
Rabbit	Human immature dental pulp stem cells (hIDPSCs) Cultured on amniotic membrane carriers	Human	Total limbal stem cell deficiency	The sheet of hIDPSCs was placed directly onto the exposed transparent stromal bed	Histological Immunohistochemical evaluation Transmission electron microscopy Real-time RT-PCR analysis	hIDPSCs are comparable to LSC in certain aspects and could be a viable substitute cell source for corneal restoration Similar to LSC, hIDPSCs express markers like ABCG2, integrin β1, vimentin, p63, connexin 43, and cytokeratins 3	[17]
Rabbit	Human immature dental pulp stem cells (hIDPSCs) Cultured on amniotic membrane carriers	Human	Total limbal stem cell deficiency	hIDPSC sheet was transplanted onto the corneal bed	Histological Immunohistochemical evaluation Transmission electron microscopy	During the follow-up period, the corneal transparency of the rabbit eyes that received hIDPSC transplantation improved, but the corneas in the control group experienced complete conjunctivalization and opacification	[18]
Rabbit	BMSCs	Rabbit bone marrow aspiration	Limbal stem cell deficiency	MSCs were injected under the amniotic membrane on the injured cornea	Clinical evaluation Histology Immunohistochemistry	All eyes displayed complete conjunctivalization of the corneal surface four to five weeks following the injury Following MSC transplantation, the expression of integrin and connexin 43 may show whether the cells are able to retain their stem cell characteristics or undergo trans differentiation into epithelial progenitor cells	[19]
Rabbit	Mucin-expressing cord-lining epithelial cell (CLEC-muc) expanded on human amniotic membrane	Human umbilical cord	Limbal stem cell deficiency	CLEC-muc sheet was transplanted onto the denuded cornea stromal surface	Histological analysis Immunohistochemistry Reverse transcription-polymerase Chain Reaction	When CLEC-muc sheet was transplanted into rabbit eyes lacking limbal stem cells, the corneal surface recovered and became smooth and clear The normal corneal-specific epithelial markers CK3 and CK12 were phenotypically expressed	[20]
Rabbit	Scaffold-free embryonic stem cell sheets	Mice embryo	Limbal stem cell deficiency	Scaffold-free embryonic stem cell sheets were expanded on the bare corneal stroma	Histology Immunocytochemistry Single-cell atomic force microscope measurement	Reconstruction of the ocular surface in 75% of the treated rabbits Embryonic stem cells differentiated into corneal epithelial cells when in direct contact with the stroma	[21]
Rabbit	Oral mucosal epithelial stem cells sheet	Rabbit interior buccal mucosa	Limbal stem cell deficiency	The membrane was placed directly onto the exposed stromal bed	Histological Immunohistochemical evaluation Real-time RT-PCR analysis	The ocular surfaces were clear and smooth and consisted of only oral mucosal epithelial stem cells or heterogeneously mixed with corneal epithelial cells	[22]
Rabbit	Skin epithelial stem cells transduced with paired box protein (PAX6)	Rabbit skin	Limbal stem cell deficiency	Epithelial stem cells transplanted into the corneas	Histopathology Immunohistochemistry	Skin epithelial stem cells can be reprogrammed to become LSC-like cells by transduction of PAX6, and these reprogrammed cells can heal injured corneal surfaces and replace CECs when transplanted onto eyes in a rabbit corneal injury model	[23]
Rabbit	Human iPS cell-derived corneal epithelial cell (human iCEC) sheets	human	Total limbal stem cell deficiency	Harvested human iCEC sheets were grafted onto rabbit corneas	Histology Scanning electron microscopy Immunofluorescence Real-time RT-qPCR Microarray analysis	The sheets exhibited the classic corneal differentiation markers CX43, K3, and K12, as well as the corneal limbal stem-cell markers K15 and K19 Through sorting and ex vivo expansion, cells isolated from the ocular surface ectodermal zone can produce a corneal epithelium and restore functions in a rabbit model of corneal blindness	[24]
Rabbit	Limbal epithelial stem cells cultured on poly (ethylene glycol)-modified silk fibroin membrane	Rabbit limbus	Limbal stem cell deficiency	The membrane transplanted on the corneas	Histology immunofluorescence Scanning electron microscopy Optical coherence tomography	The membrane inhibited new blood vessels and rescued corneal epithelial defects In addition, increased corneal epithelial thickness and stromal thickness	[25]
Rabbit	Limbal stem cells on decellularized human amniotic membrane	Rabbit limbus	Limbal stem cell deficiency	LSC-seeded decellularized human amniotic membrane transplanted into the damaged eye	Histology Immunohistochemistry Real-time polymerase chain reaction analysis	The composite membrane enhanced LSC survival, retention, and organization; it decreased inflammation and neovascularization; it enhanced the re-epithelialization of the defect area; and it preserved the pro-regenerative and immunomodulatory qualities of the decellularized amniotic membrane	[26]
Rabbit	Limbal stem cells implanted on a type I collagen membrane	Rabbit limbus	Limbal stem cell deficiency	The construct was placed over the exposed surface of the removed limbal zone	Clinical evaluation Histology Immunohistochemistry Optical coherence tomography	The experimental group of rabbits showed transparency and corneal epithelium regeneration free of epithelial abnormalities. In addition, there were no goblet cells in the corneal epithelium’s core zone	[27]
Rat	BMSCs seeded on the amniotic membrane	Human	Limbal stem cell deficiency	The membrane was sutured onto the corneal surface using 8/0 Vicryl sutures	Slit lamp evaluation Immunocytochemical analysis optical coherence tomography analysis Quantitative real-time polymerase chain reaction	Serial slit lamp evaluation revealed remarkable improvement in corneal regeneration Whereas histologic and optical coherence tomography analyses demonstrated corneal histoarchitecture and thickness resembling that of a normal cornea	[28]
Rat	Oral mucosal epithelial cells implanted on porous silicon membranes	Rat	Limbal stem cell deficiency	Scaffolds bearing cells were implanted close to the limbus	Histology immunohistochemistry Multiplex-nested PCR	Scaffolds supported transplanted rat oral mucosal epithelial cells in vitro and in vivo and recapitulated some aspects of an artificial stem cell niche	[29]
Rat	ADSCs transfected with the PAX6 gene	Rats inguinal region	Limbal stem cell deficiency	PAX6-transfected ADSCs were placed into an application tube adhered to the damaged cornea for 1 h	Histological Examination Immunohistochemical staining Western blot analysis	PAX6-transfected ADSCs attached to and replenished the damaged cornea through the formation of corneal epithelium	[30]
Rat	Human ADMSCs with LSC-specific medium	Human	Limbal stem cell deficiency	Amniotic membrane implant with ADMSCs cultured in LSC medium	Clinical evaluation Histopathology Quantitative real-time PCR (qRT-PCR)	Human ADMSCs in an LSC-specific media can reduce inflammation and neovascularization while promoting ocular wound repair	[31]
Mice	BMSCs, limbal stem cells growing on polyamide nanofibers	Mice femur, limbus	Limbal stem cell deficiency	The nanofibers with growing cells were transferred to the cornea	Immunofluorescence Real-time PCR	Transfer of LSCs and MSCs to reduce a local inflammatory response Two days following the procedure (during which the corneal epithelium is removed), and 7 and 14 days following the cell transfer (during which the corneal epithelium is restored)	[32]
Mice	Hair follicle bulge-derived stem cells (HFSCs) transplanted on a fibrin carrier	Mice hair	Limbal stem cell deficiency	HFSCs on the fibrin carrier were sutured onto the eye	Assessment of barrier function histology immunofluorescence	In 80% of transplanted rats, the HFSC transplant was able to repair the ocular surface by developing into corneal epithelial-like cells, expressing Krt12, and repopulating the corneal SC pool while inhibiting conjunctival ingrowth and vascularization	[33]
Goat	Epidermal adult stem cells (EpiASC)	Goat ear skin	Limbal stem cell deficiency	The amniotic membrane, on which autologous epidermal stem cells are fixed to the sclera surface with interrupted sutures	Immunohistochemistry Transmission optical microscopy	In goats with entire LSCD, EpiASC healed the cornea and rebuilt the skin, with hair showing in the restored areas These findings support the ability of EpiASC to differentiate into various functional cell types both in vivo and in vitro	[34]
Goat	Epidermal adult stem cells (EpiASC) were cultivated on the human amniotic membrane	Goat ear skin	Total limbal stem cell deficiency	The sheets were surgically transplanted into the cornea	Clinical observation Histology immunohistochemistry	Goats receiving EpiASC treatment, which improved their postoperative visual acuity and restored corneal clarity The role of the reconstructed corneal epithelium (RCE) was to secrete material that resembled glycocalyx It also expressed the proteins CK3, CK12, and PAX-6	[35]
Goat	Cryopreserved limbal stem cells on human amniotic membrane	Goat	Limbal stem cell deficiency	The membrane was transplanted into the experimental goats by surgery	Clinical observation Histological analysis Polymerase chain reaction analysis	Transplantation of cryopreserved LSCs that resemble fresh LSCs reconstructs the damaged goat corneal surface gradually	[36]

**Table 2:** Summary of stem cells used in the treatment of corneal ulcers and burns in different animals

Animal	Type of therapy	Source of stem cells	Type of injury	Site of injection	Clinical evaluation	Clinical results	References
Rabbit	BMSCs	Rabbit bone marrow	Corneal alkali burn	The injections were given through the ear vein	Clinical evaluation immunohistochemistry bone marrow function check	Following alkali burn, well-formed neovascularization emerged on day 14 At various times, there was increased expression of PCNA, P63, and vimentin MSCs expressed a-SMA, which led to their differentiation into myofibroblasts	[37]
Rabbit	Rabbit limbal and central corneal epithelial stem cells	Rabbit	Corneal alkali burn	The central corneal incision causes the limbal cells to rise in population size temporarily	Histological immunohistochemical evaluation Real-time qPCR	By using both in vivo transplantation and 3D culture, limbal stem cells were able to restore the cornea The undifferentiated, epithelial cell population seen in the rabbit limbus is a side population whose size temporarily increases in response to central corneal injury	[38]
Rabbit	Bone marrow mesenchymal stem cells suspended in fibrin gels	Rabbit femurs	Alkali burn wounds	Fibrin gels were transplanted onto the rabbit cornea	Slit lamp microscope Histological analysis Immunofluorescence	Upon the transplantation of BMSCs, the rabbit's wounded corneal surface was successfully restored, and some BMSCs expressed CK3 and helped the corneal epithelium repair	[39]
Rabbit	Human ASCs on an HA-derived scaffold	Human	Corneal ulcer, a flap of 120 µm in depth, was created on the cornea using a microkeratome blade	The scaffold is inserted into the corneal stroma of the rabbits	Slit lamp examination histological examination immunohistochemical examination	The stem cells expressed human cornea-specific proteins, as shown by immunostaining of keratocan, aldehyde dehydrogenase, and type I collagen Keratocytes from human ASCs on HA-derived scaffolds can be used to regenerate extracellular matrix	[40]
Rabbit	Conditioned media from human amniotic epithelial cells	Human amniotic tissue	Chemical burn of the cornea	Cells were injected into the dorsal bulbar subconjunctiva	Evaluation of corneal wound healing using fluorescein staining Corneal histological examination	Corneas had less inflammatory cell infiltration and showed more intact epithelial features than the other groups	[41]
Rabbit	Human adipose-derived mesenchymal stem cells	Human	Corneal alkali burn	Subconjunctival	Corneal haziness grading Histopathology Quantitative real-time PCR (qPCR)	Compared to the control group, the experimental group demonstrated quicker wound healing, and the outcome was a clearer corneal medium Histologically, the experimental group's corneas had five to six epithelial cell layers, while the control group’s corneas had two to three cell layers	[42]
Rabbit	Human adipose-derived stem cells overlaid on a scleral contact lens carrier	Human	Ocular alkaline burn	hASCs cultured on SCLs were placed on the damaged eye	Clinical observation Light and electron microscopic examination	Human adipose-derived stem cells were readily confluent and adhered to the SCL surface Compared to SCL eyes, human adipose-derived stem cells on SCL eyes demonstrated less corneal neovascularization, less corneal opacity, and a smaller epithelial defect	[43]
Rabbit	Human ADSCs are seeded on decellularized corneal sheets	Liposuction	The corneal stromal pocket of 50% depth	The graft is implanted into the Corneal stromal pocket	Clinical observation Histological examination Immunohistochemistry	Survival of the transplanted human stem cells inside the graft and their differentiation into functional keratocytes	[44]
Rabbit	Human amniotic membrane-derived mesenchymal stem cells hAM-dMSCs	Human amniotic membrane	Corneal Alkali Burn	Subconjunctival	Clinical evaluation Histology Immunocytochemistry Enzyme-linked immunoassay (ELISA)	The treated groups showed reduced levels of corneal opacification and neovascularization as well as quicker corneal epithelial repair as compared to the control group These findings showed that in corneal alkali wounds, hAM-dMSCs might improve epithelial healing while lowering corneal opacification and neovascularization	[45]
Rabbit	Bone marrow mesenchymal stem cells	Rabbit tibia and femur	Corneal alkali burn	Intravenous	Histological examination Immunohistochemistry	The MSC-treated group displayed the best histology outcomes at 28 days, with nearly repaired corneas. In contrast, BMSC-treated group demonstrated a higher expression of vimentin when evaluating the capacity of BM-MSCs to differentiate	[46]
Rabbit	ADSCs	Inguinal fat	Alkali injured cornea	Intrastromal, subconjunctival injections, and topical application	Clinical observation Histological examination Immunohistochemistry	MSCs resulted in almost normal architecture of eye tissues Reduction of a-SMA in the MSC group with higher mitotic-regenerative activity with the presence of Ki67	[47]
Rabbit	Human AD-MSCs or rabbit AD-MSCs	Human liposuction, retroperitoneal from rabbits	The center of the donor and recipient cornea was excised with trephine	Intrastromal, intravenous injections of MSCs	Histopathology Immunohistochemistry Leukogram	Administering AD-MSCs locally or systemically to prevent corneal rejection may not improve survival; on the contrary, it may worsen inflammation and neovascularization and undermine the innate immunity of the eye	[48]
Rabbit	Human ADMSCs colonized polyethyl acrylate	Human adipose tissue	A 7-mm diameter intrastromal pocket was created in the central cornea using a blade	The membrane was placed and centered inside the cornea	Clinical observation Histological analysis Scanning electron microscope	There was a little decrease in the h-ADMSCs colonized materials’ extrusion rate Regarding transparency and neovascularization, no discernible differences were found between the groups with and without h-ADMSCs	[49]
Rabbit	Limbal epithelial stem cells (LSCs), as well as bone marrow (BM-MSCs) or adipose tissue (ADMSCs) cultured on nanofiber scaffolds	Femur rabbit Subcutaneous adipose tissue	Corneal Alkali burn	Stem cell-seeded nanofiber scaffolds were positioned and stitched to the conjunctiva	Clinical evaluation Histology Immunocytochemistry Quantitative real-time PCR (qPCR)	The eyes treated with BM-MSCs and LSCs showed comparable clinical healing characteristics, evaluation of corneal thickness, re-epithelialization, neovascularization, and suppression of a local inflammatory reaction; however, the outcomes were significantly better than those of untreated eyes or eyes treated with a nanofiber scaffold alone or with a nanofiber scaffold seeded with ADMSCs	[50]
Rabbit	Human amniotic membrane-derived mesenchymal stem cells (hAM-dMSCs)	Human amniotic membrane	Corneal Alkali burn	Intracameral injection	Clinical evaluation Histological examination Immunofluorescence	Neovascularisation, opacity, stromal inflammatory cell infiltration, and stromal α-SMA + cells are all decreased by intracameral hAM-MSC injection These findings imply that intracameral hAM-MSC injection causes an environment that is anti-inflammatory and anti-fibrotic, hence facilitating the healing of corneal wounds	[51]
Rabbit	Human umbilical cord mesenchymal stem cells	Human umbilical cord	Bullous keratopathy	The cells placed on the stromal bed	Immunocytochemistry Western blot analysis Quantitative RT-PCR	Corneal thickness and transparency were successfully preserved by transplanting umbilical cord cells into a rabbit model of bullous keratopathy For the treatment of corneal endothelial disease, tissue-engineered corneal endothelium from the umbilical cord may serve as a source of allogeneic cells	[52]
Rabbit	LSCs encapsulated in alginate-chitosan hydrogel	Rabbit limbus	Alkali burn wounds	Alginate-chitosan hydrogel cross-linked in the corneal wound	Visual observation Slit lamp Examination Histological analysis Immunofluorescence	The hydrogel encapsulating LSCs improves epithelial reconstruction and may serve as a rapid and effective method for corneal wound healing	[53]
Rabbit	ADSCs	Inguinal fat	Corneal alkali burn	Intrastromal, subconjunctival injections, and topical application	Clinical observation Histological Immunohistochemical evaluation	Improve the corneal sensation Restoration of normal corneal architecture in the group treated with AMSCs	[54]
Rabbit	BMSCs on a temperature-responsive membrane	Tibia and femur	Alkaline injured cornea	The membrane was placed over the damaged cornea for 30 min to allow the adherence of cells to the corneal surface	Clinical evaluation Histopathology Immunohistochemistry Real- time qPCR	Corneal transparency improved 1 week after MSCs transplantation, while Complete re-epithelialization of the injured cornea was observed 2 weeks after MSCs transplantation	[12]
Rabbit	Fetal cartilage-derived stem cells (FCSC)	Femoral head of immature cartilage tissue	Chemically burned rabbit model	FCSC-sheet was placed on the cornea and sutured	Immunocytochemistry Western blots	FCSC created a cell sheet that successfully differentiated into corneal epithelial cells The implanted cell sheet maintained its transparency, and the cells were alive a week after implantation	[55]
Rabbit	Human umbilical cord mesenchymal stem cells carried on the 3D scaffold	Human	Corneal ulcer, a diameter of 9-mm pocket was created in the corneal stroma with	MSCs and the 3D scaffold were transplanted into the pocket	Immunohistochemistry Immunofluorescence Alcian blue staining Scanning electron microscope	Implanting the scaffold into the corneal stroma showed no significant immune rejection, which indicated that the scaffold and corneal tissue were well compatible By introducing scaffolds into the rabbit corneal stroma with differentiated chondrocytes, the corneal thickness was enhanced, allowing the chondrocytes to remain stable within the cornea	[56]
Rat	BMSCs expanded on the human amniotic membrane	Human bone marrow	Chemical alkali burn	Amniotic membrane with grown cells was sutured into the corneal surface	Slit lamp microscope Histology Immunohistochemistry Immunofluorescence	Similar to limbal epithelial stem cells, MSC transplantation was successful in reconstructing the injured rat’s ocular surface It's interesting to note that, rather than MSCs differentiating into epithelial cells, the therapeutic benefit of transplantation may be linked to the suppression of inflammation and angiogenesis following MSC transplantation	[57]
Rat	Rat mesenchymal stem cells	Rat	chemically burned corneas	MSC applied topically to the damaged cornea	Histopathology Quantitative real-time PCR (qRT-PCR) Enzyme-linked immunosorbent assay (ELISA)	MSC inhibited corneal neovascularization and inflammation, downregulated IL-2, but elevated IL-10, TGF-B1, and IL-6. They also lowered CD4 + cell infiltration and upregulated TSP-1 expression while downregulating MMP-2 expression	[58]
Rat	BMSCs induced by rat corneal stromal cells (CSCs)	Rat	Chemical alkali burn	MSCs induced by CSCs were transplanted onto the cornea	Slit lamp observation Histology Immunohistochemistry Confocal laser corneal microscopy	The group that received induced MSCs showed a significant decrease in corneal opacity, fluorescence staining, and neovascularization grades Following co-cultivation with CSCs, the generated MSCs had CK12-positive staining, a feature of corneal epithelial cells that was verified by SEM	[59]
Rat	Bone marrow mesenchymal stem cells	Rat tibia and femur	Corneal alkali burn	Subconjunctival	Clinical evaluation Histological examination Quantitative real-time PCR (qPCR) Enzyme-linked immunosorbent assay (ELISA)	MSCs markedly improved corneal epithelial regeneration and reduced the region affected by corneal neovascularization (CNV) Subconjunctival injection of MSCs greatly lowers CNV in alkaline-burned corneas, attenuates inflammation, and speeds up corneal wound healing; these effects were linked to downregulated TNF-a, VEGF, and MIP-1a and a decrease in infiltrating CD68 + cells	[60]
Rat	h-ADMSCs	Human subcutaneous adipose tissue	Chemical alkali burn	Topical drops	Clinical evaluation Histological Examination Immunofluorescence	Comparing the stem cell-treated corneas to the control eyes, histology revealed that the former had full re-epithelization, fewer inflammatory cells, and a smaller fibroblast activation structure	[61]
Rat	Polysaccharide hydrogel combined with BMSCs	Bone marrow of rat long bones	Chemical alkali burn	Polysaccharide hydrogel was applied in conjunction with subconjunctival injection of MSCs	Histological examination quantitative real-time PCR (qRT-PCR) Enzyme-linked immunosorbent assay (ELISA)	The combination resulted in better recovery of corneal epithelium and reduction in inflammation, neovascularization, and opacity of the healed cornea	[62]
Rat	Bone marrow and adipose-derived mesenchymal stem cells	Rabbit tibia and femur, pubic adipose tissue	Chemical alkali burn	Subconjunctival	Clinical evaluation Histological Examination Immunofluorescence immunohistochemistry	MSCs produced from bone marrow and adipose tissue both significantly reduce tissue inflammation and promote corneal damage healing When comparing the groups receiving bone marrow and adipose-derived MSC to the alkaline burn group, the IL-1b and TNF-a staining scores as well as the quantity of CD68- and Cas-3-positive stained cells were considerably lower	[63]
Rat	BM-MSCs	Rats	Ultraviolet-induced corneal injury	Intravenous versus subconjunctival injection	Histopathology Immunohistochemistry Electron microscopy assessment	Minimal changes were observed in rats treated with BM-MSCs with more improvement associated with the subconjunctival administration compared to the Intravenous route Local injection of BM-MSCs has an amazing regenerative efficacy on the corneal injury compared to the systemic Intravenous route	[64]
Rat	Human umbilical mesenchymal stem cells	Human	Irradiation-induced photo keratitis	Subconjunctival injection	Histology Immunohistochemistry Intraocular pressure measurement	The degree of damage to the surrounding corneal tissue is lessened by MSCs MSCs enhance the disarray of collagen and fibronectin in the corneal stroma, speed corneal epithelial regeneration, and lessen inflammation and neovascularization	[65]
Mice	Embryonic stem cells (ESCs) cultivated on type IV collagen	Mouse	Cornea treated with n-heptanol	The graft cells were put into the tube and adhered to the damaged cornea	Histological analysis Reverse transcription–polymerase chain reaction Western blot analysis	Keratin (K)12, unique to corneal epithelial cells, and cell surface CD44 and E-cadherin, both critical for corneal epithelial wound repair, were expressed by these progenitor stem cells Within twenty-four hours following transplantation, the corneal surface had fully reepithelialised During the follow-up period, the corneal epithelial cells showed markers of the transplanted cells	[66]
Mice	Pax6-transfected embryonic stem cells	Mice	Cornea treated with n-heptanol	The graft cells were put into the tube and adhered to the damaged cornea	Histological analysis Immunofluorescence Reverse transcription–polymerase chain reaction	E-cadherin, CD44, and cytokeratin12—a particular keratin of corneal epithelial cells—were expressed by pax6-transfected cells They combined to form a colony that had a reticular structure staining pattern for CD44, E-cadherin, and cytokeratin 12 The cells were maintained alive on the cornea when they were transplanted into injured corneas	[67]
Mice	Embryonic stem cells (ESCs) cultivated on type IV collagen	Monkey	Cornea treated with n-heptanol for induction of corneal injury	The graft cells were transplanted into the injured cornea	Immunostaining Reverse transcription–polymerase chain reaction Confocal Laser microscopic analysis	Multiple cell layers formed due to the transplanted corneal epithelium-like cells adhering to the corneal stroma The cells grown on type IV collagen resembled cobblestones and were similar to epithelial cells	[68]
Mice	Orbital fat-derived stem cells (OFSCs)	Human orbital fat tissue	Chemical alkali burn	Topical administration or intralimbal injections in the cornea	Histology Immunocytochemistry Immunofluorescence Western blot analysis	Topical OFSCs facilitated corneal wound healing by re-epithelializing the cornea, decreased stromal infiltration and corneal edema caused by alkali within the first three days The use of OFSCs topically outperformed the injection of IL. A prolonged corneal haze was linked to the OFSCs from the intralimbal injection clustering in the limbal area and central corneal epithelium	[69]
Mice	Limbal biopsy-derived stromal cells	Human limbus	Corneal debridement wounds	Fibrin gel with limbal stem cells was injected into the wound	Optical coherence tomography Transmission electron microscopy Immunofluorescences	LSCs prevent the formation of scar tissue and induce the regeneration of eroded stroma	[70]
Mice	BMSCs	Mice femur	Mechanical removal of corneal epithelium and anterior stroma	BMSCs were injected into the tail vein	Immunocytochemical analysis Real-time polymerase chain reaction ELISA	MSCs can restore corneal transparency by secreting high levels of hepatocyte growth factor (HGF) HGF alone can restore corneal transparency	[71]
Mice	Corneal epithelial cells differentiated from human embryonic stem cells	Human	Decellularization of the cornea using NaoH and ultraviolet light	The cells injected into the anterior chamber using a microliter syringe	Histopathology Immunohistochemistry Real-time polymerase chain reaction	In the decellularized murine cornea, the highly proliferative corneal epithelial cells differentiated from human embryonic stem cells could create multilayer epithelium, maintain transparency, and generate intact tight connections on its surface	[72]
Mice	Bone marrow mesenchymal stem cells on amniotic membrane	Mice femur	Corneal injury using Alger brush	Topical, subconjunctival, intraperitoneal, and intravenous	Slit lamp examination Histology Immunofluorescence Quantitative real-time PCR (qPCR)	Subconjunctival, or IV administration, causes higher frequencies of MSCs in ocular surface tissues after corneal damage. Subconjunctival or intravenous therapy decreases tissue fibrosis, inflammation, and corneal opacity	[73]
Mice	Human adipose-derived mesenchymal stromal cells	Human	Ethanol-induced injury in the mouse cornea	The cells injected into the retro-orbital area	Fluorescein cornea angiography Histological analysis Immunofluorescence Quantitative real-time PCR	Treatment with ADSCs lessens corneal fibrosis, decreases corneal thickening during granulation, and lessens neovascularization in damaged corneas Neovascularization is decreased when peripheral neutrophils are depleted during granulation	[11]
Mice	Cryopreserved human bone marrow-derived MSCs	Human	A 2-mm area of the central epithelium was removed by an Alger Brush	Subconjunctival injection	Slit lamp examination Histopathology	Mice injected with MSCs showed no inflammation or scar formation at the site of injection, and no sign of corneal haziness, scarring, or neovascularization	[74]
Mice	Human placental-MSCs	Human placenta	Chemical burn	Subconjunctival injection	Histological analysis Quantitative real-time polymerase chain reaction Western blot analysis	Subconjunctival injection of MSCs exerted anti-inflammatory and anti-apoptotic effects in the cornea reduced inflammatory cytokines diffusion from the damaged cornea after the MSC injection	[75]

**Table 3:** Summary of stem cells used in the treatment of retinal degeneration (macular) in different animals

Animal	Type of therapy	Source of stem cells	Type of injury	Site of injection	Clinical Evaluation	Clinical results	References
Rabbit	Human embryonic stem cell-derived retinal pigmented epithelium	Human	Sodium Iodate–induced retinal degeneration	Subretinal injection	RT-PCR analysis Western blot analysis	Retinal transplanted cells proliferated and moved into the layers of the retina, and produced a minor but noticeable B-wave recovery. Photoreceptor markers S-Opsin and Rhodopsin were expressed by the transplanted cells. Our findings show that associated genes and proteins are expressed by putative hESC-derived retinal cells	[76]
Rat	Bone marrow mesenchymal stem cells (MSCs)	Rat femurs and tibias	Sodium iodate (SI)-induced retinal degeneration	Subretinal transplantation	Histopathology Immunohistochemistry Immunofluorescence analysis	Five weeks following transplantation, viable MSCs were seen, mostly in the subretinal area. Pan-cytokeratin, glial fibrillary acidic protein, and rhodopsin were expressed by the cells Bone marrow MSCs can develop into RPE, photoreceptor, and glial lineage cells when they are delivered into the subretinal region of rats given sodium iodate injections	[77]
Rat	Retinal pigment epithelial cell-derived from human embryonic stem cells	Human	Sodium iodate (SI)-induced retinal degeneration	Subretinal transplantation	Histology Immunohistochemistry Real-time RT-qPCR	The hESC-generated prospective RPE cells showed morphological characteristics, molecular markers, and were associated with the fate of RPEs Grafted RPE cells were seen to be viable in the subretinal area up to four weeks following transplantation, and the expression of RPE markers was confirmed by immunohistochemistry	[78]
Rat	Erythropoietin gene-modified rat MSCs	Rat bone marrow	Sodium iodate (SI)-induced retinal degeneration	Subretinal transplantation	Histology Immunohistochemistry Fundus fluorescein angiography electroretinogram	After transplantation, labeled donor cells took on the shape of the RPE and were seen in the subretinal region Parallel to the improvement in retinal shape and function, the EPO concentration in the vitreous and retina of SI-treated rats transplanted with EPO-rMSCs or Tet-on EPO-rMSCs increased significantly	[79]
Rat	Neural progenitor cell (NPC)-derived from induced pluripotent stem cells (iNPCs)	Human	Royal College of Surgeons rat, a model of age-related macular degeneration	Subretinal transplantation	Histological analysis Transmission electron microscopy immunofluorescence RT-PCR Analysis Western blot analysis	Comparing the number of photoreceptor nuclei in the iNPC-treated eyes to the controls, there were six to eight rows in the former instead of just one The best iNPC-protected regions of the retina were 140 times more responsive to light stimulation than corresponding regions in the contralateral eye, according to electrophysiological recordings	[80]
Mice	Endothelial precursor cell-enriched bone marrow-derived stem cells	Mice	Laser and scar models were created using either a diode laser (150 mW, 1 s, 50 mm) or mechanically by puncturing the retina with a 27-G needle	Intravitreal injection	Immunohistochemistry confocal analysis In vivo retinal angiogenesis quantification assay	Particular interactions occur between BM cells and astrocytes in the retina during both pathological vascular degeneration and normal angiogenesis EPCs found in HSCs can target reactive astrocytes and integrate into an existing template to induce angiogenesis without affecting the retinal structure	[81]
Mice	Bone marrow mesenchymal stem cells (MSCs) neurotrophin-4 (NT-4)	Mice femurs, tibias	Acute Retinal injury using a low dose of sodium iodate	Intravitreal injection	Immunofluorescence analysis Optical coherence tomography (OCT) Electroretinography (ERG) Gene expression analysis	Damaged retinal cells were considerably protected by MSC-NT-4, which actively synthesized NT-4 in the wounded retina The ongoing supply of NT-4, led to notable enhancements in the functioning characteristics of the organs served by ERG	[82]
Mice	Bone marrow mesenchymal stem cells	Mice bone marrow	Laser-induced retinal injury	Intravenous transplantation	Histopathological analysis Quantitative RT-PCR Apoptosis analysis	Analysis showed that following MSC transplantation, the damaged eyes had reduced levels of glial fibrillary acidic protein and matrix metalloproteinase-2 mRNA expression Our findings imply that MSCs administered intravenously can prevent retinal cell death, lessen inflammation, and stop damage from spreading in mice’s retinal injuries caused by lasers	[83]
Mice, monkey	Human embryonic stem cell-derived retinal tissue	Human	Laser-induced model and cobalt-induced retinal degeneration	Subretinal transplant	Histology Electron microscopy Immunohistochemistry Electrophysiology	After transplantation, rod and cone photoreceptors with organized outer nuclear layers were among the retinal cell types that the grafted hESC-retina was seen to differentiate into Further immunohistochemical investigations revealed the establishment of synaptic connections between the host and the graft	[84]
Mice	Adipose-derived mesenchymal stem cells	Human adipose tissue	Sodium iodate (SI)-induced retinal degeneration	ASCs were transplanted in the subretinal space	Immunocytochemical Analysis Enzyme-linked immunosorbent assay Quantitative RT-PCR	Mice given ASCs demonstrated enhanced staining of the RPE and photoreceptor layer and preservation of nuclear layers in the outer nuclear layer ASCs can target injured RPE cells and offer defence against oxidative stress-induced deterioration of the RPE layers	[85]
Mice	Human adipose mesenchymal stem cells combined fetal retinal pigmented epithelium cells	Human	Sodium iodate (SI)-induced retinal degeneration	Subretinal transplantation	Histology immunofluorescence Electroretinography Optical coherence tomography Quantitative RT-PCR	The outer and inner nuclear layers of the retina as well as the total thickness of the retina increased with MSC and RPE cell transplantation Additionally, combined transplantation decreased the expression of caspase 3 and increased the expression of rhodopsin	[86]

**Table 4:** Summary of stem cells used in the treatment of Retinitis Pigmentosa

Animal	Type of therapy	Source of stem cells	Type of injury	Site of injection	Clinical Evaluation	Clinical results	References
Rat	Bone marrow-derived mesenchymal stem cells	Rat femurs and tibias	Royal College of Surgeon’s (RCS) rat as a model of retinitis pigmentosa	Intravenous injection through the tail vein	Spatial visual acuity Luminance threshold Immunohistochemistry Real-time RT-qPCR	While only a single layer of photoreceptors remained in the control animal, both rod and cone photoreceptors (5–6 cells thick) were retained at that time In the eyes of the rats that received MSCs, RT-PCR analysis showed an increase in growth factors, while immunohistochemistry showed an increase in neurotrophic factors	[87]
Rat	BMSCs	Rat femurs and tibias	Royal College of Surgeon’s rat as a model of retinitis pigmentosa	Subretinal transplantation	Immunofluorescence Enzyme-linked immunosorbent assay (ELISA) Real-time RT-qPCR Electroretinography Western blot analysis	After receiving BMSCs, there was a considerable increase in the number of Müller cells expressing proliferative, stem/progenitor, and neuronal markers In the retinas of rats receiving BMSC transplants, Müller cells expressing significant amounts of the nerve growth factor receptor neurotrophic tyrosine kinase receptor type 1 were seen	[88]
Rat	Human bone marrow-derived mesenchymal stem cells combined with retinal progenitor cells	Human	Royal College of Surgeons rats as a model of retinitis pigmentosa	Subretinal transplantation	Immunofluorescence Electroretinogram Real-time RT-qPCR Western blot analysis	Electroretinogram results are maintained far better by combination transplantation than by single transplantation. In addition, the ratio of transplanted cells' photoreceptor development in the retina of RCS rats undergoing combination transplantation was higher than that of single transplantation	[89]
Mice	Bone marrow-mesenchymal stem cells	Mice femur	Rhodopsin knockout mouse as a model of Retinitis pigmentosa	Subretinal transplantation	Immunohistochemical staining Light and electron microscopy Stereo-microscope	According to a histological investigation, cells integrated into layers of the neuroretina that had neuronal and glial morphologies upon transplantation, in addition to the retinal pigment epithelium More importantly, there were notable rescue effects, as evidenced by the presence of intact photoreceptor cells	[90]
Mice	Retinal stem cells (RSCs)	Newborn mice retina	rd1 mice or VPP transgenic mice as a model of Retinitis Pigmentosa	Intravitreal or subretinal transplantation	Immunohistochemistry TUNEL assay for apoptosis	Subjected cells had extensive migration into the ganglion cell layer in VPP animals following subretinal injection One week and 4 weeks’ post-injection, the transplanted cells were found to have neuronal and glial markers expressed locally, including glial fibrillary acidic protein	[91]
Mice	Embryonic stem (ES) cell-derived from retinal pigment epithelial (RPE) cells	Mouse embryo	Retinitis pigmentosa	Subretinal injection	Immunocytochemistry Electroretinography (ERG) Western blot analysis	Over a 7-month period, mice transplanted with RPE-like cells showed a considerable recovery in their visual function, while mice injected with saline did not exhibit any rescue Electroretinogram responses in the transplanted eyes were elevated in one-fourth of the mice	[92]
Mice	Human iPSCs	Human keratinocytes	Immunodeficient *Crb1*^−/−^ mice a model of Retinitis pigmentosa	Subretinal transplantation	Histology Transmission electron microscope Immunocytochemical analysis Real-time RT-qPCR Western blot analysis	Following transplantation into 4-day-old immunodeficient Crb1 − / − mice, identifiable photoreceptor cells were formed both morphologically and immunohistochemically, indicating that the patient's mutations may function through post-developmental photoreceptor degeneration	[93]
Mice	Human embryonic and induced pluripotent stem cell-derived photoreceptor progenitors	Human	rd1 mice as a model of retinitis pigmentosa	Subretinal transplantation	Optomotor response Light avoidance response Histology Immunohistochemistry Real-time RT-qPCR	These cells established a cell layer linked to the host retinal neurons after differentiating into photoreceptors Two visual behavioral tests showed that the treated animals' visual function had partially returned	[94]
Mice	Human umbilical cord tissue mesenchymal stem cell-derived retinal progenitor cells	Human	rd12 mice as a model of retinitis pigmentosa	Intravitreal injection	Histopathology Immunohistochemistry Reverse transcription-PCR analysis Electroretinography	Significant improvements in function, retinal thickness, and vision were observed with transplanted RPCs Retinal pigment epithelium and pMSCs moved to the retina's neuronal layers, where they produced cell-specific markers and thickened the corresponding layers	[95]

**Table 5:** Summary of stem cells used in the treatment of glaucoma in different animals

Animal	Type of therapy	Source of stem cells	Type of injury	Site of injection	Clinical Evaluation	Clinical results	References
Rabbit	Human cord blood stem cells	Human cord blood	Lasered trabecular meshwork dysfunction	Intracameral injection	Histopathology Immunohistochemistry	Significant trabecular architecture damage, loss, and pleomorphism of trabecular endothelial cells, and progressive trabecular space narrowing until 12 weeks were seen in the rabbit eyes used for the laser-controlled experiment Conversely, endothelial cellularity and the structure of the trabecular meshwork were mostly retained in lasered eyes that were also infused with human cord blood stem cells	[96]
Rabbit	Human Wharton’s Jelly mesenchymal stromal cells (hWJ-MSCs)	Human	Glucocorticoid-induced ocular hypertension	Intravitreal injection	Analysis of the aqueous and vitreous humor IOP and ophthalmology changes Immunohistochemistry	hWJ-MSCs have the ability to regulate the immune system by increasing regulatory and suppressing proinflammatory cytokines Both the retina and the optic nerves showed significant expression of GFAP	[97]
Rat	Bone marrow stromal cells (BMSCs)	Rat femurs and tibias	Ocular hypertension was induced by cauterization of the episcleral veins	Intravitreal injection	Histology Immunohistochemistry Real-time RT-qPCR	Following transplantation, the inner limiting membrane and BMSCs were primarily visible, and very few cells had integrated into the ganglion cell layer When comparing the glaucomatous eyes with PBS injection to those with BMSC injection, the former revealed a smaller decrease in the number of retinal ganglion cells	[98]
Rat	Human Muller stem cell (MIO-M1)	Human neural retina	Laser-induced ocular hypertension	Intravitreal or subretinal transplantation	Quantification of ganglion cell axonal loss Immunohistochemistry	In vivo, transplanted cells persisted for 2 to 3 weeks, but by week 4, there was an invasion of macrophages and microglia and a decrease in graft survival The grafted cells were frequently directed towards the retina and expressed markers as glial fibrillary acidic protein	[99]
Rat	Bone marrow-derived mesenchymal stem cells	Rat femurs	Laser-induced ocular hypertensive glaucoma	Intravitreal or intravenous injection	Retinal ganglion cell Axon Quantification Immunohistochemistry Intraocular pressure analysis	Retinal ganglion cell axon survival generally increased, and the rate of RGC axon loss significantly decreased following intravitreal MSC transplantation The transplanted MSCs did not move to the damaged eye following intravenous treatment. Damage to the optic nerve was not affected by intravenous transplantation	[100]
Rat	Rat mesenchymal stem cells	Rat	Chronic ocular hypertension induced by laser cauterization of trabecular meshwork and episcleral veins	Intravitreal injection	Optic nerve quantification Retinal ganglion cell quantification Electroretinography Enzyme-linked immunosorbent assay (ELISA)	Evaluation of pupil light reflex and electroretinogram performance revealed that brain-derived neurotrophic factor -MSCs sustained much more retinal and optic nerve function than green fluorescent protein-MSC-treated eyes Comparing the eyes that received green fluorescent protein-MSCs alone versus those that got brain-derived neurotrophic factor-MSCs, the latter showed a higher degree of RGC preservation	[101]
Rat	Retinal stem cells (RSCs) combined with copolymer-1 immunization transplantation on interferon-gamma (IFN- γ)	Rat retina	Experimental glaucoma was induced by photocoagulation of the limbal plexus and episcleral veins with an argon laser	Intravitreal injection	Histological Analysis Immunohistochemistry Determination of IFN-γ by ELISA TUNEL assay for apoptosis	Apoptotic RGCs were less common in the RSC group than in the other groups, and animals that had glaucoma induction had higher IFN-concentrations in their serum and Aqueous Humour than non-induced control rats The RSCs group had considerably higher levels of BDNF and IGF-I expression than the other groups. Furthermore, the RSC group had significantly fewer apoptotic RGCs than the other groups did	[102, 103]
Rat	Bone marrow stem cells (BMSCs)	Rat femurs and tibias	Laser-induced ocular hypertension	Intravitreal injection	Visual water box performance Intraocular pressure monitoring	In the transplanted eye as opposed to the control eye, BMSCs increased retinal ganglion cells’ survival In tests of swimming directed by visual cues, the rats receiving BMSC transplants fared considerably better	[104]
Rat	Rat and human BMMC-derived factors	Rat femurs	Experimental ocular hypertension was induced by laser trabecular meshwork photocoagulation	Intravitreal injection	Histology Immunofluorescence	Retinal ganglion cell neuroprotection mediated by mesenchymal stem cells may be significantly aided by the production of platelet-derived growth factor, according to the numerous neuroprotective substances secreted by these cells	[105]
Rat	Bone-marrow mononuclear cells (BMMCs)	Rat femurs and tibias	Laser-induced model of open-angle glaucoma	Intraocular transplantation	Histology Immunofluorescence TUNEL assay for apoptosis	MSC-secreted substances stimulated cellular proliferation and reactivated a reservoir of progenitor cells within the ciliary body. For a minimum of 1 month, proliferating cells were seen inside the chamber angle Through laser-induced remodelling, MSCs might be directed into damaged sites, leading to targeted increases in ocular progenitor cells	[106]
Rat	Bone marrow mesenchymal stem cells, adipose-derived mesenchymal stromal cells	Rat bone marrow, adipose tissue	Ocular Hypertension induced using hyaluronic acid	Intravitreal transplantation	Retinal ganglion cell counting Intraocular pressure assessment Immunofluorescence	When comparing the stem cell-treated groups to the non-treated group, there was a substantial improvement in the number of retinal ganglion cells per area A small number of stem cells had integrated into the inner nuclear layer and the ganglion cell layer, according to the results of immunohistochemical tests	[107]
Rat	iPS cell-derived retinal ganglion cells	Mouse	Ocular hypertension	Intravitreal transplantation	Immunofluorescence RT-PCR analysis Electrophysiology	Induced RGCs showed the ability to make contact with particular targets and expressed axonal guidance molecules Moreover, these cells exhibited markers specific to RGCs and integrated into the host RGC layer when transplanted into the rat model of ocular hypertension	[108]
Rat	BMSCs	Rat femur	Ocular hypertension by cauterization of 3 episcleral veins	Injections were performed into the anterior chamber	Immunostaining Real-time qPCR Enzyme-linked immunosorbent assay (ELISA) Western blot analysis	MSC transplantation markedly lowered intraocular pressure in eyes that were hypertensive RGCs on the entire flat-mounted retina were counted, and the results showed that MSCs prevented RGC’s mortality	[109]
Rat	Adipose-derived stem cells, bone marrow-derived mesenchymal stem cells (BMSC), and dental pulp stem cells (DPSC)	Human	Ocular hypertension using transforming growth factor-β1 (TGF-β)	Intravitreal transplantation	Immunohistochemistry Optical coherence tomography (OCT) Electroretinography (ERG)	RGC numbers, RNFL thickness, and function were significantly reduced in control glaucomatous eyes that were sham-treated with heat-killed DPSC in comparison to intact eyes Significant protection against RGC loss, RNFL thinning, and preservation of RGC function was offered by BMSC and, to a larger extent, DPSC	[110]
Mice	Mouse embryonic stem cells (ESC)-derived neural progenitors	Mouse	Chemically induced ocular hypertension glaucoma model	Intravitreal injection	Behavioural analysis Immunohistochemistry Wheel running activity Whole mount retina immunostaining	After 2 months of transplantation, there was an improvement in visual acuity as compared to the pre-transplantation levels Improved vision could arise from transplanted cells surviving, differentiating into the retinal ganglion cell lineage, and perhaps integrating into the ganglionic cell layer	[111]
Mice	Mice iPSC were differentiated toward a TM cell phenotype (iPSC-TM)	Mice	Primary open-angle glaucoma (POAG)	The cells injected into the anterior chamber	IOP and aqueous humor outflow Transmission electron microscopy Western blot Immunohistochemistry	After 12 weeks of transplantation, iPSC-TM recipients had decreased IOP and enhanced outflow facility compared to untreated controls. Additionally, iPSC-TM transplantation preserves the ER structure	[112]

**Table 6:** Summary of stem cells used in the treatment of diabetic retinopathy

Animal	Type of therapy	Source of stem cells	Type of injury	Site of injection	Clinical evaluation	Clinical results	References
Rat	Human adipose-derived mesenchymal stem cells	Human	Diabetic retinopathy (DR) induced by streptozotocin	Intravenous injection	Assessment of Blood Glucose and blood-retinal barrier Immunohistochemistry	Blood glucose levels were significantly lower in the AMSC therapy group compared to the sham group after 1 week of transplantation In the retinas of the therapy group rats, donor cells were seen to display particular markers for astrocytes and photoreceptors, glial fibrillary acidic protein, and rhodopsin, respectively	[113]
Rat	Human umbilical cord blood-derived mesenchymal stem cells	Human umbilical cord blood	Diabetic retinopathy induced by streptozotocin	Intravitreal injection	Histology Immunohistochemistry	The control group exhibited diabetes retinopathy with increasing histological alterations. The MSC-treated group showed a significant increase in the number of ganglion cells with less clear regions Additionally, Prussian blue and CD34-positive cells were seen in several retinal layers of the MSC-treated group	[114]
Rat	Stromal fraction of adipose tissue	Human	Diabetic retinopathy (DR) induced by streptozotocin	Intravitreal injection	Histopathology Immunohistochemistry Confocal microscope Real-time RT-qPCR Electroretinogram TUNEL assay for apoptosis	Histopathological analysis showed that diabetic eyes receiving ASC had significantly less vascular leakage and apoptotic cells surrounding the retinal arteries than eyes receiving saline injection Furthermore, diabetic retinas treated with ASC exhibited a down-regulation in the expression of inflammatory genes by molecular studies	[115]
Rat	Bone marrow-derived stem cells (BMSC)	Rat femur and tibia	Diabetic retinopathy (DR) induced by streptozotocin	Intravitreal injection	Immunohistochemistry Immunofluorescence Electroretinography	Vimentin and GFAP expression measurements revealed that the diabetic group had more retinal gliosis than the baseline group The oscillatory potential wave amplitudes of the diabetic group were significantly lower than those of the Baseline group	[116]
Rat	Perivascular progenitor cell-derived from human embryonic stem cells (hESC-PVPCs)	Human	Streptozotocin-induced diabetic model	Intravitreal injection	Retinal vascular image analysis Microarray analysis Immunofluorescence Real-time RT-qPCR	Cells stabilized the breach of the blood–retinal barrier by localizing beside typical perivascular areas of the retinal vasculature The therapeutic potential of hESC-PVPCs in diabetic retinopathy by imitating pericytes' function in vascular stabilization	[117]
Rat	Adipose tissue-derived stem cells	Rat adipose tissue	Diabetic retinopathy (DR) induced by streptozotocin	Intravenous injection through the tail vein	Histopathology Immunohistochemistry Real-time RT-qPCR	In contrast to a large drop in C-peptide and HDL levels, the STZ injection considerably raises blood glucose, HbA1c, cholesterol, TG, and LDL levels With the use of adipose tissue stem cell therapy, all of these metrics improved. When compared to the usual control group, this improvement fell short of expectations	[118]
Rat	Neural stem cells differentiated from human umbilical cord-derived mesenchymal stem cells	Human	Diabetic retinopathy-induced by streptozotocin	Intravitreal injection	Histology Immunohistochemistry Electroretinogram Western blot analysis	Therapy reduced retinal vascular dysfunction in rats as compared to rats that were not treated, and treatment significantly increased the expression of Thy-1 and BDNF in the treated group compared to the control group	[119]
Rat	Bone marrow-derived mesenchymal stem cells	Rat femurs and tibias	Diabetic retinopathy induced by streptozotocin	Intravitreal injection	Measurement of blood glucose Measurement of retinal oxidant/antioxidant redox Histopathology Immunohistochemistry Enzyme-linked immunosorbent assay	Restoring the retinal oxidative/antioxidant redox and lowering retinal inflammatory mediators were the results of melatonin and/or stem cell treatment When melatonin and stem cells were administered together, the amount of transplanted stem cells in the retinal tissue increased, and the levels of retinal BDEF, VEGF, APOA1, and RBP4 were much lower than in other groups	[120]
Rat	Human umbilical cord blood-derived mesenchymal stem cells (hUCB-MSCs) pretreated with Sirolimus	Human	Diabetic retinopathy (DR) induced by streptozotocin	Subconjunctival injection	Assessment of body weight and blood glucose Histology Electroretinography	Histological analysis demonstrated that the retinal layers in the DR-induced groups were thinner than those in the control group, with the DR-MSC-S group exhibiting the thickest retinal layers The DR-MSC and DR-MSC-S groups exhibited considerably higher values than the DR group, according to the flicker amplitude measurements	[121]
Rat	Bone marrow-derived mesenchymal stem cells	Rat femurs and tibias	Diabetic retinopathy induced by streptozotocin	Intravitreal injection	Histopathology Immunohistochemistry Real-time RT-qPCR	When compared to the control group, morphometric examination of the MSC-treated group showed a statistically significant increase in retinal thickness and a decrease in CD34 and fibronectin immunoreaction optical densities	[122]
Mice	HASC-derived pericyte	Human adipose tissue	Diabetic retinopathy Oxygen-induced retinopathy (OIR)	Intravitreal injection	Confocal microscope for retinal imaging Immunohistochemistry	ASCs improve retinal microvascular stabilization in three different pre-clinical mouse models of retinopathic vasculopathy when injected intravitreally Pericytes produced from ASCs can integrate with the retinal vasculature, taking on the shape and marker expression of pericytes, and offering protective functions for the vasculature	[123]
Mice	Adipose-derived stem cells from healthy and diabetic mice	Mice epididymal fat	Akimba mouse model of diabetic retinopathy	Intravitreal injection	Immunofluorescence Confocal microscopy TUNEL assay for apoptosis	Protecting the diabetic retina against further vascular dropout was a greater efficacy of healthy mouse ASCs than diabetic ASCs As demonstrated by a high-throughput enzyme-linked immunosorbent test, diabetic ASCs released fewer vasoprotective substances than healthy ASCs	[124]
Mice	Adipose-derived MSCs	Mice epididymal fat	Diabetic mice using streptozotocin	Intravitreal injection	Immunofluorescence Real-time qPCR Enzyme-linked immunosorbent assay (ELISA) Angiography Electroretinography	Retinal ganglion cell loss was totally stopped by MSC injection The level of oxidative damage in the retina was decreased and the intraocular concentrations of many powerful neurotrophic factors (glial cell line-derived neurotrophic factor, basic fibroblast growth factor, and nerve growth factor) improved	[125]
Mice	Human adipose-derived stem cells (ASCs) positive for the pericyte marker CD140b	Human adipose tissue	Ins2^Akita^ mouse model of Diabetic Retinopathy	Intravitreal injection	Histological evaluation Immunohistochemistry Flash electroretinography (ERG) Fluorescein angiography and retinal fundus imaging Optical coherence tomography (OCT) Gene expression analysis	By modifying the neurovascular system, a single intravitreal injection of ASCs can therapeutically enhance the retina and improve vision In comparison to unstimulated cells, cytokine-primed ASC-CM exhibits differential production of chemokines and angiogenic proteins ASCs or their secreted factors reduce the retinal problems caused by diabetes	[126]
Mice	Bone Marrow CD133^+^ stem Cells	Mice femurs and tibias	Diabetic retinopathy induced by streptozotocin	Intravitreal injection	Visual acuity Immunofluorescence Electroretinography Real-time RT-qPCR Western blot analysis	Functional investigation showed that for 56 days, the transplantation of CD133 + cells avoided visual impairment Histological examination verified this functional improvement and demonstrated that transplanted CD133 + cells endured, eventually moved into the inner retina, and maintained IR degeneration, including rod-on bipolar cells and retina ganglion cells	[127]
Mice	Human CD34 + bone marrow stem cells	Human	Diabetic retinopathy induced by streptozotocin	Intravitreal injection	Confocal microscopy imaging Immunohistochemistry	In the superficial retinal capillary plexus, the eyes that were injected intravitreally with CD34 + BMSCs exhibited notably greater vascular density and vascular length density in comparison to the untreated contralateral eye or the PBS-treated control eye	[128]

**Table 7:** Summary of stem cells used in the treatment of retinal ischemia

Animal	Type of therapy	Source of stem cells	Type of injury	Site of injection	Clinical evaluation	Clinical results	References
Rat	Bone marrow-derived mesenchymal stem cells	Rat femurs and tibias	Retinal ischemia/reperfusion model	Intravitreal injection	Immunocytochemistry Confocal microscopy Real-time RT-qPCR Western blot analysis	A few BMSCs were seen to exhibit neurotrophic factors, neurofilament, and neuron-specific enolase two to four weeks after transplantation Compared to the eyes treated with PBS injection, the eyes treated with BMSC exhibited a smaller decrease in the quantity of retinal ganglion cells	[129]
Rat	(BMSC)-conditioned medium	Rat femur	Retinal ischemia by increasing intraocular pressure to 130 to 135 mm Hg for 55 min	Intravitreal injection of conditioned medium 24 h after ischemia	Histology Electroretinography Fluorescent TUNEL Mass spectrometry Western blotting	Intravitreal injection of conditioned medium 24 h after ischemia significantly improved retinal function and attenuated cell loss in the retinal ganglion cell layer Conditioned medium attenuated postischemic apoptosis and apoptosis-related gene expression Conditioned medium is a robust means of delayed postischemic intervention	[130]
Rat	(BMSC)-conditioned medium	Rat femur	Retinal ischemia by increasing intraocular pressure to 130 to 135 mm Hg for 55 min	Medium was injected into the vitreous 24 h after ischemia ended	Histology Electroretinography Fluorescent TUNEL ELISA rat cytokine array	When eyes were injected with hypoxia BMSC-conditioned medium 24 h after ischemia, they showed considerably improved recovery of retinal function, decreased retinal ganglion cell layer loss, and attenuated apoptosis compared to those given normoxic or hypoxic unconditioned medium. Protein levels in the hypoxic-preconditioned medium were substantially higher than those in the normoxic medium	[131]
Rat	BMSCs	Rat femur	Retinal ischemia was generated by increasing intraocular pressure (IOP) to 130–135 mmHg for 55 min	BMSCs were injected into the vitreous 24 h post-ischemia	Electroretinography Fluorescent TUNEL Immunohistochemistry Western blotting	The recovery of the ERG a- and b-waves, OP, negative STR, and P2 was markedly enhanced by the intravitreal injection of BMSCs, which also attenuated apoptosis as shown by a decrease in TUNEL and caspase-3 protein levels Retinal vascular permeability was dramatically reduced, autophagy was promoted, and inflammatory mediators (TNF-α, IL-1β, and IL-6) were decreased by BMSCs. BMSCs were present in the ischemic retina as well as the vitreous	[132]
Rat	MSCS	Rat	Middle cerebral artery occlusion (MCAO)	Intravenous	Immune histochemistry Cell viability assay Laser Doppler blood flow measurement	At days 3 and 14 post-stroke, middle cerebral artery closure drastically reduced blood supply to the brain and eye, as well as mitochondrial malfunction and ganglion cell death Intravenous MSCs stimulated mitochondrial repair and increased ganglion cell survival on day 14 after stroke	[133]
Mice	BM-derived myeloid progenitor cells	Mice femurs and tibias	Oxygen-induced retinopathy	Intravitreal injection	Immunohistochemistry Electroretinography	With no long-term toxicity detected, transplanted BM-derived progenitors significantly sped up the retinal vascular repair of OIR, boosting the pace of physiological intraretinal revascularization while also significantly lowering the production of aberrant, preretinal neovascularization	[134]
Mice	Human cord blood–induced pluripotent stem cells (CB-iPSCs)-derived vascular progenitors	Human	Retinal ischemia–reperfusion model	Intravitreal injection	Immunofluorescence Real-time RT-qPCR Western blot analysis	Vascular Progenitors produced from human embryonic stem cells and CB-iPSCs engrafted and homed into injured retinal capillaries with reliability, staying inside the damaged vasculature for up to 45 days	[135]
Mice	Mice bone marrow-derived lineage-negative (lin-ve) stem cells	Femur, tibia, humerus, and radio-ulna of mice	Pterygopalatine artery ligation induced retinal ischemia–reperfusion injury	Intravenously through tail vein after 24 h of injury	Histology immunohistochemistry Fundus fluorescein angiography Laser Doppler FITC-dextran imaging Electroretinography Real-time PCR	The retina showed enhanced expression of neurotrophic factors such as BDNF and FGF2, but decreased expression of GFAP The functional examination using an Electroretinogram indicated no significant alterations before or after injury or stem cell implantation	[136]

**Table 8:** Summary of stem cells used in the treatment of Uveitis, retinal detachment, and optic neuropathies

Animal	Type of therapy	Source of stem cells	Type of injury	Site of injection	Clinical evaluation	Clinical results	References
Mice	Bone marrow mesenchymal stem cells (MSCs)	Mice femurs and tibiae	Experimental autoimmune uveitis	Intraperitoneal injection	Delayed-type hypersensitivity assay Quantitative RT-PCR	Experimental auto-immune uveitis was found to be much lessened by injection of MSCs The paracrine production of antigen-specific Treg by MSCs through the secretion of TGF-B is at least partially responsible for their immunomodulatory role, as demonstrated by their ability to suppress experimental auto-immune uveitis	[137]
Mice	Gingiva-derived mesenchymal stem cells (GMSCs)	Human gingiva	Experimental autoimmune uveitis	The cells were injected into tail vein	Gene ontology enrichment analysis Intercellular communication analysis RNA-sequencing	GMSC significantly rescued monocytes, dendritic cells, T cells, and B cells. T helper 17 cell proportion was restored, and regulatory T cell proportion was raised GMSCs had a significant impact on Th17 cell morphologies, increasing the production of interleukin 10 in the CCR6 + CCR2 + phenotype and inhibiting the formation of the highly inflammatory CCR6-CCR2 + phenotype	[138]
Mice	Human-induced pluripotent stem cell	Human	Experimental autoimmune uveitis (EAU)	Intravenous injection through the caudal vein	Fluorescein fundus angiography Optical coherence tomography Histology Western blot analysis Immunofluorescence Real-time RT-qPCR	The number of all retinal neuron types and their marker expression levels decreased as a result of EAU, and Müller glia may have served as antigen-presenting cells in this process Additionally, there was a significant increase of the chemokine CCL5 in the EAU retinas due to the classical EAU generated by the interphotoreceptor retinoid-binding protein peptide	[139]
Rat	BMSCs	Rat femurs and tibias	Retinal detachment	Subretinal transplantation	Cell viability assay Caspase activity assays TUNEL assay Histology Immunohistochemistry Western blot analysis	Retinal autophagy was triggered, and apoptosis was decreased in the retinas treated with BMSCs following transplantation An increase in autophagy during the early stages may help cells survive hypoxic stress Transplanting BMSCs can drastically reduce photoreceptor cell loss and preserve retinal integrity in retinal detachment models	[140]
Rat	Umbilical cord blood mesenchymal stromal cells	Human	Optic tract transection model	Cells were then implanted onto a piece of gel foam at the lesion site	Immunofluorescence Enzyme-linked immunosorbent assay (ELISA)	Retrograde tracer studies revealed that transplanted MSCs and human fibroblast line cells had a neuroprotective effect four weeks after grafting, saving a sizable portion of axotomized retinal ganglion cells (RGCs) Moreover, MSCs may encourage the regrowth of axotomized RGCs to the superior colliculus, their intended target	[141]
Rat	Neurotrophic factors secreting Bone marrow mesenchymal stem cells (NTF-SCs)	Rat and human	Optic nerve transection (ONT)	Intravitreal injection	Immunohistochemistry Real-time RT-qPCR Enzyme-linked immunosorbent assay (ELISA)	Rat NTF-SCs generated from bone marrow did not exhibit the same level of neuroprotection as human NTF-SCs compared to PBS. Following an intravitreal injection Immunohistochemistry showed that human-derived MSCs, human NTF-SCs, and rat-derived NTF-SCs all survived for at least 24 days	[142]
Rat	Human umbilical cord blood stem cells (hUCBSCs)	Human	Optic nerve crush	Intravitreal injection	Histology Real-time RT-qPCR	In comparison to the model group, the transplanted group exhibited a significant amelioration of pathological injury, as evidenced by the clear increase in the number of labelled retinal ganglion cells and the expression of BDNF and GDNF mRNA	[143]
Rat	Bone-marrow mononuclear cells (BMMCs)	Rat femurs and tibias	Optic nerve crush	Intravitreal injection	Immunohistochemistry Real-time RT-qPCR Analysis of axonal outgrowth to the brain	The rats treated with BMMC also exhibited decreased Müller glia activation and more axons, growing up to 1.5 mm from the crush site The mRNA levels of fibroblast growth factor 2 increased in treated mice 14 days after injury, according to an analysis of mRNAs under all conditions	[144]
Rat	Rat bone marrow mesenchymal stem cells (rMSCs) with rat BDNF	Rat bone marrow	Optic nerve axotomy	Intravitreal or subretinal injection	Immunohistochemistry Real-time RT-qPCR Western blot analysis	Up until 4 weeks following transplantation, the subretinal injection of MSCs markedly elevated the expression of BDNF in the retina of axotomized rats Compared to intravitreal injection, subretinal injection resulted in a substantially higher level of BDNF expression in the retina and more extensive stem cell integration	[145]
Rat	Bone-marrow mononuclear cells (BMMCs)	Rat femurs and tibias	Optic nerve crush	Intravitreal injection	Immunohistochemistry Real-time RT-qPCR Western blot analysis	Cell treatment greatly increases the strong expression of Tax1-binding protein 1 in the ganglion cell layer In the retina and optic nerve, activated Müller cells and astrocytes express Synaptotagmin IV, and there are no differences in the protein levels between the two populations	[146]
Rat	Human umbilical cord blood-derived mesenchymal stem cells	Human umbilical cord blood	Acute optic nerve injury	Intravitreal injection	Histology Immunohistochemistry Real-time RT-qPCR Western blot analysis TUNEL assay for apoptosis	MSC implantation dramatically lowered cellular apoptosis and increased the survival of retinal ganglion cells in the early phase However, this protection was transitory, and the ganglion cells could not be protected from death in the end	[147]
Rat	Human umbilical cord blood stem cells (hUCBSCs)	Human	Optic nerve injury	Intravitreal injection	Histology Real-time RT-qPCR Measurement of optic nerve function TUNEL assay for apoptosis	Compared to the injured group, transplantation of hUCBSCs dramatically attenuated a decline in optic nerve function as seen by reduced amplitude declines and peak latency increases of the wave form Significant increase in the number of retinal ganglion cells (RGCs) was seen following transplantation	[148]
Rat	Dental pulp stem cells (DPSCs) and bone marrow-derived mesenchymal stem cells (BMSCs)	Rat dental pulp, femurs	Optic Nerve Injury	Intravitreal injection	Immunohistochemistry Optical coherence tomography	Compared to BMSCs, DPSCs secrete more neurotrophins, which aid in RGC survival and axon regeneration NGF, BDNF, and NT-3 were secreted by both DPSCs and BMSCs; however, DPSCs released noticeably larger titters of NGF and BDNF than BMSCs	[149]
Rat	Bone-marrow mononuclear cells (BMMCs)	Rat femurs and tibias	Optic nerve crush	Intravitreal injection	Immunohistochemistry Retinal Ganglion Cells survival analysis Magnetic resonance imaging (MRI)	At 16 days following optic nerve crush, cell treatment produced a considerable increase in the number of axons distal to the crush site, but the number of retinal ganglion cells declined with time 28 days following injury, we showed a 5.2-fold increase in axon outgrowth; however, BMMCs had no effect on RGC survival Two BMMC injections are administered as part of a new regimen, the second of which is given seven days after the injury, in an effort to increase RGC survival	[150, 151]
Rat	Bone marrow–derived mesenchymal stem cells	Rat and human	Optic nerve crush	Intravitreal injection	Axon count Immunofluorescence Western blot analysis	In the PBS-treated group, there were relatively few axons that extended past the crush point On day 15 following injury, a significant proportion of axons in the BMSC-treated group passed through the crush site Thus, the regeneration of injured axons was markedly enhanced by BMSCs	[152]
Rat	Human periodontal ligament-derived stem cells (PDLSCs)	Human teeth	Optic nerve crush (ONC)	Intravitreal injection	Immunofluorescence Real-time RT-qPCR Enzyme-linked immunosorbent assay (ELISA)	Three weeks after ONC, human PDLSCs were still alive in the vitreous chamber and were sustained on the RGC layer Rats transplanted with human PDLSCs had considerably higher numbers of surviving RGCs and regenerating axons	[153]
Mice	Embryonic-derived retinal stem cells	Mice embryonic retina	Optic nerve crush	Intravitreal injection	Histopathology Immunohistochemistry Electroretinography	In addition to reduced amplitudes of the a, b waves in the ERG, the injured retina displayed cellular nuclear disintegration and fragmentation in the retinal tissue that developed over the clamping times RSCs moved into the inner nuclear layer and retinal ganglion cell layer. In retinal injuries, transplanted RSCs markedly increased wave amplitudes	[154]

**Table 9:** Summary of stem cells used in the treatment of other retinal degenerative diseases

Animal	Type of therapy	Source of stem cells	Type of injury	Site of injection	Clinical evaluation	Clinical results	References
Feline	Human Muller glia with stem cell (hMGSC)-derived retinal ganglion cell (RGC)	Feline retina	Retinal ganglion cell (RGC) depletion	Intravitreal injection	Histopathology Immunofluorescence analysis Reverse transcription-PCR analysis Western blotting analysis Electroretinography	Retinal function was improved, and cell attachment was encouraged by allogeneic transplantation of RGC generated from Muller glia, as demonstrated by an improvement in the electroretinogram’s threshold responses According to the findings, successful neuroprotection requires the transplanted cells to adhere to the retina for RGC function to improve	[155]
Rabbit	Human retinal pigment epithelium Stem Cells (hRPESC) grown on polyester membranes	Human	Two-port core-vitrectomy	Subretinal transplantation	Histology Electron microscopy Immunohistochemistry	Retinal atrophy covering the foetal or adult transplant was noticed after one week, and it remained constant after that Four weeks following implantation, histology revealed a continuous, polarised human RPE monolayer on polyester	[156]
Rat	Embryonic stem cell-derived pigment epithelial cells (ESPEs)	Monkey	Royal College of Surgeons (RCS) rats as a model of retinal degeneration	Subretinal injection	Behavioral assessment Histologic analysis Immunohistochemistry Transmission electron microscopy RT-PCR analysis Western blot analysis	The RPE markers ZO-1, RPE65, CRALBP, and Mertk were expressed by the ESPEs. Grafted epidermal growth factors (ESPEs) improved host photoreceptor survival when inserted into the subretinal space of RCS rats	[157]
Rat	Human Embryonic Stem Cells	Human	Royal College of Surgeons (RCS) rats as a model of retinal degeneration	subretinal injections	Histologic analysis RT-PCR analysis Electroretinogram	After being transplanted into RCS rats, the cells lived for a long time and migrated to the subretinal space rather than the retina Animals treated with hESC-derived RPE not only demonstrated broad photoreceptor rescue (5–7 cells deep in the outer nuclear layer), but also outperformed sham and untreated controls significantly in terms of relative sharpness as determined by the Optomotor system	[158]
Rat	Human umbilical cord tissue-derived cells, human placental-derived cells, and human bone marrow mesenchymal stem cells	Human	Royal College of Surgeons rats are used as a model of retinal degeneration	Subretinal transplantation	Histology Electroretinogram Acuity threshold Luminance threshold	Large areas of photoreceptor recovery were provided by cells generated from umbilical tissue, whereas mesenchymal stem cells only provided localised rescue. Placental cells had a similar effect to controls, but not significantly better The cells produced from umbilical tissue showed the strongest photoreceptor restoration	[159]
Rat	Bone marrow mesenchymal stem cells (BMSCs)	Mice	Royal College of Surgeons (RCS) rats as a model of retinal degeneration	Subretinal transplantation	Histology Quantitative RT-PCR Electrophysiology	MSC subretinal implantation prevents and slows the aging of the retina while maintaining retinal function These findings imply that MSC can be a valuable source of cells for treatments involving cell replacement for some types of retinal degeneration	[160]
Rat	Human iPS cell-derived retinal pigmented epithelium	Human	Royal College of Surgeons rats as a model of retinal dystrophy	Subretinal transplantation	Immunohistochemistry Electron microscope Real-time RT-qPCR Western blot analysis	Transplanting iPS cells can help sustain photoreceptors in the near term by phagocytosing photoreceptor outer segments The preservation of long-term visual function in this retinal illness model is indicative of a secondary protective host cellular response, even in cases where the xenografted cells eventually die	[161]
Rat	Human embryonic stem cell-derived retinal pigment epithelium hESC- RPE	Human	Royal College of Surgeons (RCS) rats as a model of Age-related macular degeneration	Subretinal injection	Histologic analysis Immunocytofluorescence RT-PCR analysis Western blot analysis Electroretinography	The cells maintained photoreceptor integrity and visual function without becoming teratoma or experiencing undesirable pathological responses The effective treatment of many retinal degenerative diseases may benefit from the use of hESCs as a potentially safe and limitless supply of RPE	[162]
Rat	Human adult bone marrow-derived somatic cells	Human	Royal College of Surgeons (RCS) rats as a model of retinal degeneration	Subretinal transplantation	Histology Functional assessment Antibody staining	Histological analysis showed that while sham-injected and untreated controls had only one layer of photoreceptors, eyes receiving cell injections had three to six layers	[163]
Rat	Bone marrow-derived mesenchymal stem cells	Rat femurs and tibias	Retinal light-damaged model	Subretinal transplantation	Histology Immunohistochemistry TUNEL assay for apoptosis	The group that received BMSC transplantation had a much lower percentage of apoptotic outer nuclear layer cells than the groups that received light damage or phosphate-buffered solution injection In comparison to the group that received light damage, the retinas of the BMSC transplantation group showed higher levels of basic fibroblast growth factor and BDNF immunoreactivity	[164]
Rat	BMSCs	Rat femurs and tibias	Laser-induced retinal trauma model	The cells were injected into the tail vein	Histopathology Fundus photography Confocal microscope images	In the experimental group, retinal detachment improved, while in the control group, it got worse Five weeks following the MSC injection, the retinotomy sites partially healed with discernible cells Seven weeks following the MSC injection, the transitional zone between the injured and normal retina showed abundant cells and full repair without retinal detachment	[165]
Rat	Human iPS cell-derived retinal pigmented epithelium	Human	Nude rats as a model of age-related macular degeneration	Subretinal transplantation and subcutaneous	Histology Immunohistochemistry Real-time RT-qPCR	After 6 to 12 months of observation, no tumor was discovered using RPE sheets produced from iPSCs	[166]
Rat	Human adult bone marrow-derived stem cells (hBM-MSCs)	Human	Royal College Surgeon rat model of retinal degeneration	Subretinal or intravitreal transplantation	Histology Immunohistochemistry Immunofluorescence Electroretinogram	Up to 20 weeks after transplantation, retinal function in the subretinal group was considerably better in transplanted eyes than in control eyes. In contrast, restoration of retinal function was only sustained for 12 weeks after transplantation in the intravitreal group hBMMSCs were dispersed throughout the majority of the subretinal space and choroid in the subretinal group as a nearly uniform thin layer. While concentrated in the vitreous cavity of the intravitreal injection group	[167]
Rat	Human Wharton’s Jelly-derived mesenchymal stem cells (hWJ-MSCs)	Human	Royal College of Surgeons (RCS) rats as a model of retinal degeneration	Subretinal injection	Micro-computed tomography Electroretinography Histological analysis Transmission electron microscopy	hWJ-MSCs remained localised in the eye and did not migrate systemically, according to micro CT scans Histology revealed that the treated group had the outer nuclear layer preserved, whereas the control group did not MSCs were shown to express markers for Müller cells, bipolar, and photoreceptors using confocal imaging	[168]
Rat	Human bone marrow mesenchymal stromal cells (hBM-MSCs)	Human	Royal College Surgeon rats model of retinal degeneration	Subretinal transplantation	Histology Optical coherence tomography Immunofluorescence Electroretinogram	A thin coating of transplanted cells was seen throughout the choroid’s extravascular spaces and subretinal Cell transplantation markedly improved retinal functioning and postponed photoreceptor degradation throughout the retina in RCS rats After transplantation, there were no signs of choroidal haemorrhages or retinal detachment in the rabbits	[169]
Rat	Human bone marrow mesenchymal stem cells	Human	Royal College of Surgeons (RCS) rats as a model of retinal degeneration	Epiretinal transplantation	Histology immunofluorescence Electroretinogram	Up to 20 weeks after cell transplantation, retinal function was restored and photoreceptor degradation was postponed by epiretinal surgery Following epiretinal transplanting, rats’ visual functions stayed relatively normal. Transplanted eyes showed no signs of inflammation or other negative side effects	[170]
Rat	Retinal pigmented epithelium stem cell (RPESC)-derived RPE cells (RPESC-RPE)	Human	Rat model of RPE cell dysfunction	Subretinal transplantation	Assessment of Vision Phagocytosis assay Histology Immunocytochemistry	The effectiveness of RPE cell replacement is highly influenced by the maturation stage of RPESC-RPE After four weeks of culture, an intermediate stage of RPESC-RPE differentiation was more constant at eyesight rescue Preserving visual behavior in RCS rats is more effective with an intermediate 4-week RPESC-RPE stage	[171]
Rat	Human umbilical cord blood-derived mesenchymal stem cells	Human umbilical cord blood	Vigabatrin-induced retinopathy	Intravenous injection through tail vein	Histopathology Immunohistochemistry Real-time RT-qPCR	Following Vigabatrin administration, MSCs reduced the expression of glial fibrillary acidic protein, and vascular endothelial growth factor, ameliorating retinal degenerative alterations and indicating the function and vascular modifying effect of MSCs Furthermore, MSCs control the expression of genes related to BDNF, NGF, and interleukin in retinal tissue	[172]
Rat	(ADSCs) (BMSCs) Amniotic fluid stem cells (hAFSCs) Dental pulp stem cells (hDPSCs) Induced pluripotent stem cell (hiPSC) hiPSC-derived retinal pigment epithelium (RPE)	Human	Royal College of Surgeons (RCS) rats as a model of retinal degeneration	The cells were delivered into the subretinal space	Light–dark box evaluation Optomotor response evaluation Fundus photography Histological analysis Electroretinography Enzyme-linked immunosorbent assay (ELISA) Immunofluorescence analysis	Adult and fetal stem cells produced improvements in visual function for up to 4 weeks after injection in comparison to hiPSC-derived RPE cells The thickness of the outer nuclear layer in histological sections displayed a pattern consistent with the findings of the qOMR and ERG When any type of stem cell, other than hiPSCs, was transplanted into the subretinal cavity of RCS rats, their ERG waves were higher than those of the control rats	[173]
Mice	Adult bone marrow–derived lineage-negative hematopoietic stem cells	Mice bone marrow	rd1 and rd10 models of retinal degeneration	Intravitreal injection	Immunofluorescence Quantitative RT-PCR Electroretinography	Numerous antiapoptotic genes, including transcription factors and minor heat shock proteins, are significantly upregulated in rescued retinas according to microarray analysis	[174]
Mice	Embryonic stem cell-derived eye-like structures	Mouse	N-Methyl-D-aspartate (NMDA)-Induced retinal damage	Intravitreal injection	Histology Immunohistochemistry TUNEL assay for apoptosis Electrophysiology	After transplanting the embryonic stem cell-derived eye-like structures into eyes that had received NMDA treatment, the cells covered a sizable portion of the host retinal ganglion cell layer that had been damaged by the NMDA and dispersed throughout the retina Also, the cells developed into retinal ganglion cell-specific marker-expressing cells, forming a new retinal ganglion cell layer	[175]
Mice	BM-derived MSCs	Mice femurs and tibias	Laser-induced Choroidal neovascularization (CNV) model	Intravenous injection through tail vein	Histology Immunofluorescence Enzyme-linked immunosorbent assay (ELISA)	The antiangiogenic pigment epithelial-derived factor (PEDF), which is produced at the locations of CNVs by MSCs, inhibits the expansion of CNVs and promotes regressive characteristics	[176]
Mice	Mouse-induced pluripotent stem cells (iPSCs)	Mouse	Rhodopsin-null mice as a model of retinal degeneration	Subretinal injections	Immunocytochemical analysis RT-PCR Analysis Electroretinogram	iPSCs colonized the outer nuclear layer of the retina and produced enhanced electro retinal function as measured by ERG and functional anatomy To produce retinal precursors for transplantation for the therapy of retinal degenerative diseases, adult fibroblast-derived iPSCs offer a feasible source	[177]
Mice	Human embryonic stem cells (hESC) differentiate into retinal progenitor cells (hESC-RPCs)	Human embryo	Un-immunosuppressed mouse retina	Subretinal and Epiretinal Transplantation	Histological analysis Immunohistochemistry RT-PCR Analysis	When the transplantation process did not compromise the blood–retinal barrier, the cells in the subretinal grafts developed and endured in a xenogeneic environment without immunosuppression Although the epiretinal grafts lived, they lacked mature retinal cell markers Epiretinal grafts are efficiently integrated into the inner nuclear layer (INL) and the layer of retinal ganglion cells (RGCs), but not the subretinal layer	[178]
Mice	Embryonic stem cell-derived photoreceptor precursors	Mouse	Adult Gnat1 − / − mouse, a model of night blindness,	Subretinal injection	Immunohistochemical analysis RT-PCR analysis Electrophysiology	Following this technique, rod precursors develop into outer segment-bearing photoreceptors that integrate inside the degenerate retinas of adult mice For retinal cell transplantation, ESCs can supply a source of photoreceptors	[179]
Mice	3D-differentiated ESC- or iPSC-derived retinal sheets	Mouse	rd1 mice as a model of retinal degeneration	Subretinal Transplantation	Immunohistochemistry Electron microscope analysis Real-time RT-qPCR Fluorescence-activated cell sorting analysis	The transplanted sheets flourished in the host retina, eventually developing into mature photoreceptors' organised outer nuclear layer The transplanted retinal sheets photoreceptors of the structured outer nuclear layer may establish direct synaptic connections with the host bipolar cells	[180]
Mice	BMSCs	Mice	Mice-induced reactive gliosis as a model of retinal ganglion cells loss	Intravitreal injection	Immunohistochemistry Real-time RT-qPCR Western blot analysis	Intravitreal BMSCs transplantation is linked to the recruitment of macrophages, elevation of intermediate filaments, and retinal folding mediated by gliosis Glial fibrillary acidic protein production in retinal Muller glia was successfully decreased and BM-MSC retinal engraftment was boosted by pharmacologically inhibiting STAT3 in BMSCs cocultured retinal explants	[181]
Mice	Human mesenchymal stromal cells Human neural stem cells Human iPS-derived retinal pigmented epithelial cells	Human	rd1 mice as an animal model of retinitis degeneration	Subretinal injection	Immunofluorescence analysis Gene expression analysis Enzyme-linked immunosorbent assay (ELISA)	Compared to the other two types of transplant cells, which had lower immune responses and apoptosis, human iPS-RPE cells dramatically reduced photoreceptor degradation Because they can survive in a degenerating ocular environment, iPS-RPE cells are a viable source for delaying photoreceptor degeneration	[182]
Mice	Human umbilical tissue-derived cells (hUTC)	Human umbilical tissue	Royal College of Surgeons (RCS) rats as a model of retinal degeneration	Subretinal injection	Immunofluorescence analysis Gene Expression analysis Enzyme-linked immunosorbent assay (ELISA)	Through the production of receptor tyrosine kinase ligands and bridge molecules, hUTC can effectively treat RPE phagocytic dysfunction The receptor tyrosine kinase (RTK) ligands secreted by hUTC include brain-derived neurotrophic factor (BDNF), hepatocyte growth factor (HGF), and glial cell-derived neurotrophic factor (GDNF)	[183]
Mice	Neural stem cells	Mice	rd1 mice as an animal model of retinitis degeneration	The cells injected into the subretinal space	Immunofluorescence Enzyme-linked immunosorbent assay Real-time quantitative polymerase chain reaction Western blot analysis	The transplantation of NSCs into the subretinal space of rd1 animals resulted in the inhibition of activated microglia and a delay in the degeneration of the outer nuclear layer Tissue inhibitor of metalloproteinase (TIMP1) was shown to have an increased gene and protein level in NSCs, while matrix metalloproteinase (MMP9) was found to be significantly reduced in BV2 microglia	[184]
Mice	Human BM CD34 cells	Human bone marrow	rd1 mutation mice as an animal model of retinitis degeneration	Intravitreal injection	Scanning laser ophthalmoscopy Optical coherence tomography Electroretinography Immunohistochemistry	In all eyes, electroretinography testing revealed a flat signal both one and four weeks after injection More than 300 mouse genes, mostly involved in photoreceptor maintenance and function as well as apoptosis, were found to have changed in expression in the retina after cell injection, according to microarray analysis	[185]
Mice	Embryonic stem cell-derived cone precursors	Mouse	Aipl1 − / − mice, a model of end-stage retinal degeneration	Subretinal injection	Immunohistochemistry Fluorescence-activated cell sorting	Cone cell replacement research can benefit from the use of mESC-derived retinal organoids as a developmentally appropriate donor source In the subretinal space of Aipl1 − / − mice, a model of end-stage retinal degeneration, MESC-derived cones may be separated in great quantities and transplanted into adult mouse eyes, demonstrating the ability to live and mature	[186]
Mice	Mouse iPSC-derived retinal tissue (miPSC retina)	Mouse	rd1 mice as an animal model of retinitis degeneration	Subretinal transplantation	Behavioral assessment Histological analysis RT-PCR analysis Electroretinogram	When iPSC retina is implanted into the eyes of mice with end-stage retinal degeneration, it matures into an ONL and reacts to light The majority of the mice that had retinal transplants exhibited behavior in response to light	[187]
Mice	Purified human pluripotent stem cell-derived cone photoreceptors	Human	rd1 mice as an animal model of retinitis degeneration	The cells transplanted into damaged retina	Immunohistochemistry Image analysis Transmission electron microscopy (TEM) Multi-electrode array (MEA)	Assessments based on electrophysiology and behaviour show that treated animals exhibit better light-evoked behaviours and the restoration of unexpectedly complicated light-evoked retinal ganglion cell responses	[188]
Mice	Pluripotent stem cell-derived cone precursors	Human	Pde6brd1 mice as a model of retinal degeneration	Subretinal transplant	Behavioral tests Histology Immunohistochemistry Electrophysiology	Cone precursors produced from pluripotent stem cells have the ability to trigger light responsiveness even at advanced stages of degeneration	[189]

## Results

### Anatomy of the eye

The eyes are one of the most significant and complicated sensory organs; they serve as a gateway for external images, which are transmitted to the brain as impulses via the optic nerve. This mechanism helps to maintain a connection between the body and its surroundings. The eye is broadly divided into two segments: anterior and posterior. The anterior section includes the cornea, conjunctiva, aqueous humor, iris, ciliary body, and crystalline lens. These cover roughly one-third of the front of the eye. The remaining section, known as the posterior segment, includes the sclera, choroid, Bruch’s membrane, retinal pigment epithelium (RPE), neural retina, and vitreous humor [190]. A full discussion of the anatomy of the eye is provided here.

### Anterior segment

#### Cornea

The cornea is thin, transparent, smooth, avascular, densely innervated, and sensitive tissue. The cornea is one with the sclera and the conjunctiva. The limbus refers to the cornea’s border where it meets the sclera. The cornea is made up of six separate layers: corneal epithelium, Bowman’s layer, stroma, Dua’s layer, Descemet’s membrane, and endothelium [191]. Corneal epithelium consists of five to six layers of stratified and squamous non-keratinized epithelial cells. The cornea’s epithelial layers are made up of two to three layers of superficial and wing cells, as well as one layer of basal cells [190, 192]. The corneal epithelium is constantly shed and replaced, while the stroma, which makes up roughly 90% of the cornea and is primarily composed of highly organized collagen, is both tough and transparent. The corneal endothelium, a single-celled layer of epithelial cells, is responsible for maintaining deturgescence [193].

#### Conjunctiva

The conjunctiva is a thin, highly vascularized, semi-transparent membrane that secretes mucus. It constitutes the inner lining of the upper and lower eyelids. It reflects onto the eye as a thin translucent tissue on the sclera that continues to the corneal limbus [192].

### Aqueous humor

Aqueous humour is a clear, slightly alkaline ocular fluid produced by epithelial cells in the ciliary body. Aqueous humor generated and secreted in the posterior eye segment travels via the pupil to the anterior chamber. It drains into the venous bloodstream via the trabecular meshwork and Schlemm’s canal. Aqueous humor provides nutrients and some oxygen to the ocular avascular tissue, specifically the cornea and lens. It removes garbage, macrophages, blood, and other material from the posterior cornea and anterior lens [194].

### Iris–ciliary body

The iris is found in the posterior portion of the cornea and appears as a root of the ciliary body. The iris creates a small circular opening or aperture in front of the lens, known as the pupil, which serves to regulate the quantity of light that passes through to the retina. Each ciliary body contains a ciliary process, which has a fibro-vascular core that seems to be continuous with the ciliary body’s stroma [190].

### Lens

The lens membrane, commonly known as the capsule, is made up of four separate parts: the capsule, cortex, nucleus, and epithelium. The lens is transparent, avascular, non-innervated, and biconvex. It is supported by the zonular fibers of the ciliary body and is situated behind the pupil and iris. The anterior lens is covered with aqueous humour, while the posterior is covered with vitreous humor [195].

### Posterior segment

#### Sclera

The sclera is a tough, avascular, elastic tissue that runs parallel to the cornea beneath the conjunctiva. The lamina cribrosa is a densely woven network of fibrous tissue through which the optic nerve exits posteriorly [196].

#### Choroid

The inner retinal pigmented epithelium and the peripheral sclera are separated by the choroid. It is a highly innervated and vascularised tissue that contains melanocytes and extracellular fluid that resembles mucus. From outer to inner, the choroid is made up of three separate components: Bruch’s membrane, the vascular layer, and the suprachoroid. The interface between the inner choroid and outer sclera is formed by the six to ten layers that make up the suprachoroid. Three separate vessel layers with progressively shrinking capillary and luminal sizes make up the vascular layer. Above the RPE, the final and deepest layer of the choroid is called Bruch’s membrane. Another name for it is the lamina vitrea [190].

#### Retina

The neurosensory part of the eye is the retina. It is a transparent, thin, and delicate tissue that is made from neuroectoderm. These sensory neurones form the starting point of the visual pathway. The nine layers that comprise the neural retina (neuroretina) are the outer and inner segments of photoreceptors (rods and cones), the external limiting membrane, the outer nuclear layer, the outer plexiform layer, the inner nuclear layer, the inner plexiform layer, the ganglion cell layer, the nerve fibre layer, and the internal limiting membrane. Prior to starting signal transduction in the rods and cones, light must pass through these several layers [197].

## Bruch’s membrane

The choriocapillaries and the retinal pigment epithelium (RPE) are separated by Bruch’s membrane. The choriocapillaris’ basement membrane, the RPE’s basement membrane, an exterior collagenous layer, a middle elastic layer, and an inner collagenous layer make up this elastic membrane’s five layers. Elastin, several collagen types (types I–V, IX, XI, and XII), and a number of sticky glycoproteins, such as fibronectin and laminin, which aid in anchoring cells to Bruch’s membrane, make up the complex makeup of Bruch’s membrane [190].

## Retinal pigment epithelium

The retinal pigment epithelium (RPE), a monolayer of cuboidal cells with many melanosomes that give the cells their pigmented colour, is located beneath the photoreceptors. The choriocapillaries and the outer retina’s photoreceptors are separated by the approximately 3.5 million RPE cells that make up each eye’s continuous epithelial monolayer, which is held together by junctional complexes. This creates the outer blood–retina barrier, a selective barrier between the outer retina and its choroidal blood supply [197]. By aiding in the diffusion of nutrients from the choroid and the elimination of waste or worn-out photoreceptor segments, the RPE cells support the outer neurosensory retina that sits on top of it. Despite not dividing, these cells may multiply in unhealthy settings. In addition to secreting a lot of growth factors (vascular endothelial growth factor, ciliary neurotropic factor, and platelet-derived growth factor), it protects the inner ocular tissues [198]. This monolayer secretes immunomodulatory cytokines to preserve ocular immunity and shield against oxidative damage. A number of enzymes, including glutathione, catalase, superoxide dismutase, and melanin pigment, are produced by RPE cells. RPE is essential for the survival and sustenance of choriocapillaries as well as the operation of photoreceptors. As a result, its existence is necessary to preserve the visual function [199].

## Neural retina

### Photoreceptor layer

A single pallisading layer of photoreceptors is formed by the tightly packed stacking of rods and cones. The only area of the neuroretina that is light-sensitive and where phototransduction occurs is this thin, subcellular layer [200].

### External limiting membrane

The external membrane that limits Junctional complexes between neighbouring Müller cells and between Müller and photoreceptor cells forms this membrane, which is not a real membrane. The potential gap between the exterior limiting membrane and the outer blood–retina barrier is known as the subretinal space [201].

### Outer nuclear layer

The photoreceptor cells’ nuclei are found in the outer nuclear layer, which is thickest in the foveolar region. Rods are more common beyond the foveola throughout the rest of the retina, while only cones are found in the foveola. The “rod ring,” which is located around 4.5 mm from the foveola, has the highest rod density [200].

### Outer plexiform layer

The bipolar and horizontal cells of the inner nuclear layers link with the photoreceptor cells of the outer nuclear layer in the outer plexiform layer (OPL). The axons of the photoreceptor, bipolar, and horizontal cells, as well as their synaptic connections, make up the OPL. The photoreceptor cells’ axons create a specialised structure in the central retina called Henle’s fiber layer and carry the photosignal to the OPL [202].

### Inner nuclear layer

The nuclei of at least five distinct cell types are found in this layer: the longitudinal, bipolar, amacrine, interplexiform, and Müller cells. The amacrine cells face the IPL, while the horizontal cells are situated along the outer edge of the inner nuclear layer facing the OPL. The bipolar, interplexiform, and Müller cell nuclei occupy intermediate positions [203].

### Inner plexiform layer

The second processing layer of the retina, the Inner Plexiform Layer(IPL), is made up of networks of ganglion, amacrine, and bipolar cells [202].

Ganglion cell layer: About 1.2 million ganglion cells and several additional cell types, such as “displaced” amacrine cells, astrocytes, endothelial cells, and pericytes, are found in this layer. With eight to ten rows of nuclei (60–80 µm) in the perifoveal macula, the ganglion cell layer is thickest there. Outside the macula, it thins to a single row (10–20 µm), and it is not present in the foveola itself [204].

### Nerve fiber layer

Ganglionic axons move through the nerve fiber layer in the direction of the optic nerve head. All of the retinal ganglion axon fibers on the optic disc converge to form a thicker layer of nerve fibers, which is thin and hard to see at the far periphery. The internal limiting membrane and Müller cell cellular processes divide the axons into little bundles, while astrocytes accompany them in the nerve fibre layer [201].

### Inner limiting membrane

The inner limiting membrane is formed by the enlargement and flattening of the Müller cell’s innermost processes on the vitreal side. The retina is susceptible to vitreoretinal tension because vitreous collagen fibrils intrude into this membrane [205].

## Vitreous humor

Vitreous humor is a transparent, thick, gel-like fluid that covers the space between the lens and retina and helps to maintain the globe’s structure. The vitreous body and neural retina are separated from one another by an inner limiting membrane. The vitreous is loosely attached at the optic nerve and posterior macula, but firmly attached to anterior retinal layers at the ora serrata, which is present at the posterior segment of the iris-ciliary body [190]. Following a quick overview of the anatomy of the eye, we will now discuss the most common ocular disorders that affect various components of the eye.

## Ocular affections

Ocular surface affections, primarily affecting the cornea, such as limbal stem cell deficiency, corneal ulcers and burns, keratopathy, and keratitis, are the principal disorders that significantly impair vision or result in vision loss. Furthermore, disorders of the eye’s retina lead to the degeneration of neural and photoreceptor cells, which ultimately results in blindness. Also, retinal degenerative diseases such as retinitis pigmentosa, glaucoma, diabetic macular edema, and macular degeneration. Along with other ocular disorders such as uveitis.

### Corneal affections

The cornea is the outermost layer of the eye, and eyesight depends on it being transparent. Vision loss can arise from corneal diseases as limbal stem cell deficiency, corneal ulcers, endothelial dystrophy, bullous keratopathy, and keratoconus. Additionally, corneal damage from chemical burns and radiant energy from heat, electricity, and UV radiation can seriously impair vision or even cause blindness. The most important diseases affecting the corneal tissue are discussed below. Corneal therapies are obtained from limbal stem cells (LSCs) and mesenchymal stem cells (MSCs). Because of their accessible position and special ability to regenerate, LSCs are a great option for cell treatments to treat corneal damage. According to existing literature, the LSCS is the most prominent cell for corneal affections.

### Limbal stem cell deficiency

Limbic stem cell deficiency (LSCD) is brought on by a significant decrease and/or malfunction of limbal epithelial stem cells, which are in charge of the ongoing regeneration of the corneal epithelium. Loss of corneal clarity and vision impairment are the results of the conjunctival epithelium migrating across the limbus in LSCD. It is a painful and potentially blinding condition. An essential barrier that keeps conjunctival tissue from invading the cornea is provided by healthy, functional limbal epithelial cells (LEC). Since persistent inflammation damages the remaining stem cells and their function in addition to killing LEC, limb stem cell deficit usually gets worse over time [47, 206].

Numerous ocular surface conditions can cause LSCD, including immune-based conditions, congenital conditions, damage from chemicals, heat, or mechanical forces, infections, and the aftereffects of multiple eye procedures. This results in inadequate ocular surface renewal, which in turn causes chronic inflammation, neovascularization, secondary infections, and permanent epithelial abnormalities. Any of these may cause persistent pain, vision loss, and corneal opacity [206]. A summary of stem cells used in the treatment of LSCD in different animals is shown in Table 1.

### Corneal ulcers and burns

Chemical corneal damage is a serious ophthalmological emergency that carries a high risk of blindness. As a result, there has been an ocular injury that needs to be evaluated right away in order to recover the ocular surface and maintain corneal clarity. However, inflammation, angiogenesis, and conjunctivalization may result in loss of the eye limbus and central epithelium. Upon injury to the cornea, an unorganised, opaque matrix known as corneal scar tissue forms, reducing corneal transparency and may lead to blindness [47, 207]. Chemical eye injuries can aggravate uncomfortable problems such as corneal dryness, abrasions, ulcers, and perforations. Tear production can decline as a result of damage to the lacrimal, conjunctival, and eyelid glands. Extensive sub-conjunctival inflammation, mucus insufficiency, and sub-conjunctival tissue fibrosis are all brought on by severe injury to the conjunctival cells [47].

Chemical burns may result in an immediate or long-term increase in intraocular pressure (IOP) because of collagen shrinkage, sclera and corneal contraction, and altered uveal blood flow. The increase could be followed by a recovery to normal IOP or hypotony (caused by injury to the ciliary body), and then, the IOP would keep rising. Alkalis can damage the ciliary body, lens, and trabecular meshwork in as little as 15 s after they enter the anterior chamber. Long after the initial alkali exposure, penetration continues to occur. An elevated IOP over time may result from chronic trabecular meshwork degradation and the build-up of inflammatory debris. Severe complications include glaucoma, iritis, and impaired visual acuity [47]. A summary of stem cells used in the treatment of corneal ulcers and burns in different animals is shown in Table 2.

### Bullous keratopathy

Endothelial decompensation of the cornea results in bullous keratopathy (BK), the final clinicopathological manifestation that causes irreversible loss of corneal clarity. Stromal edema and epithelial bullae brought on by endothelial dysfunction are its defining features. These symptoms included sensations of a foreign body, pain, redness, reduced vision, photophobia, and halos surrounding lights. Significant vision loss and, in extreme situations, blindness are caused by the extensive corneal edema [208, 209]. Bullous keratopathy can be caused by a number of congenital and acquired diseases, such as endothelial dystrophy, iris-to-cornea persistent pupillary membranes, trauma, anterior uveitis, endotheliitis, glaucoma, endothelium-related toxic damage, age-related endothelial degeneration, and melting keratitis [210]. The level of stromal edema and opacification is usually not considerably reduced by topical use of hypertonic solutions and ointments, while it can reduce the extent of epithelial oedema and bullae development [211].

### Retinal affections

#### Retinal degenerative diseases (RDDs)

The term “retinal diseases” refers to a broad category of light-threatening conditions that include glaucoma, juvenile Stargardt’s macular dystrophy, diabetic retinopathy (DR), retinitis pigmentosa (RP), age-related macular degeneration (AMD), and many other comparable conditions. The degeneration of photoreceptors, loss of retinal integrity, and death of the specialized retinal cells are common features of retinal diseases, despite their diverse aetiologies and causes. These processes lead to visual impairment and eventually blindness [212, 213].

##### Age-related macular degeneration (AMD)

The aetiology of AMD, a degenerative condition, is influenced by a number of genetic and environmental variables. There are two types of AMD in its advanced stage: wet AMD and dry AMD. Dry AMD is typified by the loss of photoreceptors due to the damaged RPE layer’s inability to phagocytose the outer segments of the photoreceptors, as well as the degradation of the basement membrane and retinal pigment epithelium (RPE) layer. A lysosomal protein called lipofuscin accumulates as a result of incomplete phagocytosis, interfering with the RPE layer’s ability to function normally. The accumulation of drusen, or cell debris, between Bruch’s membrane and the RPE layer leads to the membrane to detach, which accelerates the development of wet AMD [9, 212]. Photoreceptor degradation, thickening Bruch’s membrane, and an increase in subretinal drusen appearance are the hallmarks of dry AMD, whereas choroidal neovascularisation is the hallmark of wet AMD, which causes significant vision impairment [214]. A summary of stem cells used in the treatment of retinal degeneration (macular) in different animals is shown in Table 3.

##### Retinitis pigmentosa (RP)

The inheritance patterns of retinitis pigmentosa (RP), a genetic degenerative disorder, are autosomal recessive, autosomal dominant, or X-linked recessive. As the disease progresses to later stages, degeneration of cones results in loss of central and colour vision, while the disease’s earlier stages include the destruction of rod photoreceptors, which results in night vision loss and restricted peripheral vision. Gene mutations are typically linked to the degradation of photoreceptors in RP [9, 213]. Pigmentary retinopathy, optic nerve pallor, progressive photoreceptor degeneration and eventual death, and nyctalopia—all of which lead to significant vision loss—are the hallmarks of retinitis pigmentosa. Furthermore, macular edema, subcapsular cataracts, and inflammatory vitreous cells [215]. A summary of stem cells used in the treatment of retinitis pigmentosa in different animals is shown in Table 4.

### Glaucoma

With over 70 million cases globally, glaucoma is the most common neurological disease. It is characterized by a substantial increase in intraocular pressure (IOP), which causes gradual loss of retinal ganglion cells (RGC), degeneration of the optic nerve head, and ultimately vision loss. Glaucoma causes the progressive loss of retinal ganglion cells (RGC) and their axons, resulting in a gradual loss of visual field. RGC loss may or may not be associated with elevated IOP levels, which contribute to glaucoma pathogenesis [216]. The ciliary body secretes aqueous humor, which flows and drains dynamically to determine intraocular pressure. The two distinct channels for aqueous humor drainage are the trabecular meshwork and uveo scleral outflow pathways. Open-angle glaucoma and angle-closure glaucoma are two distinct types of glaucoma that can result from both trabecular meshwork failure and anatomic position abnormalities of the iris obstructing the trabecular meshwork [9, 212].

The blockage of trabecular meshwork resulting from an irregular iris anatomical placement causes angle-closure glaucoma, while its malfunction causes open-angle glaucoma. In addition, neuronal damage associated with the accumulation of extracellular glutamate, free radicals, and excitatory amino acids, as well as hypoxia, ischemic insult, loss of nutrients and energy, neuroinflammation, and a decrease in the transmission of neurotrophic factors, all contribute to the damage of the retinal germ cells in glaucoma. Anterograde and retrograde axonal transport is disrupted by glutamate-induced excitotoxicity, and RGCs die from axotomy-induced death [217].

Currently, the only proven treatments that slow down the evolution of the condition are medication-assisted intraocular pressure reduction and eye surgery. While these treatments can postpone the onset of the disease, they are unable to stop neurodegeneration in the long run, and vision impairment due to optic nerve damage and loss of RGC is permanent. A summary of stem cells used in the treatment of glaucoma in different animals is shown in Table 5.

### Diabetic retinopathy (DR)

As a result of several abnormal metabolic processes that produce an excess of reactive oxygen species (ROS) and persistent hyperglycemia, diabetic retinopathy is the most common microvascular complication of diabetes mellitus (DM). An early or non-proliferative stage of diabetic retina disease (NPDR) is characterized by loss of pericytes, endothelial cells, and neuronal cells in the retina. Proliferative stage of DR (PDR): As the illness advances to a more severe stage, abnormalities in the intraretinal vasculature and bleeding occur due to pro-angiogenic and inflammatory responses. Since PDR is brought on by neovascularization, standard treatment strategies involve anti-VEGF to inhibit uncontrolled angiogenesis [9, 213]. Retinal haemorrhage, tortuosity, micro-aneurysms, and lipid exudates are characteristics of nonproliferative DR, an early stage of the condition. The development of delicate aberrant vessels, on the other hand, is a hallmark of proliferative DR, an advanced stage. Underlying causes of vision loss in both levels of DR are diabetic macular oedema and blood–retinal barrier (BRB) breakdown. The build-up of fluid in the neural retina causes thickening and cystoid oedema in diabetic macular edema, while vascular leakage is linked to BRB [218, 219]. A summary of stem cells used in the treatment of diabetic retinopathy is shown in Table 6.

### Retinal ischemia (RI)

Retinal ischemia is associated with diseases such as diabetes, glaucoma, central/branch retinal artery/vein blockage, and potentially, age-related macular degeneration [220]. Reactive oxygen species (ROS) like H_2_O_2_ are created in high quantities following ischemia/reperfusion (I/R). These reactive oxygen species (ROS) destroy neighbouring cells and tissue [221]. Furthermore, neuronal overstimulation and undesirable depolarization result from the increased release of excitatory transmitters like glutamate from ischemia-affected neurones. As a result, neurones with a high glutamate receptor density are most vulnerable. This clarifies why neurones in the inner retina, such as retinal ganglion cells (RGC) and amacrine cells, are susceptible to I/R, as are their neuronal processes [222]. A summary of stem cells used in the treatment of retinal ischemia is shown in Table 7.

### Retinal detachment (RD)

Retinal detachment is one of the most prevalent disorders affecting vision that involves the separation of the neurosensory retina (including photoreceptors) from the underlying retinal pigment epithelium (RPE), which carries nutrients (including glucose) to the photoreceptors [223, 224]. The process of separation has the potential to reduce the amount of oxygen and nutrients that reach the outer segments of photoreceptors, creating a relatively hypoxic environment. This could further hinder the energy production needed to transport nutrients to the photoreceptors, ultimately decreasing their effectiveness. In addition to producing oxidative stress, hypoxia can also result in photoreceptor apoptosis [225]. Furthermore, photoreceptor loss is further facilitated by inflammatory cytokines secreted in retinal degeneration. While improvements in surgical care have led to a significant rise in the rate of anatomic reattachment, additional factors such as photoreceptor necrosis, autophagy, apoptosis, and retinal remodelling can also cause alterations in the structure and function of the retina [226]. A summary of stem cells used in the treatment of uveitis, retinal detachment, and optic neuropathies is shown in Table 8.

### Uveitis

The term “uveitis” describes inflammation of the choroid, retina, vitreous body, iris, or ciliary body. Eighty causes of uveitis have been reported in the literature; these fall into five broad categories: drug-induced, ophthalmologic entities, infectious diseases, systemic diseases, and unknown origin [227]. The treatment options for autoimmune uveitis include topical steroids, systemic glucocorticoids, biologics such as anti-tumor necrosis factor (TNF), and immunosuppressants like methotrexate. However, these treatments continue to have a high rate of side effects and nonresponse [228].

Autoimmune uveitis (AU), which affects pigmented vascular structures in the eye, is a leading cause of avoidable blindness. In AU, ocular antigens leaking from the eyes are absorbed by dendritic cells and presented to autoreactive T cells in lymph nodes, resulting in an abnormal, uncontrolled, and overexuberant T cell-mediated host immune response, while B cells also contribute to antigen presentation and subsequent T cell activation [229]. Furthermore, T helper 17 cells play an important role in autoimmune uveitis pathogenesis as uveitogenic effectors, and a higher proportion of T helper 17 cells in the ocular inflammatory infiltrate is associated with more severe uveitis. Glucocorticoids are still the most commonly used immunosuppressive treatment for AU patients, despite the presence of several side effects and resistance [230]. A summary of stem cells used in the treatment of uveitis, retinal detachment, and optic neuropathies is shown in Table 8.

### Optic neuropathies

One category of serious pathological diseases that can cause blindness or irreversible visual impairment is known as optic neuropathies. They may be noticeable in ischemia, tumor compression, inflammation, glaucoma, or trauma injury. A common disease following optic nerve (ON) injury is the mature RGC’s apoptosis, which occurs several days later. The injured axons are unable to repair and expand into the myelin-rich environment distant from the injury site [153]. The low intrinsic regenerative ability of the retina, the presence of myelin-associated inhibitors, the formation of scars at the injury site, and the absence of appropriate trophic support have all been implicated in regenerative failure. However, RGC regeneration can be partially enhanced by stimulating RGC growth and modifying the microenvironment [231, 232]. A summary of stem cells used in the treatment of uveitis, retinal detachment, and optic neuropathies is shown in Table 8. A summary of stem cells used in the treatment of other retinal degenerative diseases is shown in Table 9. Following a review of the numerous eye affections, we will discuss one of the most important treatments for these disorders, regenerative medicine via cell therapy using stem cells. Different types of stem cells, their origins, their mechanisms of action to promote corneal and retinal regeneration, and injection methods for delivering these cells to different areas of the eye based on the type and location of the injury will all be covered in this section.

### Cell therapy

Cell treatments are another promising therapeutic technique that involves employing stem-like precursor cells to induce differentiation of certain cell types that are damaged by a specific eye disease and then delivering or implanting the cells in the affected tissue area to improve vision. Stem cells are immature, undifferentiated cells. These cells have some properties. Firstly, they have the capacity for proliferative division and long-term multiplication [233, 234]. Secondly, they have the capacity to differentiate into specialized cells as osteocytes, adipocytes, and chondrocytes through external and internal stimuli. The genetic makeup of the cell regulates internal stimuli, while the external stimuli are controlled by molecules in the surroundings, physical contact with nearby cells, and chemical mediators released by neighboring cells [235]. Finally, the minimum criteria for identifying multipotent MSCs have been established by the International Society for Cellular Therapy (ISCT). These criteria include being plastic-adherent under standard culture conditions, positive for the expression of CD105, CD73, and CD90 surface markers, and negative for the expression of CD11b, CD14, CD19, CD34, CD45, CD79a, and HLA-DR surface markers [236, 237]. Because of their special qualities, stem cells are being researched as a potential treatment for a wide range of diseases [238].

#### Types and sources of stem cells used in ophthalmology

**Fig. 1:**
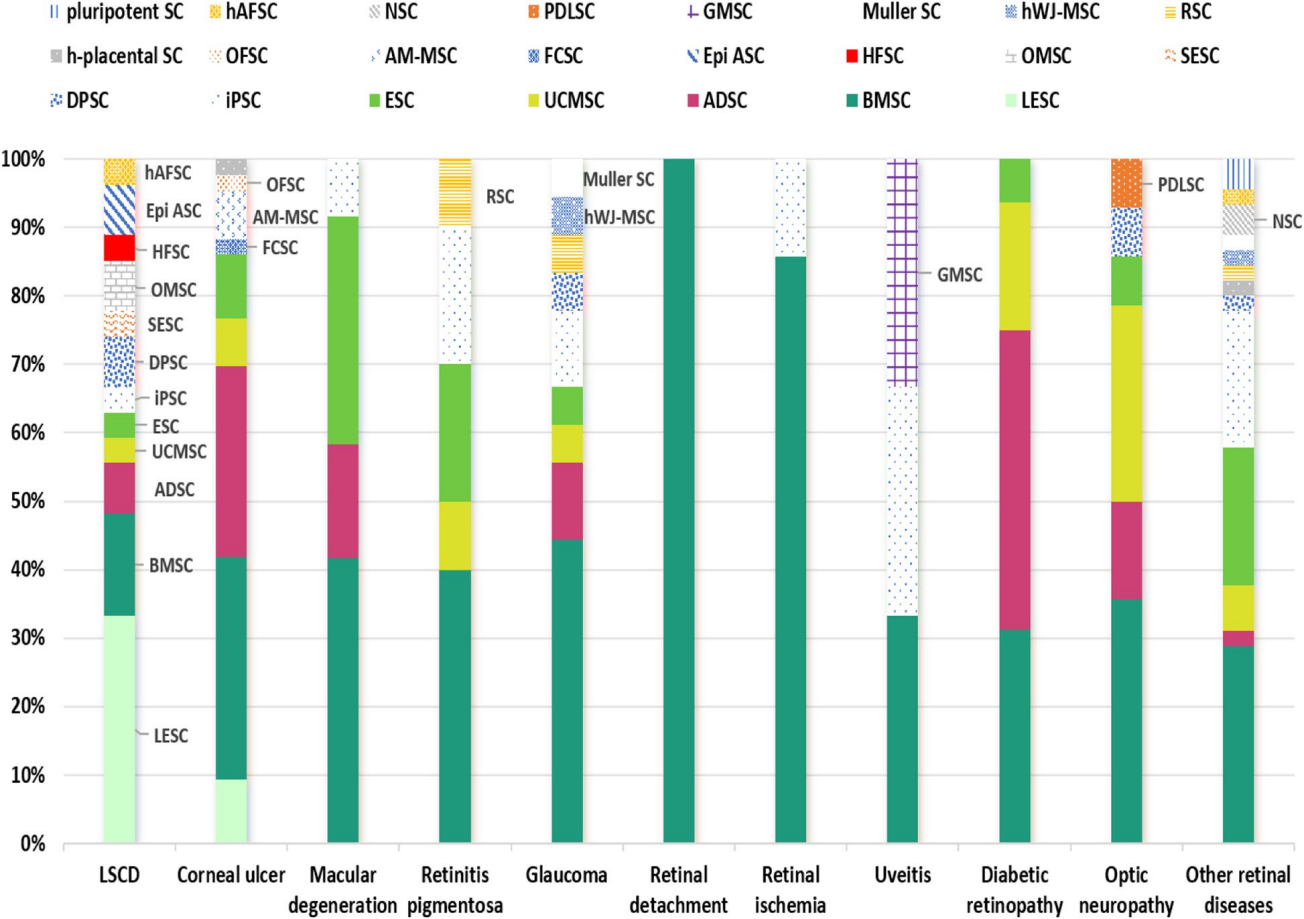
Different therapeutic types of stem cells used in ocular affections

Cell-based therapeutics are now being developed for anterior and posterior ocular diseases. The source of the stem-like cell population is determined by the target cell type. Corneal therapies are made up of limbal stem cells and mesenchymal stem cells (MSCs). The retina is the other ocular component being studied for cell treatments, with embryonic-like stem cells, induced pluripotent stem cells (iPSCs), and neural stem cells (NSCs) employed to generate RPE and retinal cell types [8]. The different therapeutic types of stem cells used in ocular affections are shown in Fig. 1.

### Adult stem cells

Mesenchymal stem cells are derived from adult tissues in vitro and can be found in a variety of organs, including the blood, blood vessels, skeletal muscles, skin, teeth, bone marrow, fat, and cartilage. The most often employed MSCs come from the bone marrow and fat. These cells are known as multipotent because they have the ability to differentiate into a wide range of specialized body cells. Recent research has looked into MSCs as a possible alternative source of stem cells for LSC transplant therapy for corneal damage.

#### Bone marrow mesenchymal stem cells (BMSCs)

The first source of stem cells to be studied for isolation was the bone marrow. Because BMSCs may be grown and cultured again, fewer procedures are needed, and there is a lower chance of infection. Although bone marrow aspiration from the posterior iliac crest is favored because it yields a larger concentration of nucleated cells, BMSCs can be separated from vertebral bodies, the humerus, femur, tibia, or calcaneus.

A comparison of MSCs produced from bone marrow, umbilical cord blood, and adipose tissue revealed that MSCs derived from bone marrow have the lowest proliferation capability and the shortest culture duration [239, 240].

#### Adipose-derived stem cells (ADSCs)

There are several benefits associated with using adipose tissue as a source of stem cells. The first is that a smooth, painless method can be used to obtain adipose-derived stem cells (ADSCs) in large quantities. Second, ADSCs can be taken under local anesthesia again and again [241, 242]. Third, compared to BMSCs, ADSCs have a longer lifespan, demonstrate more ability for proliferation and their availability, and are easier to separate by enzymes. When considering alternative stem cell sources, autologous stem cells are comparatively safe and are being utilized. Certain research indicates that ADSCs can be stimulated to develop into keratocytes, retinal progenitor cells, and phenotypes similar to corneal epithelial cells [243].

#### Embryonic stem cells (ESCs)

Embryonic stem cells (ESCs) are produced in vitro from blastocysts, which are the inner cell mass of embryos extracted during the first 3 to 5 days of early embryonic development and are useful as donor cells for retinal regeneration. The fact that these cells can differentiate into any type of body cell originating from the ectoderm, mesoderm, or endoderm makes them pluripotent. Moreover, it is feasible to eliminate these cells without harming the embryo. However, the use of human embryos raises ethical concerns, and immunological rejection following ESC transplantation may limit the use of ESC-based regeneration treatments [244, 245].

Among the tissues afflicted by retinal degenerative illnesses, the RPE is the most responsive to ESCs treatment because, in contrast to neuronal cell types, RPE cells do not need to make synaptic connections. Since neurones must establish synaptic connections with the right neurones in addition to the problem of cell integration, neural cell types are among the most difficult to build cell-based therapies for in the eye [8].

Another possible ESC-derived treatment for extremely severe types of retinal degeneration is photoreceptor cell therapy. Similar difficulties to RGC therapy arise with photoreceptor therapy, such as synapse development and cell integration [246]. Cataract formation and other anterior segment disorders are also treated with ESCs. The most popular treatment involves replacing the lens with a synthetic intraocular lens [247].

### Induced pluripotent stem cells (iPSCs)

Induced pluripotent stem cells (iPSCs), an alternative to ESC-based treatments for replacing cell types that have completely deteriorated in illnesses, are cell therapies created from iPSCs. The source population—iPSCs—can be extracted from the patient, which is one of the benefits of iPSC-derived cell therapies. Additionally, some of the ethical issues surrounding hESCs do not apply to iPSCs [8].

The first of the drawbacks of iPSC-derived cell therapies is that the lack of improvement in visual function may be due to the disease-causing mutations present in the patient’s cells, which would also be replicated in the iPSCs created from that patient. Getting iPSCs from healthy donor tissue—ideally from close family members—who do not have any known illnesses or co-morbidities would probably be a preferable option. The decreased ability of iPSCs to survive and integrate into the tissues of the eyes is another drawback of their use [6].

It has been discovered that induced pluripotent stem cells (iPSCs) share characteristics with embryonic stem cells. Four transcription factors—Oct-3/4, Sox2, c-Myc, and Klf4—can be added to somatic cells to create induced pluripotent stem cells [248]. ESC-like traits are also present in iPSCs, including telomerase activity, surface antigens, cell shape, proliferative capacity, gene expressions, and the epigenetic state of genes peculiar to pluripotent cells. iPSCs possess great therapeutic promise for regenerative therapy since they can develop into cell types of the three germ layers [249].

By retrovirally transducing four transcription factors—Oct4, Sox2, KLF4, and c-Myc—from mouse dermal fibroblasts, iPSCs were produced to treat rabbit LSCD [24], rat macular degeneration [80, 166], and retinal degeneration in mice [177, 182]. By infecting human patient-specific keratinocytes with four distinct non-integrating Sendai viruses, which were individually engineered to promote the expression of one of four transcription factors, iPSCs were produced to treat retinitis pigmentosa in mice [93]. In order to treat glaucoma in rats, the adult mouse limbal progenitors were cultured in the presence of ES cell conditioned media, which reprogrammed them non-cell autonomously to pluripotency [108]. Mice with retinal ischaemia were treated by engrafting and homeomizing human cord blood-induced pluripotent stem cells (CB-iPSCs) into damaged retinal capillaries [135]. Peripheral blood was extracted from a male donor who was 40 years of age at the time of donation and had achromatopsia as a result of a homozygous deletion in the CNGB3 gene in order to reprogram peripheral blood mesenchymal cells (PBMCs) into iPSCs for the treatment of retinal degeneration in mice [188]. Yuan et al. [139] used induced pluripotent stem cells derived from human urine cells (UCs) to treat uveitis in mice. Plasmids containing the four transcription factors were electroporated into UCs. Nrl-eGFP mice were used to create iPSCs in order to treat retinal degeneration in mice [180, 187]. Mouse iPSC-TM cells were generated from 1Nagy/J transgenic mice in order to treat glaucoma in mice [112].

### Umbilical cord-derived MSCs (UC-MSCs)

The umbilical cord is a well-known and reasonably priced source of MSCs. The primary benefits of this source are its enormous quantity, simplicity in acquisition, and accessibility to donors [250]. Large amounts of UC-MSCs can be collected, cryopreserved, and kept for future use. They also combine the prenatal and postnatal properties of MSCs uniquely. Because UC-MSCs are immature compared to adult stem cells, they are less able to withstand HLA mismatches and produce a weaker immune response in an allogeneic recipient [251].

### Amniotic membrane-derived MSCs (AM-MSCs)

AM-MSCs have cheap processing costs and are readily isolated in vitro from amniotic membranes. Because they only express low quantities of major histocompatibility complex class I antigens and lack major histocompatibility complex-II antigens, they can survive in recipients that are mismatched allogenically [51]. The ophthalmological field has extensively employed the amniotic membrane for wound healing, re-epithelialization, and anti-inflammatory effects. It has anti-scarring, analgesic, and bacteriostatic qualities [252].

### Neural stem cells (NSCs)

Neural stem cells (NSCs) that are capable of proliferating and differentiating further are found in the adult mammalian central nervous system. Retinal cell transplantation may benefit from the partially differentiated progenitor populations that have been identified from the brain or eye. Furthermore, we discovered that the stimulation of NSCs to differentiate into opsin-positive cells was significantly aided by the combination of retinoic acid and transforming growth factor beta type III. Mature photoreceptor cells are often produced by the cells incorporated into the outer retinal layer based on the cellular morphology, expression of photoreceptor markers, and functional recovery [6, 253]. The neuroprotective effect of NSC-derived cell therapies on photoreceptors has been the primary focus of research. Transplanted NSCs, however, have a neuroprotective role rather than acting as a cell replacement therapy because they do not develop into photoreceptor cell types [6].

### Dental pulp-derived MSCs (DP-MSCs)

MSCs from deciduous teeth can be separated and cryopreserved for medical use. They isolated using enzymatic digestion from third molars [254].

### Limbal stem cells (LSCs)

Limbal stem cells are a population of stem cells that can replace and regenerate corneal cells following corneal injury. They are found in the limbus’s palisade of the Vogt area. Because of their special ability to regenerate and their accessibility, limbal stem cells (LSCs) are an excellent option for cell therapies aimed at treating corneal damage. This stem cell population can be lost because of corneal burns and injuries to the limbal region. This can cause conjunctival invasion of the cornea and neovascularization, which can reduce corneal transparency and result in vision loss. As such, the treatment of corneal diseases leading to opacification is currently being investigated, including repopulation of the limbal region with LSC therapy [70]. Because LSCs are so scarce, current research is also investigating the use of other types of stem cells as a substitute [8].

Mesenchymal stem cells (MSCs) are thought to be promising candidates for treatment of several corneal affections and retinal degenerative disorders. In this section, we cover some important characteristics of MSCs that help in regeneration and repair, including paracrine substances produced by the cells, differentiation of the stem cells, exosomes, and mitochondrial translocation into host cells.

### MSCS’s roles and mechanisms in corneal wound healing

MSCs help tissue wounds repair. MSCs may migrate to tissue injury sites. MSC roles in corneal wound recovery are driven by two mechanisms: trans differentiation and paracrine action.

#### MSC mobilization and homing

Stem cell mobilization, migration, and colonization are induced by injury and inflammation. Certain chemoattractants released by corneal damage cause the bone marrow to discharge endogenous MSCs into the peripheral circulation. As a result, circulating MSCs proliferate and move to the nearby corneal injury but not to the healthy cornea [255–257]. MSC recruitment and mobilization to ocular injury sites are regulated by the chemokines in the cauterized cornea. In addition, the presence of chemokines, chemokine receptors, intracellular signals, adhesion molecules, and proteases can cause systemically injected MSCs to move towards wounded or inflamed ocular tissues [257].

#### Paracrine action and neuroprotective factors

MSCs release soluble molecules that help to promote tissue wound healing. Through paracrine action, the soluble factors generated by MSCs have anti-inflammatory and anti-angiogenic properties. The anti-angiogenic factor thrombospondin-1 (TSP-1) and the anti-inflammatory cytokines IL-10, TGF-1, and IL-6 are upregulated, and the pro-inflammatory factors IL-2, interferon-γ (IFN-γ), macrophage inflammatory protein-1α, and vascular endothelial growth factor (VEGF) are downregulated in injured corneas following MSC transplantation [58].

IL-6 functions in the eyes in two ways. While IL-6 shields the ocular tissues against unwanted infections, it can also exacerbate inflammation or cause unintended neovascularisation, which can harm and destroy the eyes’ delicate components. The conjunctiva, cornea, iris, retina, and orbit all have significant roles in ocular inflammation and angiogenesis that are influenced by IL-6 [258].

#### Differentiation into corneal cells

Corneal keratocytes are quiescent, flat cells with a dendritic structure. Keratocytes may be activated by injury, and keratocan and lumican production are down-regulated during wound healing [19]. Keratocytes grown in vitro under serum-containing growth conditions take on the characteristics of activated cells. MSCs are an excellent choice for restoring damaged corneal endothelium. Corneal endothelial cells are primarily responsible for maintaining the cornea’s transparency and nourishing it by drawing water out of it. Cells cannot be restored once they are lost or destroyed. The differentiation of MSCs into cornea epithelial-like cells can be accomplished in vitro by culturing the MSCs in a conditioned medium, a medium supplemented with signalling molecules only, or by co-culturing the MSCs with the signalling cells via a 3D scaffold system or a cell culture insert [259]. The expression of corneal epithelial cell surface markers CK3, CK12, E-Cadherin, and PAX6 on the cell surface can be used to determine whether the differentiation of ADSC has been successful. Other corneal cell markers, such as ZO-1, Na + ATPase, AQP1, and N-cadherin, are used to identify differentiation towards corneal endothelial cells, while the expression of p63 and ABCG2 is more suggestive of limbal epithelial stem cells [260, 261].

### Mechanism of MSCs for the treatment of retinal disorders

In this section, we discuss some significant properties of MSCs, such as the paracrine factors secreted by the cells, the exosomes, and mitochondrial transfer into host cells that facilitate the repair and regeneration of the retinal layer.

### Paracrine action and neuroprotective factors

Bone marrow mesenchymal stem cells (BMSCs) secrete a variety of neurotrophic factors (NTFs), including brain-derived neurotrophic factor (BDNF), ciliary neurotrophic factor (CNTF), glial cell-derived neurotrophic factor (GDNF), platelet-derived growth factor (PDGF), nerve growth factor (NGF), neurotrophin-3, 4/5 (NT-3, 4/5), basic fibroblast growth factor (FGF2), and erythropoietin (EPO). Neural cell survival, differentiation, axonal extension, neural cell attachment, and inhibition of apoptosis are all improved by the neurotrophic factors released by BMSCs when they bind to their appropriate receptors on the recipient cells [262, 263]. A variety of NTFs, including hepatocyte growth factor (HGF), CNTF, IGF, FGF2, epidermal growth factor (EGF), VEGF, NGF, BDNF, GDNF, NT3, and PDGF, are secreted by adipose-derived mesenchymal stem cells (ADSCs), just similar to BMSCs [264].

#### MSCs dampen inflammatory responses

Ocular immune privilege is the capacity of the eye to prevent intraocular inflammation, hence preserving visual acuity and shielding the visual elements from harm [265]. The blood–retinal barrier (BRB), which effectively isolates the eye from the immune system, the ocular microenvironment’s local inhibition of innate and adaptive immune responses, and ocular-specific mechanisms that activate immunosuppressive regulatory T cells systemically all contribute to the maintenance of this intricate phenomenon [266].

The process by which MSCs suppress the immune system includes cell-to-cell interaction that represses the maturation and activity of B cells, T cells, natural killer cells, neutrophils, and macrophages [267]. Immunomodulatory cytokines, including nitric oxide (NO), indole amine 2,3-dioxygenase (IDO), prostaglandin E2 (PGE2), thrombospondin type 1 (TSP1), interleukins 6, 10 (IL6, IL10), TGFβ1, and HGF, are secreted by MSCs and trigger the functional regulation of these immune cells and anti-inflammatory responses [268].

Exosomes produced from MSCs also contribute to the modulation of inflammation by encouraging the transition of macrophages from the pro-inflammatory M1 phenotype to the anti-inflammatory M2 phenotype, activating Treg cells, suppressing B lymphocytes, and preventing neutrophil mobilization [269].

### MSCs modulate angiogenesis

Pathological angiogenesis is a hallmark of retinal diseases such as AMD, diabetic retinopathy, uveitis, and retinal vasculitis that result in irreversible visual loss [270]. The release of paracrine anti-inflammatory and anti-angiogenic substances was more important for the effective restoration of damaged ocular tissues by MSCs than was their development into ocular cells [57, 60]. It has been discovered that MSCs have either a pro- or anti-angiogenic effect depending on the tissue environment into which they are transplanted [271].

### MSCs replace pericytes

Pericytes are a diverse group of blood vessel cells that protect and stabilize the retinal microvasculature. They are embedded in the basement membrane of the vasculature. One of the main causes of diabetic retinopathy (DR) pathogenesis is vasoregression, which is brought on by the loss of pericytes brought on by hyperglycemia [272]. Due to their morphological and functional similarities with pericytes, MSCs have been proposed in several studies as a potential replacement for pericytes. As a result, MSCs may offer therapeutic benefits in the early stages of DR [273]. MSCs and pericytes display similar cell surface markers as adipose tissue-derived stem cells, which are extracted from the adipose tissue’s stromal vascular fraction. ADSCs were discovered in the adipose tissue at perivascular sites, where they expressed genes typically characteristic of pericytes, stabilized the vasculature, and prevented apoptosis of endothelial cells [274].

### MSCs donate mitochondria

According to several studies, MSCs repair damaged cells by transferring healthy, functional mitochondria to them through gap junctions, exosomes, and tunnelling nanotubes [275]. Numerous retinal diseases, including AMD, DR, and glaucoma, are associated with mitochondrial dysfunction; hence, mitochondrial transfer therapy may be very effective in treating these conditions [276].

### MSC-derived extracellular vesicles (MSC-EVs)

MSC-EVs, also known as exosomes, are nanoscale microvesicles (40–100 nm) that contain functional components like lipids, proteins, and miRNAs and have the potential to have significant therapeutic impacts. MSC-derived exosomes can counteract the drawbacks of cell-based therapy, such as immunogenic and carcinogenic hazards, transplant failure, and increased potential for creating artificial, function-specific exosomes that will promote neuroprotection and retinal regeneration [277].

### Differentiation into retinal cells

Autologous MSC transplantation may be a potential technique for cell replacement therapy in retinal disorders. It has been discovered that BMSCs, ADSCs, DPSCs, and UMSCs can effectively develop into diverse retinal lineage cells in vitro and express genes associated with retinal cells [9].

#### Advantages of cell therapy in the eye

The transplanting process requires a very small amount of cells. The capacity to transplant cells into specific areas of the eye globe while under direct vision. Ocular barriers prevent transplanted cells from migrating outside of the eye’s globe [278]. Allogeneic transplantation of ocular tissue without long-term immunosuppression is known to provide partial immune privilege [279].

#### Methods of delivery of MSCs

Multiple methods exist for delivering MSCs to the ocular surface. Because MSCs have adhesion molecules like CD94d on their surface and are between 20 and 30 μm in diameter, systemic administration of MSCs may cause the cells to become trapped in the lungs, limiting their therapeutic potential. This issue can be resolved by localising the administration of MSCs to the ocular surface [280]. There are several ways to administer the cells to the posterior segment ocular tissues, including systemic administration, intravitreal injections, and periocular injections.

### Local administration

Topical administration is a simple and noninvasive technique for applying stem cells directly to the injured cornea. Because MSCs may stick to the surface of the eye, they can be applied topically. However, because of a high rate of washing, the corneal epithelium is not extremely permeable and has a short retention period [281].

Scaffold-based delivery, the difficulties associated with topical distribution, may be addressed by employing carriers to move cells to the intended location. Amniotic membranes are thought to be the best option for delivering cells to the ocular surface because of their extensive history of use in ophthalmology. Amniotic membrane functions as a constitutive basement membrane and could improve MSCs’ anti-inflammatory qualities. To help MSCs migrate to the ocular surface, a temperature-responsive scaffold was created [282].

Subconjunctival injections, high-dose treatments, can be administered continuously using subconjunctival injections, which are administered into the area between the conjunctiva and Tenon’s capsule. The term “periocular administration” describes the injection of a medication close to the ocular organ, such as the sub-conjunctival, sub-tenon, or parabulbar areas, in order for the medication to pass through the choroid, RPE, and sclera and enter the vitreous cavity. However, there are drawbacks to periocular medication administration as well, such as increased intraocular pressure, cataract formation, strabismus, and corneal decompensation [283].

Intrastromal injections, the intended site would receive MSCs directly from intrastromal injections. This technique has been applied to animal models of alkali chemical burns and congenital corneal disease in order to provide UC-MSCs [284].

Intracameral injection, an intracameral injection, is one way that MSCs can be given to the eye. The quick washout of cells through the trabecular meshwork is the primary issue with this method [51].

Intravitreal injection, in order to attain therapeutic concentrations at the posterior segment, intravitreal injections, and vitreal implants, has been studied; however, both of these administration options are highly risky and invasive. Regular intravitreal injection is linked to short-term side effects such as endophthalmitis, retinal detachment, intravitreal hemorrhage, and an elevated risk of developing cataracts [280]. Following the intravitreal injection of adipose stem cells into three patients in the USA, serious bilateral vision loss occurred. Visual loss was linked to ocular hypertension, hemorrhagic retinopathy, vitreous hemorrhage, traction and rhegmatogenous retinal detachment, and lens displacement. Following a year, the patients’ visual acuity ranged from 20/200 to no light perception [285].

Subretinal injection offers a novel treatment approach for vitreoretinal illnesses, particularly when gene therapy and/or cell therapy are used. This is because subretinal injection has a more direct effect on the targeting cells in the subretinal area than intravitreal injection [286].

Systemic administration, after intravenous injections, of MSCs was administered to damaged tissues. Nevertheless, after systemic treatment, less than 1% of cells will reach the target area. Therapeutic agents have also been delivered to the posterior segment of the eye through systemic administration; however, due to the inner and outer blood–retinal barriers that isolate the retina and vitreous humor from the systemic circulation, this method of administration necessitates high dosages [287].

### Current cell treatment failures and the ways to enhance transplantation

The therapeutic potential of cell treatment is influenced by a variety of factors, including the patient’s age, mechanical, and pathological circumstances. Mechanical stress during the transplantation procedure, a harsh microenvironment caused by the activation of inflammation-related factors, oxygen and nutrient starvation due to poorly vascularized environments at the site of implantation, and a lack of delivery protocol optimization may all have an impact on transplanted cell survival [288].

To maintain or augment transplanted cells’ resistance to hypoxic stress, donor cells could be preconditioned or modified before implantation. Tissue engineering could improve the longevity of transplanted cells by using appropriate biomaterials as carriers, such as a biologic-derived ECM scaffold [288].

#### Important factors for a successful stem cell transplantation

The following crucial actions are necessary for a successful transplant in cell therapies: (1) cell origin (embryonic stem cells, adult MSCs from various sources, iPSC); (2) injection route, as little invasive as feasible; and (3) local microenvironment, as exemplified by (a) extracellular matrix signals and (b) an appropriate growth factor combination [289].

(1) Choosing the cells. The target cell type determines where the stem-like cell population comes from. Both mesenchymal and limbal stem cells are used in corneal treatments. Neural stem cells, induced pluripotent stem cells, and embryonic-like stem cells are used to produce RPE and retinal cell types in the retina, the other ocular component under investigation for cell therapies [278].

(2) Mode of administration, the route of administration has to be as less intrusive as possible. Chemokines and their receptors control the migratory and organ-specific homing of stem cells. It has been documented that both adult and embryonic stem cells express CXCR4. Many organs express stromal cell-derived factor-1 (SDF-1), a particular CXCR4 ligand that is increased in response to ischaemia or injury. MSCs have the ability to target and integrate into the neuroretinal layer. Only the damaged retinal tissue showed migration of the transplanted stem cells; the normal retina did not [290].

(3) Cell delivery scaffolds and biomaterials for enhancing the local microenvironment, cells injected in a suspension frequently do not survive and do not develop into a completely differentiated phenotype in regeneration models. (a) Signals from the extracellular matrix. The integrity of Bruch’s membrane, the underlying substrate, is crucial for the survival of cells transplanted into the subretinal region during retinal restoration. The benefits of employing biodegradable scaffolds, such poly(L-lactic acid) and poly (D, L-lactic-co-glycolic acid), which may enhance retinal photoreceptor cell (RPC) survival and development [278].

(b) Retinal regeneration growth factors: numerous studies have been conducted on several growth factors in relation to retinal functional reconstruction. Neurotrophin-4, bFGF, ciliary neurotrophic factor, and BDNF can help RPCs recover from damage and stop retinal photoreceptor cells from degenerating [291].

### Animals used for ocular research

Mice: Since mice’s eyes resemble those of humans, they are used extensively in experimental animal studies. The most commonly used strain in LSCD research is the C57BL6 mice. A key advantage of using mice for experimental research is the possibility of genetically altered mouse breeds [292]. This allows researchers to reproduce essential aspects of human genetic disorders and investigate the involvement of particular genes and molecular pathways involved in the disease process. The growing use of mice in animal studies can also be attributed to advances in biochemical techniques that require smaller tissue samples, as well as the availability of a diverse range of analytical kits and reagents for studying mouse models. However, the tiny size of the mouse eye necessitates a larger number of mice per experiment than comparable tests employing rat eyes [293].

Rat: The Sprague Dawley rat is the strain most commonly used. Rats are very popular in research because they are inexpensive and simple to produce, which makes them ideal for a variety of wound healing studies. Rats can be administered inhalational anaesthesia, which offers a greater degree of control over the anesthetic process and lowers mortality compared to mice and rabbits, which need injectable anaesthesia [294].

Rabbit: Rabbits’ eyes and human eyes share anatomical similarities, which have led to their widespread use as models for a variety of ocular illnesses. The most widely utilised variety of rabbit is the New Zealand White rabbit (Oryctolagus cuniculus). Because rabbit corneas are similar in size to human corneas, diagnostic techniques designed for human usage, such as in vivo confocal microscopy and anterior segment optical coherence tomography, can be easily used to research rabbit corneas [294]. Drawbacks of using rabbit models include the lower availability of genetically modified strains, the higher expense of acquisition and upkeep compared to rats and mice, and the restricted supply of polyclonal antibodies against target proteins. Furthermore, it has been noted that rabbits’ respiratory centres are more sensitive to anaesthetics, which raises the possibility of respiratory suppression during anaesthesia administration [293].

Goat: Like other large animals, this model has a number of drawbacks, such as the absence of inbred strains, high maintenance and procurement costs, the challenge of managing larger animals, and the scarcity of a variety of analytical reagents for thorough assessments that are easier to obtain in murine models [295].

**Fig. 2:**
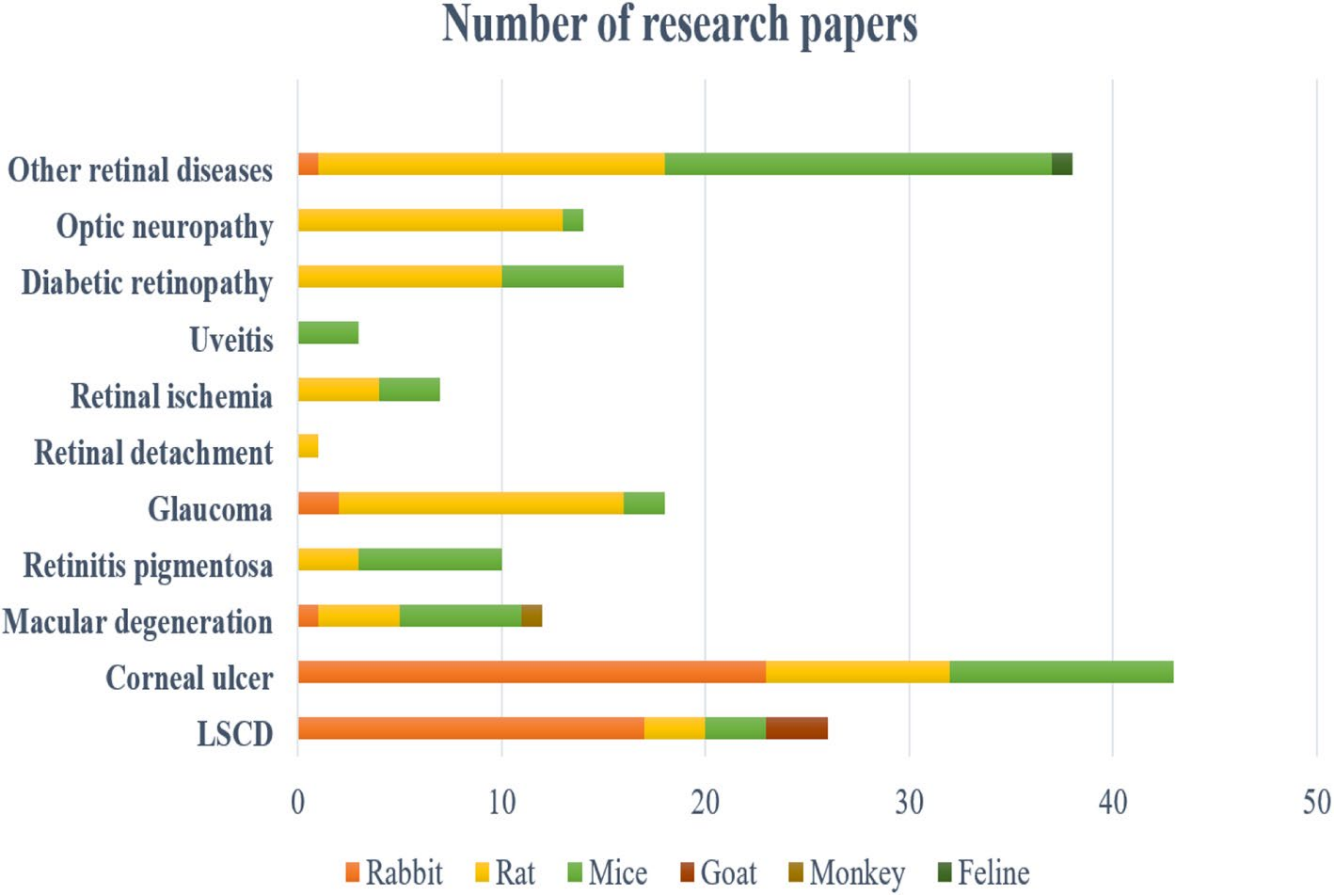
Summary of stem cell trials in ocular affections in animal models

The summary of stem cell trials in ocular affections in animal models is shown in Fig. 2. After reviewing the ocular affections and their available treatment using stem cells, we will discuss an important topic that hinders the cells from reaching the damaged area as the eye is filled with a group of barriers and obstacles and how to overcome these barriers to ensure the drug reaches the correct site in sufficient quantities for effective treatment.

### Barriers and constraints associated with ocular drug delivery

Different ocular barriers separate the eye from the rest of the body and the outside world. The anatomical, physiological, and functional components that shield the eye from the environment and systemic circulation produce these barriers. They are created throughout the development of the embryo, and diseases and aging can alter their integrity and functionality [296, 297]. To shield visual structures from microbial, chemical, metabolic, and mechanical harm, barriers are necessary. They rely on the regulated and active transporters to maintain the transparency of the ocular environment and tissues. Maintaining the makeup of the ocular compartments and occasionally even the intratissue microenvironments depends on this activity. Preserving the immune privilege of various ocular compartments, such as the cornea, aqueous humor, or subretinal space, is one of the main functions of the barrier [297, 298].

### Ocular drug biopharmaceutical barriers

Drugs used topically on the eye must get past several biological barriers to the targeted ocular tissues. First, a precorneal tear film with a total thickness of around 10 µm is used to dilute drug molecules. It is composed of three layers: an outer lipid layer, an intermediate aqueous layer that contains proteins, secreted mucins, salts, and metabolic enzymes, and an interior layer that is 500-nm thick and is primarily made up of lysozymes and cell surface mucins, which together constitute a layer known as glycocalyx [299].

Drugs must either pass through the cornea and/or conjunctiva and reach the internal tissues of the eye, or they must be kept at the target locations of the various ocular diseases. The sclera severely hinders the entry of medicines through the conjunctiva, which is typically linked to systemic drug absorption [300]. Because of this, the cornea serves as the primary entry point for medications whose intended effect is on the inner eye. Regrettably, many medications face significant challenges while trying to pass the corneal barrier [301].

Drug transport is, in fact, extremely challenging due to the hydrophilic stroma and the highly ordered multilayer corneal epithelium. In general, passive diffusion between the various compartments may be the mode of transport, although the existence of inflow and efflux transporters may also be important [302]. In addition to the obstacles mentioned above, medications must permeate through the vitreous humor, an extremely dense matrix made of glycosaminoglycans and collagen fibrils, in order to treat diseases related to the rear of the eye [303]. As an alternative, medications administered topically to the eye can cross the blood–retinal barrier by traveling through the trans-scleral pathway to the choroids [304].

### Influence of physicochemical properties on ocular drug bioavailability

Drugs with low molecular weight lipophilicity can permeate the corneal epithelium via a transcellular route. Subsequently, the medication remains within the stroma, creating a depot from which the medication is discharged into the watery humour. These medications’ sluggish and restricted absorption into the inner eye, as well as the fact that they must be prepared as suspensions or emulsions, which causes patient discomfort and medication loss, are the main issues associated with them [301].

### Advantages offered by nanotechnology in the ocular barrier

The intricacy of these obstacles makes it imperative to logically create distribution vehicles that might aid medications in getting past them. It is possible to create delivery systems specifically designed to get through obstacles related to the eyes, thanks to nanotechnology. Drug-delivery systems using nanoparticles eliminate the need for frequent injections, increase effectiveness, and minimize side effects, all of which improve patient compliance.

The following benefits come from using nanoparticles as carriers for therapeutic agents: the drugs can be delivered to specific cells or tissues, the delivery of large biomolecule and water-insoluble drugs is improved, the drug resistance mechanisms are overcome, the toxicity to healthy tissues is decreased, the high surface to volume ratio of nanoparticles allows to load more drug molecules or multiple drug types, and the blood retention time is prolonged by improving the drug concentration at the pathological sites and by providing sustained drug release. As a result, significant work is being done to create reliable nanocarriers that can deliver drugs safely throughout the BRB in vivo. By providing growth factors as bioactive molecules that activate particular signalling pathways, nanotechnology can control the development of stem cells [305–307].

## Discussion of tables

Table 1 displays the therapeutic efficacy of various MSC types for the treatment of LSCD in many animal models, including rabbit, rat, mouse, and goat. The cornea’s surface was covered with limbal epithelial stem cells, but they could also be grown alongside other stem cells, implanted on type I collagen membrane, in a thermo-reversible polymer, expanded on amniotic membrane, or cultured on polyethylene glycol [13–16]. Additional stem cell types, such as the skin and oral mucosal epithelial stem cells, ADSCs, BMSCs, cord lining epithelial cells, hair follicle-derived stem cells, dental pulp stem cells, iPS cells, and embryonic stem cells that differentiate into corneal epithelial cells when in direct contact with the stroma, are used to treat limbus deficiency [20–24]. The animals’ limbus shows how these stem cells have homed into the limbus. The composite membrane improved the re-epithelialization of the defect area, reduced inflammation and neovascularization, and improved LSC survival, retention, and organization.

Table 2 lists the MSCs used to treat corneal ulcers in rabbits, rats, and mice. According to the available literature, ADSCs are the most commonly used type of MSC, followed by BMSCs in the second stage. Additionally, the transplantation of BMSCs resulted in a successful restoration of the injured corneal surface, and some BMSCs expressed CK3 and assisted in the repair of the corneal epithelium. MSCs may improve epithelial healing while lowering corneal opacification and neovascularization [39]. The scaffold was implanted into the corneal stroma, and there was no significant immune rejection, indicating that the scaffold and corneal tissue were compatible [56]. MSC transplantation was successful in restoring the damaged rat’s eye surface, much like limbal epithelial stem cells were. Instead of MSCs developing into epithelial cells, the prevention of inflammation and angiogenesis after transplantation may be the reason for the therapeutic benefit of transplantation [57].

Table 3 shows how MSCs are utilized to treat macular degeneration in mice, rats, and rabbits. BMSCs, ADSCs, ESCs, and IPSCs are used to treat macular degeneration. Retinal transplanted cells exhibited a slight but discernible B-wave recovery as they multiplied and migrated into the layers of the retina. The transplanted cells displayed the photoreceptor markers Rhodopsin and S-Opsin [76]. When injected into the subretinal area, bone marrow MSCs can differentiate into glial, photoreceptor, and RPE lineage cells [77]. MSC and RPE cell transplantation resulted in an increase in the retina’s outer and inner nuclear layers as well as its overall thickness [86].

Table 4 lists the retinal stem cells, umbilical cord stem cells, ESCs, IPSCs, and BMSCs that are utilized to treat retinitis pigmentosa. Compared to single transplantation, combined transplantation significantly improves the maintenance of electroretinogram outcomes. Furthermore, compared to single transplantation, the ratio of transplanted cells’ photoreceptor growth in the retina of RCS rats receiving combined transplantation was higher [89].

In Table 5, BMSCs are mostly used to treat glaucoma. Additionally, Wharton’s jelly stem cells, UC-BSCs, ESCs, DPSCs, IPSCs, and ADSCs have all been employed. By boosting regulatory and inhibiting proinflammatory cytokines, hWJ-MSCs can control the immune system [97]. In hypertensive eyes, MSC transplantation significantly reduced intraocular pressure [109].

Table 6 shows that ADSCs and BMSCs are mostly utilized in TTT of DR in rats and mice. Neural stem cells are also used as well as the umbilical cord and neural blood. Inflammatory gene expression was downregulated in diabetic retinas treated with ASC [115]. MSC infusion reduced the amount of oxidative damage in the retina and completely prevented the death of retinal ganglion cells [125].

In Table 7, in addition to cord blood cells, BMSCs are mostly used to treat retinal ischemia. BMSCs significantly decreased inflammatory mediators (TNF-α, IL-1β, and IL-6), increased autophagy, and decreased retinal vascular permeability. Both the vitreous and the ischemic retina contained BMSCs [132].

In Table 8, BMSCs, IPSCs are mainly employed in the treatment of uveitis, retinal detachment, whereas both BMSCs and UC-blood cells prevail for the treatment of optic neuropathy, in addition to embryonic stem cells and periodontal ligament cells. MSC infusion significantly reduced experimental auto-immune uveitis [137]. T cells, B cells, dendritic cells, and monocytes were all considerably restored by GMSC. The number of T helper 17 cells was restored, while the proportion of regulatory T cells increased [138]. In retinal detachment models, BMSC transplantation can significantly reduce photoreceptor cell loss and maintain retinal integrity [140]. More neurotrophins are secreted by DPSCs and BMSCs, which promote axon regeneration and RGC survival [149]. Retinal ganglion cells (RGCs) significantly increased after umbilical cord blood stem cells transplantation, although a decline in optic nerve function was significantly inhibited as seen by decreased amplitude drops and peak latency increases of the wave shape [148].

Table 9 lists several stem cell types—BMSCs, ADSCs, UC-Blood, Muller cells, ESCs, IPSCs, and neural and dental pulp stem cells—that are utilized for TTT of retinal degenerative disorders in felines, rabbits, rats, and mice.

For the RGC function to improve and neuroprotection to be successful, the transplanted cells must attach to the retina [155]. While mesenchymal stem cells only offered limited rescue, cells derived from umbilical tissue were able to restore photoreceptors over large areas. Placental cells did not considerably outperform controls, although their effects were comparable.

The highest restoration of photoreceptors was observed in cells derived from umbilical tissue [159]. MSC subretinal implantation preserves retinal function while preventing and delaying retinal ageing. These results suggest that MSC may be a useful source of cells for cell replacement therapies for certain forms of retinal degeneration [160].

Based on these data, we infer that MSCs are the most common form of stem cells used in the treatment of ocular diseases, whether corneal or retinal affections. Among the various forms of mesenchymal stem cells, BMSCs are the most common and widely used cells, followed by ADSCs. ESCs and IPSCs are primarily used in retinal degenerative illnesses, rather than corneal discomfort. Other types of stem cells used in the literature include UC-blood, Wharton’s jelly cells, amniotic membrane epithelial cells, placental stem cells, and dental pulp cells. The most commonly employed cells for treating corneal diseases such as LSCD and corneal ulcers are limbal stem cells, followed by MSCs, mainly BMSCs and ADSCs.

## Conclusions and future directions

Stem cell therapy continues to be an eccentric treatment option for ocular diseases due to the encouraging outcomes of numerous preclinical studies. Cell transplantation is a unique therapeutic strategy for diseases and injuries to the ocular surface and central nervous system (CNS), which includes the retina. Mesenchymal stem cells with several functions possess significant potential for delivering therapeutic agents to treat diseases or injuries in the ocular tissue. In an endeavour to create cell-based treatments, MSC transplantation has garnered a lot of interest. Additionally, MSCs can develop into neural-like cells in vitro, exhibit mature neuronal electrophysiological characteristics, and move and survive when transplanted into CNS tissues. When employed as a therapeutic approach in animal models of glaucoma and retinal degeneration, naive MSCs have also demonstrated the potential to be neuroprotective. The production of neurotrophic growth factors by stem cell engineering has been investigated as a promising long-term delivery method for neuroprotective agents to the wounded central nervous system in many animal models. With the promising results of multiple preclinical studies, stem cell therapy is still a great choice for treating ocular degenerative illnesses. However, medication transport to the posterior ocular area is severely restricted by a number of anatomical and physiological barriers in the eye. Potential future therapeutic approaches to enhance the clinical outcomes could involve co-transplanting two or more cell types. Additionally, using nanotechnology in combination with other delivery methods has improved the delivery of ocular medications. Furthermore, using scaffolds in the culture and transplantation processes may enhance the therapeutic effects of stem cells and their derivatives.

## Data Availability

All data collected or analyzed during this study are included in this published review.

## Abbreviations

MSCs: Mesenchymal stem cells
ADSCs: Adipose tissue-derived stem/stromal cells
BMSCs: Bone marrow mesenchymal stem cells
HSCs: Hematopoietic stem cells
ESCs: Embryonic stem cells
IPSCs: Induced pluripotent stem cells
DPSCs: Dental pulp-derived stem cells
UC-MSCs: Umbilical cord mesenchymal stem cells
AM-MSCs: Amniotic membrane mesenchymal stem cells
NSCs: Neural stem cells
LSCs: Limbal stem cells
MSCEVs: MSC-derived extracellular vesicles
LEC: Limbal epithelial cells
RGC: Retinal ganglion cells
RPE: Retinal pigmented epithelium
Oct-3/4: Octamer binding transcription factor
Sox2: SRY-Box Transcription Factor 2
c-Myc: Cellular-myelocytomatosis
Klf4: Krüppel-like factor 4
LSCD: Limbal stem cell deficiency
DM: Diabetes mellitus
RDDs: Retinal degenerative diseases
DR: Diabetic retinopathy
NPDR: Non-proliferative diabetic retinopathy
PDR: Proliferative diabetic retinopathy
RP: Retinitis pigmentosa
AMD: Age-related macular degeneration
RD: Retinal detachment
RI: Retinal ischemia
I/R: Ischemia reperfusion
AU: Autoimmune uveitis
CNS: Central nervous system
ON: Optic nerve
BRB: Blood–retinal barrier
IOP: Intraocular pressure
CD: Clusters of differentiation
HLA-DR: Human leucocyte antigen–D related
PDGF: Platelet-derived growth factor
VEGF: Vascular endothelial growth factor
HGF: Hepatocyte growth factor
TGFβ1: Transforming growth factor beta 1
IL6,10: Interleukins 6,10
IFN-γ: Interferon-γ
NTFs: Neurotrophic factors
CNTF: Ciliary neurotrophic factor
GDNF: Glial-derived neurotrophic factor
NGF: Nerve growth factor
FGF: Basic fibroblast growth factor
EGF: Epidermal growth factor
IGF: Insulin growth factor
BDNF: Brain-derived neurotrophic factor
NT-3, 4/5: Neurotrophin-3, 4/5
EPO: Erythropoietin
IDO: Indole amine 2,3-dioxygenase
NO: Nitric oxide
ROS: Reactive oxygen species
PGE2: Prostaglandin E2
TSP1: Thrombospondin type 1

## References

[1] Kim YC, et al. Ocular delivery of macromolecules. J Control Release. 2014;190:172–81. 24998941 10.1016/j.jconrel.2014.06.043 PMC4142116

[2] Gower NJ, et al. Drug discovery in ophthalmology: past success, present challenges, and future opportunities. BMC Ophthalmol. 2016;16:1–11. 26774505 10.1186/s12886-016-0188-2 PMC4715274

[3] De La Mata A, et al. Chitosan–gelatin biopolymers as carrier substrata for limbal epithelial stem cells. J Mater Sci - Mater Med. 2013;24:2819–29. 23892486 10.1007/s10856-013-5013-3

[4] Villegas VM, et al. Current advances in the treatment of neovascular age-related macular degeneration. Expert Opin Drug Deliv. 2017;14(2):273–82. 27434329 10.1080/17425247.2016.1213240

[5] Gater R, et al. Development of better treatments for retinal disease using stem cell therapies. Int J Stem Cell Res Ther. 2016;3:1–6.

[6] Whalen M, et al. Seeing the future: a review of ocular therapy. Bioengineering. 2024;11(2):179. 38391665 10.3390/bioengineering11020179 PMC10886198

[7] Rama P, et al. Limbal stem-cell therapy and long-term corneal regeneration. N Engl J Med. 2010;363(2):147–55. 20573916 10.1056/NEJMoa0905955

[8] Tomczak W, et al. Advancements in ocular regenerative therapies Biology. 2023;12(5):737. 37237549 10.3390/biology12050737 PMC10215726

[9] Adak, S., et al., A review on mesenchymal stem cells for treatment of retinal diseases. Stem cell reviews and reports, 2021: p. 1–20. 10.1007/s12015-020-10090-x PMC7787584 33410097

[10] Jeong W-Y, Kim J-H, Kim C-W. Co-culture of human bone marrow mesenchymal stem cells and macrophages attenuates lipopolysaccharide-induced inflammation in human corneal epithelial cells. Biosci Biotechnol Biochem. 2018;82(5):800–9. 29452534 10.1080/09168451.2018.1438167

[11] Shang Q, et al. Adipose-derived mesenchymal stromal cells promote corneal wound healing by accelerating the clearance of neutrophils in cornea. Cell Death Dis. 2020;11(8):707. 32848141 10.1038/s41419-020-02914-y PMC7450061

[12] Sharma J, et al. Corneal reconstruction in chemically damaged cornea using temperature responsive surface assisted mesenchymal stem cell transplantation in rabbits. Graefes Arch Clin Exp Ophthalmol. 2021;259:1859–70. 33754210 10.1007/s00417-021-05132-0

[13] Ti S, et al. Correlation of long term phenotypic and clinical outcomes following limbal epithelial transplantation cultivated on amniotic membrane in rabbits. Br J Ophthalmol. 2004;88(3):422–7. 14977781 10.1136/bjo.2003.026054 PMC1772037

[14] Qu L, et al. Reconstruction of corneal epithelium with cryopreserved corneal limbal stem cells in a rabbit model. Vet J. 2009;179(3):392–400. 10.1016/j.tvjl.2007.10.009 18023216

[15] Sitalakshmi G, et al. Ex vivo cultivation of corneal limbal epithelial cells in a thermoreversible polymer (Mebiol Gel) and their transplantation in rabbits: an animal model. Tissue Eng Part A. 2009;15(2):407–15. 10.1089/ten.tea.2008.0041 18724830

[16] Omoto M, et al. The use of human mesenchymal stem cell–derived feeder cells for the cultivation of transplantable epithelial sheets. Invest Ophthalmol Vis Sci. 2009;50(5):2109–15. 10.1167/iovs.08-2262 19136703

[17] Monteiro B, et al. Human immature dental pulp stem cells share key characteristic features with limbal stem cells. Cell Prolif. 2009;42(5):587–94. 10.1111/j.1365-2184.2009.00623.x PMC6495697 19614680

[18] Gomes JÁP, et al. Corneal reconstruction with tissue-engineered cell sheets composed of human immature dental pulp stem cells. Invest Ophthalmol Vis Sci. 2010;51(3):1408–14. 10.1167/iovs.09-4029 19892864

[19] Reinshagen H, et al. Corneal surface reconstruction using adult mesenchymal stem cells in experimental limbal stem cell deficiency in rabbits. Acta Ophthalmol. 2011;89(8):741–8. 10.1111/j.1755-3768.2009.01812.x 20039850

[20] Reza HM, et al. Umbilical cord lining stem cells as a novel and promising source for ocular surface regeneration. Stem Cell Rev Rep. 2011;7:935–47. 10.1007/s12015-011-9245-7 21431286

[21] Zhang W, et al. Rapidly constructed scaffold‐free embryonic stem cell sheets for ocular surface reconstruction. Scanning. 2014;36(3):286–92. 10.1002/sca.21103 23861021

[22] Sugiyama H, et al. Evidence of the survival of ectopically transplanted oral mucosal epithelial stem cells after repeated wounding of cornea. Mol Ther. 2014;22(8):1544–55. 24769908 10.1038/mt.2014.69 PMC4435588

[23] Ouyang H, et al. WNT7A and PAX6 define corneal epithelium homeostasis and pathogenesis. Nature. 2014;511(7509):358–61. 25030175 10.1038/nature13465 PMC4610745

[24] Hayashi R, et al. Co-ordinated ocular development from human iPS cells and recovery of corneal function. Nature. 2016;531(7594):376–80. 26958835 10.1038/nature17000

[25] Li Y, et al. Poly (ethylene glycol)-modified silk fibroin membrane as a carrier for limbal epithelial stem cell transplantation in a rabbit LSCD model. Stem Cell Res Ther. 2017;8:1–19. 29116027 10.1186/s13287-017-0707-y PMC5678789

[26] Zhou Z, et al. Nanofiber-reinforced decellularized amniotic membrane improves limbal stem cell transplantation in a rabbit model of corneal epithelial defect. Acta Biomater. 2019;97:310–20. 31437637 10.1016/j.actbio.2019.08.027

[27] Yu Y, et al. Matrix-assisted cell transplantation for the treatment of limbal stem cell deficiency in a rabbit model. Biomedicines. 2024;12(1):101. 38255207 10.3390/biomedicines12010101 PMC10813050

[28] Rohaina CM, et al. Reconstruction of limbal stem cell deficient corneal surface with induced human bone marrow mesenchymal stem cells on amniotic membrane. Transl Res. 2014;163(3):200–10. 24286920 10.1016/j.trsl.2013.11.004

[29] Irani YD, et al. Oral mucosal epithelial cells grown on porous silicon membrane for transfer to the rat eye. Sci Rep. 2017;7(1):10042. 28855664 10.1038/s41598-017-10793-1 PMC5577150

[30] Sun J, et al. Differentiation of rat adipose-derived mesenchymal stem cells into corneal-like epithelial cells driven by PAX6. Exp Ther Med. 2018;15(2):1424–32. 29434727 10.3892/etm.2017.5576 PMC5774412

[31] Nieto-Nicolau N, et al. Priming human adipose-derived mesenchymal stem cells for corneal surface regeneration. J Cell Mol Med. 2021;25(11):5124–37. 33951289 10.1111/jcmm.16501 PMC8178265

[32] Zajicova A, et al. Treatment of ocular surface injuries by limbal and mesenchymal stem cells growing on nanofiber scaffolds. Cell Transplant. 2010;19(10):1281–90. 20573307 10.3727/096368910X509040

[33] Meyer-Blazejewska EA, et al. From hair to cornea: toward the therapeutic use of hair follicle-derived stem cells in the treatment of limbal stem cell deficiency. Stem Cells. 2011;29(1):57–66. 10.1002/stem.550 PMC3711469 20957740

[34] Yang X, et al. Plasticity of epidermal adult stem cells derived from adult goat ear skin. Mol Reprod Dev. 2007;74(3):386–96. 10.1002/mrd.20598 16998851

[35] Yang X, et al. Reconstruction of damaged cornea by autologous transplantation of epidermal adult stem cells. Mol Vis. 2008;14:1064. 18552982 PMC2426721

[36] Mi S, et al. Reconstruction of corneal epithelium with cryopreserved corneal limbal stem cells in a goat model. Mol Reprod Dev. 2008;75(11):1607–16. 10.1002/mrd.20900 18361397

[37] Ye J, Yao K, Kim J. Mesenchymal stem cell transplantation in a rabbit corneal alkali burn model: engraftment and involvement in wound healing. Eye. 2006;20(4):482–90. 15895027 10.1038/sj.eye.6701913

[38] Park K-S, et al. The side population cells in the rabbit limbus sensitively increased in response to the central cornea wounding. Invest Ophthalmol Vis Sci. 2006;47(3):892–900. 16505021 10.1167/iovs.05-1006

[39] Gu S, et al. Differentiation of rabbit bone marrow mesenchymal stem cells into corneal epithelial cells in vivo and ex vivo. Mol Vis. 2009;15:99. 19156227 PMC2627808

[40] Espandar L, et al. Adipose-derived stem cells on hyaluronic acid–derived scaffold: a new horizon in bioengineered cornea. Arch Ophthalmol. 2012;130(2):202–8. 22332213 10.1001/archopthalmol.2011.1398

[41] Kim T-H, et al. Effects of conditioned media from human amniotic epithelial cells on corneal alkali injuries in rabbits. J Vet Sci. 2013;14(1):61. 23388445 10.4142/jvs.2013.14.1.61 PMC3615233

[42] Lin HF, et al. Effects of cultured human adipose-derived stem cells transplantation on rabbit cornea regeneration after alkaline chemical burn. Kaohsiung J Med Sci. 2013;29(1):14–8. 23257251 10.1016/j.kjms.2012.08.002 PMC11916480

[43] Espandar L, et al. Application of adipose-derived stem cells on scleral contact lens carrier in an animal model of severe acute alkaline burn. Eye Contact Lens. 2014;40(4):243–7. 24901976 10.1097/ICL.0000000000000045 PMC4365910

[44] del Barrio JLA, et al. Acellular human corneal matrix sheets seeded with human adipose-derived mesenchymal stem cells integrate functionally in an experimental animal model. Invest Ophthalmol Vis Sci. 2014;55(13):5158. 10.1016/j.exer.2015.01.020 25625506

[45] Zeng W, et al. Transplantation with cultured stem cells derived from the human amniotic membrane for corneal alkali burns: an experimental study. Ann Clin Lab Sci. 2014;44(1):73–81. 24695478

[46] Ahmed SK, et al. Bone marrow mesenchymal stem cell transplantation in a rabbit corneal alkali burn model (a histological and immune histo-chemical study). Int J Stem Cells. 2015;8(1):69–78. 26019756 10.15283/ijsc.2015.8.1.69 PMC4445711

[47] Almaliotis D, et al. Mesenchymal stem cells improve healing of the cornea after alkali injury. Graefes Arch Clin Exp Ophthalmol. 2015;253:1121–35. 26002143 10.1007/s00417-015-3042-y

[48] Fuentes-Julián S, et al. Adipose-derived mesenchymal stem cell administration does not improve corneal graft survival outcome. PLoS ONE. 2015;10(3):e0117945. 25730319 10.1371/journal.pone.0117945 PMC4346399

[49] Alió del Barrio JL, et al. Biointegration of corneal macroporous membranes based on poly (ethyl acrylate) copolymers in an experimental animal model. J Biomed Mater Res A. 2015;103(3):1106–18. 10.1002/jbm.a.35249 24910285

[50] Holan V, et al. A comparative study of the therapeutic potential of mesenchymal stem cells and limbal epithelial stem cells for ocular surface reconstruction. Stem Cells Transl Med. 2015;4(9):1052–63. 26185258 10.5966/sctm.2015-0039 PMC4542873

[51] Navas A, et al. Anti-inflammatory and anti-fibrotic effects of human amniotic membrane mesenchymal stem cells and their potential in corneal repair. Stem Cells Transl Med. 2018;7(12):906–17. 30260581 10.1002/sctm.18-0042 PMC6265633

[52] Yamashita K, et al. Corneal endothelial regeneration using mesenchymal stem cells derived from human umbilical cord. Stem Cells Dev. 2018;27(16):1097–108. 29929442 10.1089/scd.2017.0297

[53] Xu W, et al. An in situ hydrogel based on carboxymethyl chitosan and sodium alginate dialdehyde for corneal wound healing after alkali burn. J Biomed Mater Res A. 2019;107(4):742–54. 10.1002/jbm.a.36589 PMC6590378 30548137

[54] Almaliotis D, et al. Evaluation of clinical and histological outcomes of adipose-derived mesenchymal stem cells in a rabbit corneal alkali burn model. Stem Cells Int. 2021;2021. 10.1155/2021/6610023 PMC7964115 33763139

[55] Park I-S, et al. Corneal repair with adhesive cell sheets of fetal cartilage-derived stem cells. Tissue Eng Regen Med. 2021;18:187–98. 10.1007/s13770-020-00317-w PMC7862470 33415672

[56] Li Y, et al. 3D biomaterial P scaffolds carrying umbilical cord mesenchymal stem cells improve biointegration of keratoprosthesis. Biomed Mater. 2022;17(5):055004. 10.1088/1748-605X/ac7e98 35790151

[57] Ma Y, et al. Reconstruction of chemically burned rat corneal surface by bone marrow–derived human mesenchymal stem cells. Stem Cells. 2006;24(2):315–21. 10.1634/stemcells.2005-0046 16109757

[58] Oh JY, et al. The anti-inflammatory and anti-angiogenic role of mesenchymal stem cells in corneal wound healing following chemical injury. Stem Cells. 2008;26(4):1047–55. 10.1634/stemcells.2007-0737 18192235

[59] Jiang T-S, et al. Reconstruction of the corneal epithelium with induced marrow mesenchymal stem cells in rats. Mol Vis. 2010;16:1304. 20664793 PMC2905634

[60] Yao L, et al. Role of mesenchymal stem cells on cornea wound healing induced by acute alkali burn. PLoS ONE. 2012;7(2):e30842. 10.1371/journal.pone.0030842 PMC3281878 22363499

[61] Zeppieri M, et al. Human adipose-derived stem cells for the treatment of chemically burned rat cornea: preliminary results. Curr Eye Res. 2013;38(4):451–63. 23373736 10.3109/02713683.2012.763100

[62] Ke Y, et al. Polysaccharide hydrogel combined with mesenchymal stem cells promotes the healing of corneal alkali burn in rats. PLoS ONE. 2015;10(3):e0119725. 25789487 10.1371/journal.pone.0119725 PMC4366244

[63] Dinç E, et al. Evaluation of anti-inflammatory and antiapoptotic effects of bone marrow and adipose-derived mesenchymal stem cells in acute alkaline corneal burn. J Ocul Pharmacol Ther. 2021;37(1):24–34. 10.1089/jop.2020.0103 33275515

[64] El-Din WN, Nooreldin N, Essawy A. The potential therapeutic efficacy of intravenous versus subconjunctival mesenchymal stem cells on experimentally ultraviolet-induced corneal injury in adult male albino rats. Folia Morphol. 2022;81(4):900–16. 10.5603/FM.a2021.0085 34545564

[65] Fu Y-S, et al. Human umbilical mesenchymal stem cell xenografts repair UV-induced photokeratitis in a rat model. Biomedicines. 2022;10(5):1125. 35625862 10.3390/biomedicines10051125 PMC9138504

[66] Homma R, et al. Induction of epithelial progenitors in vitro from mouse embryonic stem cells and application for reconstruction of damaged cornea in mice. Invest Ophthalmol Vis Sci. 2004;45(12):4320–6. 15557438 10.1167/iovs.04-0044

[67] Ueno H, et al. Experimental transplantation of corneal epithelium-like cells induced by Pax6 gene transfection of mouse embryonic stem cells. Cornea. 2007;26(10):1220–7. 18043180 10.1097/ICO.0b013e31814fa814

[68] Kumagai Y, et al. Induction of corneal epithelium–like cells from cynomolgus monkey embryonic stem cells and their experimental transplantation to damaged cornea. Cornea. 2010;29(4):432–8. 20164754 10.1097/ICO.0b013e3181b9ffcc

[69] Lin K-J, et al. Topical administration of orbital fat-derived stem cells promotes corneal tissue regeneration. Stem Cell Res Ther. 2013;4:1–12. 23769140 10.1186/scrt223 PMC3707029

[70] Basu S, et al. Human limbal biopsy–derived stromal stem cells prevent corneal scarring. Sci Transl Med. 2014;6(266):266ra172. 10.1126/scitranslmed.3009644 PMC4398334 25504883

[71] Mittal SK, et al. Restoration of corneal transparency by mesenchymal stem cells. Stem Cell Rep. 2016;7(4):583–90. 10.1016/j.stemcr.2016.09.001 PMC5063582 27693426

[72] Yang J, et al. Universal corneal epithelial-like cells derived from human embryonic stem cells for cellularization of a corneal scaffold. Transl Vis Sci Technol. 2018;7(5):23. 10.1167/tvst.7.5.23 PMC6181193 30323996

[73] Shukla S, et al. Therapeutic efficacy of different routes of mesenchymal stem cell administration in corneal injury. Ocul Surf. 2019;17(4):729–36. 31279065 10.1016/j.jtos.2019.07.005 PMC6874749

[74] Putra I, et al. Preclinical evaluation of the safety and efficacy of cryopreserved bone marrow mesenchymal stromal cells for corneal repair. Transl Vis Sci Technol. 2021;10(10):3. 10.1167/tvst.10.10.3 PMC8362636 34383879

[75] Chen M, et al. Subconjunctival administration of mesenchymal stem cells alleviates ocular inflammation in a murine model of corneal alkali burn. Stem Cells. 2023;41(6):592–602. 37061809 10.1093/stmcls/sxad027

[76] Amirpour N, et al. Differentiation of human embryonic stem cell–derived retinal progenitors into retinal cells by sonic hedgehog and/or retinal pigmented epithelium and transplantation into the subretinal space of sodium iodate–injected rabbits. Stem Cells Dev. 2012;21(1):42–53. 10.1089/scd.2011.0073 21456900

[77] Gong L, et al. Differentiation of rat mesenchymal stem cells transplanted into the subretinal space of sodium iodate-injected rats. Clin Exp Ophthalmol. 2008;36(7):666–71. 10.1111/j.1442-9071.2008.01857.x 18983552

[78] Park UC, et al. Subretinal transplantation of putative retinal pigment epithelial cells derived from human embryonic stem cells in rat retinal degeneration model. Clin Exp Reprod Med. 2011;38(4):216. 22384445 10.5653/cerm.2011.38.4.216 PMC3283081

[79] Guan Y, et al. Subretinal transplantation of rat MSCs and erythropoietin gene modified rat MSCs for protecting and rescuing degenerative retina in rats. Curr Mol Med. 2013;13(9):1419–31. 23971737 10.2174/15665240113139990071

[80] Tsai Y, et al. Human iPSC-derived neural progenitors preserve vision in an AMD-like model. Stem Cells. 2015;33(8):2537–49. 25869002 10.1002/stem.2032 PMC5477659

[81] Otani A, et al. Bone marrow–derived stem cells target retinal astrocytes and can promote or inhibit retinal angiogenesis. Nat Med. 2002;8(9):1004–10. 12145646 10.1038/nm744

[82] Machalińska A, et al. Long-term neuroprotective effects of NT-4–engineered mesenchymal stem cells injected intravitreally in a mouse model of acute retinal injury. Invest Ophthalmol Vis Sci. 2013;54(13):8292–305. 24265016 10.1167/iovs.13-12221

[83] Jiang Y, et al. Therapeutic effect of bone marrow mesenchymal stem cells on laser-induced retinal injury in mice. Int J Mol Sci. 2014;15(6):9372–85. 24871366 10.3390/ijms15069372 PMC4100100

[84] Shirai H, et al. Transplantation of human embryonic stem cell-derived retinal tissue in two primate models of retinal degeneration. Proc Natl Acad Sci. 2016;113(1):E81–90. 26699487 10.1073/pnas.1512590113 PMC4711854

[85] Barzelay A, et al. Adipose-derived mesenchymal stem cells migrate and rescue RPE in the setting of oxidative stress. Stem Cells Int. 2018;2018(1):9682856. 10.1155/2018/9682856 PMC6311721 30651740

[86] Pan T, et al. Combined transplantation with human mesenchymal stem cells improves retinal rescue effect of human fetal RPE cells in retinal degeneration mouse model. Invest Ophthalmol Vis Sci. 2020;61(8):9. 32639552 10.1167/iovs.61.8.9 PMC7425709

[87] Wang S, et al. Non-invasive stem cell therapy in a rat model for retinal degeneration and vascular pathology. PLoS ONE. 2010;5(2):e9200. 10.1371/journal.pone.0009200 PMC2821411 20169166

[88] Jian Q, Li Y, Yin ZQ. Rat BMSCs initiate retinal endogenous repair through NGF/TrkA signaling. Exp Eye Res. 2015;132:34–47. 25584870 10.1016/j.exer.2015.01.008

[89] Qu L, et al. Combined transplantation of human mesenchymal stem cells and human retinal progenitor cells into the subretinal space of RCS rats. Sci Rep. 2017;7(1):199. 28298640 10.1038/s41598-017-00241-5 PMC5428026

[90] Arnhold S, et al. Transplantation of bone marrow-derived mesenchymal stem cells rescue photoreceptor cells in the dystrophic retina of the rhodopsin knockout mouse. Graefes Arch Clin Exp Ophthalmol. 2007;245:414–22. 16896916 10.1007/s00417-006-0382-7

[91] Canola K, et al. Retinal stem cells transplanted into models of late stages of retinitis pigmentosa preferentially adopt a glial or a retinal ganglion cell fate. Invest Ophthalmol Vis Sci. 2007;48(1):446–54. 17197566 10.1167/iovs.06-0190 PMC2823590

[92] Wang N-K, et al. Transplantation of reprogrammed embryonic stem cells improves visual function in a mouse model for retinitis pigmentosa. Transplantation. 2010;89(8):911–9. 20164818 10.1097/TP.0b013e3181d45a61 PMC2855750

[93] Tucker BA, et al. Patient-specific iPSC-derived photoreceptor precursor cells as a means to investigate retinitis pigmentosa. elife. 2013;2:e00824. 10.7554/eLife.00824 PMC3755341 23991284

[94] Barnea-Cramer AO, et al. Function of human pluripotent stem cell-derived photoreceptor progenitors in blind mice. Sci Rep. 2016;6(1):29784. 27405580 10.1038/srep29784 PMC4942817

[95] Brown C, et al. Human primitive mesenchymal stem cell-derived retinal progenitor cells improved neuroprotection, neurogenesis, and vision in rd12 mouse model of retinitis pigmentosa. Stem Cell Res Ther. 2022;13(1):148. 35395806 10.1186/s13287-022-02828-w PMC8994263

[96] Sihota R, et al. Effect of intracameral human cord blood-derived stem cells on lasered rabbit trabecular meshwork. Int Ophthalmol. 2019;39:2757–66. 31140023 10.1007/s10792-019-01120-w

[97] dos Santos Evangelho K, et al. Mesenchymal stromal cells from human Wharton’s jelly modulate the intraocular immune response in a glucocorticoid hypertension model: an exploratory analysis. Ophthalmic Res. 2024;67(1):232–47. 10.1159/000538183 38447539

[98] Yu S, et al. Effects of bone marrow stromal cell injection in an experimental glaucoma model. Biochem Biophys Res Commun. 2006;344(4):1071–9. 16643846 10.1016/j.bbrc.2006.03.231

[99] Bull ND, Limb GA, Martin KR. Human Muller stem cell (MIO-M1) transplantation in a rat model of glaucoma: survival, differentiation, and integration. Invest Ophthalmol Vis Sci. 2008;49(8):3449–56. 18408183 10.1167/iovs.08-1770

[100] Johnson TV, et al. Neuroprotective effects of intravitreal mesenchymal stem cell transplantation in experimental glaucoma. Invest Ophthalmol Vis Sci. 2010;51(4):2051–9. 19933193 10.1167/iovs.09-4509 PMC2868400

[101] Harper MM, et al. Transplantation of BDNF-secreting mesenchymal stem cells provides neuroprotection in chronically hypertensive rat eyes. Invest Ophthalmol Vis Sci. 2011;52(7):4506–15. 21498611 10.1167/iovs.11-7346 PMC3175938

[102] Zhou X, Xia X-B. Retinal stem cells transplantation combined with copolymer-1 immunization reduces interferon-gamma levels in an experimental model of glaucoma. Int J Ophthalmol. 2011;4(6):594. 22553727 10.3980/j.issn.2222-3959.2011.06.04 PMC3340800

[103] Zhou X, Xia X-B, Xiong S-Q. Neuro-protection of retinal stem cells transplantation combined with copolymer-1 immunization in a rat model of glaucoma. Mol Cell Neurosci. 2013;54:1–8. 23246669 10.1016/j.mcn.2012.12.001

[104] Hu Y, et al. Bone marrow mesenchymal stem cells protect against retinal ganglion cell loss in aged rats with glaucoma. Clin Interv Aging. 2013:1467–70. 10.2147/CIA.S47350 PMC3817004 24204132

[105] Johnson TV, et al. Identification of retinal ganglion cell neuroprotection conferred by platelet-derived growth factor through analysis of the mesenchymal stem cell secretome. Brain. 2013;137(2):503–19. 24176979 10.1093/brain/awt292 PMC3914467

[106] Manuguerra-GagnÉ R, et al. Transplantation of mesenchymal stem cells promotes tissue regeneration in a glaucoma model through laser-induced paracrine factor secretion and progenitor cell recruitment. Stem Cells. 2013;31(6):1136–48. 23495088 10.1002/stem.1364

[107] Emre E, et al. Neuroprotective effects of intravitreally transplanted adipose tissue and bone marrow–derived mesenchymal stem cells in an experimental ocular hypertension model. Cytotherapy. 2015;17(5):543–59. 25618560 10.1016/j.jcyt.2014.12.005

[108] Parameswaran S, et al. Continuous non-cell autonomous reprogramming to generate retinal ganglion cells for glaucomatous neuropathy. Stem Cells. 2015;33(6):1743–58. 25753398 10.1002/stem.1987 PMC4524556

[109] Roubeix C, et al. Intraocular pressure reduction and neuroprotection conferred by bone marrow-derived mesenchymal stem cells in an animal model of glaucoma. Stem Cell Res Ther. 2015;6:1–13. 26377305 10.1186/s13287-015-0168-0 PMC4574127

[110] Mead B, et al. Mesenchymal stromal cell–mediated neuroprotection and functional preservation of retinal ganglion cells in a rodent model of glaucoma. Cytotherapy. 2016. 10.1016/j.jcyt.2015.12.002 26897559

[111] Divya MS, et al. Intraocular injection of ES cell-derived neural progenitors improve visual function in retinal ganglion cell-depleted mouse models. Front Cell Neurosci. 2017;11:295. 28979193 10.3389/fncel.2017.00295 PMC5611488

[112] Zhu W, et al. Restoration of aqueous humor outflow following transplantation of iPSC-derived trabecular meshwork cells in a transgenic mouse model of glaucoma. Invest Ophthalmol Vis Sci. 2017;58(4):2054–62. 28384726 10.1167/iovs.16-20672 PMC6108236

[113] Yang Z, et al. Amelioration of diabetic retinopathy by engrafted human adipose-derived mesenchymal stem cells in streptozotocin diabetic rats. Graefes Arch Clin Exp Ophthalmol. 2010;248:1415–22. 20437245 10.1007/s00417-010-1384-z

[114] El Maadawi ZM, Gabr HM. Effect of human cord blood-derived stem cells on induced diabetic retinopathy in adult albino rat: histological and immunohistochemical study. Egypt J Histol. 2011;34(3):576–85.

[115] Rajashekhar G, et al. Regenerative therapeutic potential of adipose stromal cells in early stage diabetic retinopathy. PLoS ONE. 2014;9(1):e84671. 10.1371/journal.pone.0084671 PMC3886987 24416262

[116] Cerman E, et al. Retinal electrophysiological effects of intravitreal bone marrow derived mesenchymal stem cells in streptozotocin induced diabetic rats. PLoS ONE. 2016;11(6):e0156495. 10.1371/journal.pone.0156495 PMC4907488 27300133

[117] Kim JM, et al. Perivascular progenitor cells derived from human embryonic stem cells exhibit functional characteristics of pericytes and improve the retinal vasculature in a rodent model of diabetic retinopathy. Stem Cells Transl Med. 2016;5(9):1268–76. 27388242 10.5966/sctm.2015-0342 PMC4996442

[118] Fathy El Mongy N, et al. Effects of adipose tissue mesenchymal stem cell therapy on diabetic rats. Al-Azhar Med J. 2017;46(1):193–210.

[119] Zhang W, et al. Therapeutic efficacy of neural stem cells originating from umbilical cord-derived mesenchymal stem cells in diabetic retinopathy. Sci Rep. 2017;7(1):408. 28341839 10.1038/s41598-017-00298-2 PMC5412648

[120] Abdel-Kawi SH, Hashem KS. Administration of melatonin in diabetic retinopathy is effective and improves the efficacy of mesenchymal stem cell treatment. Stem Cells Int. 2022;2022(1):6342594. 10.1155/2022/6342594 PMC9017455 35450343

[121] Kang N, et al. Beneficial effect of sirolimus-pretreated mesenchymal stem cell implantation on diabetic retinopathy in rats. Biomedicines. 2024;12(2):383. 10.3390/biomedicines12020383 PMC10886997 38397985

[122] Imbarak N, et al. Effect of bone marrow mesenchymal stem cells on a short-term induced diabetic retinopathy in adult female albino rats. Regen Eng Transl Med. 2024;10(1):93–109.

[123] Mendel TA, et al. Pericytes derived from adipose-derived stem cells protect against retinal vasculopathy. PLoS ONE. 2013;8(5):e65691. 10.1371/journal.pone.0065691 PMC3669216 23741506

[124] Cronk SM, et al. Adipose-derived stem cells from diabetic mice show impaired vascular stabilization in a murine model of diabetic retinopathy. Stem Cells Transl Med. 2015;4(5):459–67. 25769654 10.5966/sctm.2014-0108 PMC4414213

[125] Ezquer M, et al. Intravitreal administration of multipotent mesenchymal stromal cells triggers a cytoprotective microenvironment in the retina of diabetic mice. Stem Cell Res Ther. 2016;7:1–17. 26983784 10.1186/s13287-016-0299-y PMC4793534

[126] Elshaer SL, et al. Adipose stem cells and their paracrine factors are therapeutic for early retinal complications of diabetes in the Ins2 Akita mouse. Stem Cell Res Ther. 2018;9:1–18. 30463601 10.1186/s13287-018-1059-y PMC6249931

[127] Rong L, et al. Bone marrow CD133+ stem cells ameliorate visual dysfunction in streptozotocin-induced diabetic mice with early diabetic retinopathy. Cell Transplant. 2018;27(6):916–36. 29717657 10.1177/0963689718759463 PMC6050916

[128] Cheung KW, et al. Analysis of the retinal capillary plexus layers in a murine model with diabetic retinopathy: effect of intravitreal injection of human CD34+ bone marrow stem cells. Ann Transl Med. 2021;9(15). 10.21037/atm-20-3930 PMC8421965 34532410

[129] Na L, Xiao-rong L, Jia-qin Y. Effects of bone-marrow mesenchymal stem cells transplanted into vitreous cavity of rat injured by ischemia/reperfusion. Graefes Arch Clin Exp Ophthalmol. 2009;247:503–14. 19084985 10.1007/s00417-008-1009-y

[130] Dreixler JC, et al. Delayed administration of bone marrow mesenchymal stem cell conditioned medium significantly improves outcome after retinal ischemia in rats. Invest Ophthalmol Vis Sci. 2014;55(6):3785–96. 24699381 10.1167/iovs.13-11683 PMC4062399

[131] Roth S, et al. Hypoxic-preconditioned bone marrow stem cell medium significantly improves outcome after retinal ischemia in rats. Invest Ophthalmol Vis Sci. 2016;57(7):3522–32. 27367588 10.1167/iovs.15-17381 PMC4961056

[132] Mathew B, et al. Bone-marrow mesenchymal stem-cell administration significantly improves outcome after retinal ischemia in rats. Graefes Arch Clin Exp Ophthalmol. 2017;255:1581–92. 28523456 10.1007/s00417-017-3690-1 PMC5841582

[133] Nguyen H, et al. Eye opener in stroke: mitochondrial dysfunction and stem cell repair in retinal ischemia. Stroke. 2019;50(8):2197–206. 31242827 10.1161/STROKEAHA.119.025249 PMC6650274

[134] Ritter MR, et al. Myeloid progenitors differentiate into microglia and promote vascular repair in a model of ischemic retinopathy. J Clin Investig. 2006;116(12):3266–76. 17111048 10.1172/JCI29683 PMC1636693

[135] Park TS, et al. Vascular progenitors from cord blood–derived induced pluripotent stem cells possess augmented capacity for regenerating ischemic retinal vasculature. Circulation. 2014;129(3):359–72. 24163065 10.1161/CIRCULATIONAHA.113.003000 PMC4090244

[136] Minhas G, et al. Transplantation of lineage-negative stem cells in pterygopalatine artery ligation induced retinal ischemia–reperfusion injury in mice. Mol Cell Biochem. 2017;429:123–36. 28210901 10.1007/s11010-017-2941-0

[137] Tasso R, et al. Mesenchymal stem cells induce functionally active T-regulatory lymphocytes in a paracrine fashion and ameliorate experimental autoimmune uveitis. Invest Ophthalmol Vis Sci. 2012;53(2):786–93. 22232435 10.1167/iovs.11-8211 PMC3317420

[138] Gao Y, et al. Single-cell analysis of immune cells on gingiva-derived mesenchymal stem cells in experimental autoimmune uveitis. iScience. 2023;26(5). 10.1016/j.isci.2023.106729 PMC10192653 37216113

[139] Yuan F, et al. CCR5-overexpressing mesenchymal stem cells protect against experimental autoimmune uveitis: insights from single-cell transcriptome analysis. J Neuroinflammation. 2024;21(1):136. 38802924 10.1186/s12974-024-03134-3 PMC11131209

[140] Liu X, et al. Bone marrow mesenchymal stem cells enhance autophagy and help protect cells under hypoxic and retinal detachment conditions. J Cell Mol Med. 2020;24(6):3346–58. 32003125 10.1111/jcmm.15008 PMC7131940

[141] Zwart I, et al. Umbilical cord blood mesenchymal stromal cells are neuroprotective and promote regeneration in a rat optic tract model. Exp Neurol. 2009;216(2):439–48. 19320003 10.1016/j.expneurol.2008.12.028

[142] Levkovitch-Verbin H, et al. Intravitreal injections of neurotrophic factors secreting mesenchymal stem cells are neuroprotective in rat eyes following optic nerve transection. Invest Ophthalmol Vis Sci. 2010;51(12):6394–400. 20926814 10.1167/iovs.09-4310

[143] Zhao T, et al. Protective effects of human umbilical cord blood stem cell intravitreal transplantation against optic nerve injury in rats. Graefes Arch Clin Exp Ophthalmol. 2011;249:1021–8. 21360302 10.1007/s00417-011-1635-7

[144] Zaverucha-do-Valle C, et al. Bone marrow mononuclear cells increase retinal ganglion cell survival and axon regeneration in the adult rat. Cell Transplant. 2011;20(3):391–406. 20719093 10.3727/096368910X524764

[145] Park H-YL, et al. Stem cell-based delivery of brain-derived neurotrophic factor gene in the rat retina. Brain Res. 2012;1469:10–23. 22750585 10.1016/j.brainres.2012.06.006

[146] Mesentier-Louro LA, et al. Cell therapy modulates expression of Tax1-binding protein 1 and synaptotagmin IV in a model of optic nerve lesion. Invest Ophthalmol Vis Sci. 2012;53(8):4720–9. 22695963 10.1167/iovs.11-8198

[147] Chen M, Xiang Z, Cai J. The anti-apoptotic and neuro-protective effects of human umbilical cord blood mesenchymal stem cells (hUCB-MSCs) on acute optic nerve injury is transient. Brain Res. 2013;1532:63–75. 23933426 10.1016/j.brainres.2013.07.037

[148] Jiang B, et al. Intravitreal transplantation of human umbilical cord blood stem cells protects rats from traumatic optic neuropathy. PLoS ONE. 2013;8(8):e69938. 10.1371/journal.pone.0069938 PMC3734232 23940534

[149] Mead B, et al. Intravitreally transplanted dental pulp stem cells promote neuroprotection and axon regeneration of retinal ganglion cells after optic nerve injury. Invest Ophthalmol Vis Sci. 2013;54(12):7544–56. 10.1167/iovs.13-13045 24150755

[150] Mesentier-Louro LA, et al. Distribution of mesenchymal stem cells and effects on neuronal survival and axon regeneration after optic nerve crush and cell therapy. PLoS ONE. 2014;9(10):e110722. 10.1371/journal.pone.0110722 PMC4210195 25347773

[151] Zaverucha-do-Valle C, et al. Sustained effect of bone marrow mononuclear cell therapy in axonal regeneration in a model of optic nerve crush. Brain Res. 2014;1587:54–68. 25204691 10.1016/j.brainres.2014.08.070

[152] Tan H, et al. The therapeutic effects of bone marrow mesenchymal stem cells after optic nerve damage in the adult rat. Clin Interv Aging. 2015:487–90. 10.2147/CIA.S75319 PMC4337419 25733825

[153] Cen L-P, et al. Human periodontal ligament-derived stem cells promote retinal ganglion cell survival and axon regeneration after optic nerve injury. Stem Cells. 2018;36(6):844–55. 29476565 10.1002/stem.2812

[154] Feng X, et al. Transplanted embryonic retinal stem cells have the potential to repair the injured retina in mice. BMC Ophthalmol. 2021;21:1–11. 33422026 10.1186/s12886-020-01795-1 PMC7797095

[155] Becker S, et al. Allogeneic transplantation of Müller-derived retinal ganglion cells improves retinal function in a feline model of ganglion cell depletion. Stem Cells Transl Med. 2016;5(2):192–205. 26718648 10.5966/sctm.2015-0125 PMC4729554

[156] Stanzel BV, et al. Human RPE stem cells grown into polarized RPE monolayers on a polyester matrix are maintained after grafting into rabbit subretinal space. Stem Cell Reports. 2014;2(1):64–77. 10.1016/j.stemcr.2013.11.005 PMC3916756 24511471

[157] Haruta M, et al. In vitro and in vivo characterization of pigment epithelial cells differentiated from primate embryonic stem cells. Invest Ophthalmol Vis Sci. 2004;45(3):1020–5. 14985325 10.1167/iovs.03-1034

[158] Lund RD, et al. Human embryonic stem cell–derived cells rescue visual function in dystrophic RCS rats. Cloning Stem Cells. 2006;8(3):189–99. 17009895 10.1089/clo.2006.8.189

[159] Lund RD, et al. Cells isolated from umbilical cord tissue rescue photoreceptors and visual functions in a rodent model of retinal disease. Stem Cells. 2007;25(3):602–11. 10.1634/stemcells.2006-0308 17053209

[160] Inoue Y, et al. Subretinal transplantation of bone marrow mesenchymal stem cells delays retinal degeneration in the RCS rat model of retinal degeneration. Exp Eye Res. 2007;85(2):234–41. 17570362 10.1016/j.exer.2007.04.007

[161] Carr A-J, et al. Protective effects of human iPS-derived retinal pigment epithelium cell transplantation in the retinal dystrophic rat. PLoS ONE. 2009;4(12):e8152. 10.1371/journal.pone.0008152 PMC2780911 19997644

[162] Lu B, et al. Long-term safety and function of RPE from human embryonic stem cells in preclinical models of macular degeneration. Stem Cells. 2009;27(9):2126–35. 10.1002/stem.149 19521979

[163] Lu B, et al. Human adult bone marrow-derived somatic cells rescue vision in a rodent model of retinal degeneration. Exp Eye Res. 2010;91(3):449–55. 20603115 10.1016/j.exer.2010.06.024

[164] Zhang Y, Wang W. Effects of bone marrow mesenchymal stem cell transplantation on light-damaged retina. Invest Ophthalmol Vis Sci. 2010;51(7):3742–8. 20207980 10.1167/iovs.08-3314

[165] Chung JK, et al. Modulation of retinal wound healing by systemically administered bone marrow-derived mesenchymal stem cells. Korean J Ophthalmol. 2011;25(4):268–74. 21860575 10.3341/kjo.2011.25.4.268 PMC3149139

[166] Kanemura H, et al. Tumorigenicity studies of induced pluripotent stem cell (iPSC)-derived retinal pigment epithelium (RPE) for the treatment of age-related macular degeneration. PLoS ONE. 2014;9(1):e85336. 10.1371/journal.pone.0085336 PMC3891869 24454843

[167] Tzameret A, et al. Transplantation of human bone marrow mesenchymal stem cells as a thin subretinal layer ameliorates retinal degeneration in a rat model of retinal dystrophy. Exp Eye Res. 2014;118:135–44. 24239509 10.1016/j.exer.2013.10.023

[168] Leow S, et al. Safety and efficacy of human Wharton’s jelly-derived mesenchymal stem cells therapy for retinal degeneration. PLoS ONE. 2015;10(6):e0128973. 10.1371/journal.pone.0128973 PMC4479609 26107378

[169] Rotenstreich Y, et al. A novel platform for minimally invasive delivery of cellular therapy as a thin layer across the subretina for treatment of retinal degeneration. Ophthal Technol XXV. 2015. SPIE.

[170] Tzameret A, et al. Epiretinal transplantation of human bone marrow mesenchymal stem cells rescues retinal and vision function in a rat model of retinal degeneration. Stem Cell Res. 2015;15(2):387–94. 10.1016/j.scr.2015.08.007 26322852

[171] Davis RJ, et al. The developmental stage of adult human stem cell-derived retinal pigment epithelium cells influences transplant efficacy for vision rescue. Stem Cell Rep. 2017;9(1):42–9. 10.1016/j.stemcr.2017.05.016 PMC5511099 28625537

[172] Domouky AM, Samy WM, Rashad WA. Therapeutic effect of the mesenchymal stem cells on vigabatrin-induced retinopathy in adult male albino rat. Anat Cell Biol. 2022;55(2):217–28. 10.5115/acb.22.006 PMC9256488 35773221

[173] Liu Q, et al. Comparison of retinal degeneration treatment with four types of different mesenchymal stem cells, human induced pluripotent stem cells and RPE cells in a rat retinal degeneration model. J Transl Med. 2023;21(1):910. 38098048 10.1186/s12967-023-04785-1 PMC10720187

[174] Otani A, et al. Rescue of retinal degeneration by intravitreally injected adult bone marrow–derived lineage-negative hematopoietic stem cells. J Clin Investig. 2004;114(6):765–74. 15372100 10.1172/JCI21686 PMC516263

[175] Aoki H, et al. Transplantation of cells from eye-like structures differentiated from embryonic stem cells in vitro and in vivo regeneration of retinal ganglion-like cells. Graefes Arch Clin Exp Ophthalmol. 2008;246:255–65. 18004585 10.1007/s00417-007-0710-6

[176] Hou H-Y, et al. A therapeutic strategy for choroidal neovascularization based on recruitment of mesenchymal stem cells to the sites of lesions. Mol Ther. 2010;18(10):1837–45. 20647999 10.1038/mt.2010.144 PMC2951561

[177] Tucker BA, et al. Transplantation of adult mouse iPS cell-derived photoreceptor precursors restores retinal structure and function in degenerative mice. PLoS ONE. 2011;6(4):e18992. 10.1371/journal.pone.0018992 PMC3084746 21559507

[178] Hambright D, et al. Long-term survival and differentiation of retinal neurons derived from human embryonic stem cell lines in un-immunosuppressed mouse retina. Mol Vis. 2012;18:920. 22539871 PMC3335781

[179] Gonzalez-Cordero A, et al. Photoreceptor precursors derived from three-dimensional embryonic stem cell cultures integrate and mature within adult degenerate retina. Nat Biotechnol. 2013;31(8):741–7. 23873086 10.1038/nbt.2643 PMC3826328

[180] Assawachananont J, et al. Transplantation of embryonic and induced pluripotent stem cell-derived 3D retinal sheets into retinal degenerative mice. Stem Cell Rep. 2014;2(5):662–74. 10.1016/j.stemcr.2014.03.011 PMC4050483 24936453

[181] Tassoni A, et al. Molecular mechanisms mediating retinal reactive gliosis following bone narrow nesenchymal stem cell transplantation. Stem Cells. 2015;33(10):3006–16. 10.1002/stem.2095 PMC4832383 26175331

[182] Sun J, et al. Protective effects of human iPS-derived retinal pigmented epithelial cells in comparison with human mesenchymal stromal cells and human neural stem cells on the degenerating retina in rd1 mice. Stem Cells. 2015;33(5):1543–53. 25728228 10.1002/stem.1960

[183] Cao J, et al. Human umbilical tissue-derived cells rescue retinal pigment epithelium dysfunction in retinal degeneration. Stem Cells. 2016;34(2):367–79. 26523756 10.1002/stem.2239

[184] Li Z, et al. Neural stem cells transplanted to the subretinal space of rd1 mice delay retinal degeneration by suppressing microglia activation. Cytotherapy. 2016;18(6):771–84. 27067610 10.1016/j.jcyt.2016.03.001

[185] Moisseiev E, et al. Intravitreal administration of human bone marrow CD34+ stem cells in a murine model of retinal degeneration. Invest Ophthalmol Vis Sci. 2016;57(10):4125–35. 27537262 10.1167/iovs.16-19252 PMC6733500

[186] Kruczek K, et al. Differentiation and transplantation of embryonic stem cell-derived cone photoreceptors into a mouse model of end-stage retinal degeneration. Stem Cell Rep. 2017;8(6):1659–74. 28552606 10.1016/j.stemcr.2017.04.030 PMC5470175

[187] Mandai M, et al. iPSC-derived retina transplants improve vision in rd1 end-stage retinal-degeneration mice. Stem Cell Rep. 2017;8(1):69–83. 28076757 10.1016/j.stemcr.2016.12.008 PMC5233464

[188] Ribeiro J, et al. Restoration of visual function in advanced disease after transplantation of purified human pluripotent stem cell-derived cone photoreceptors. Cell Rep. 2021;35(3). 10.1016/j.celrep.2021.109022 PMC8065177 33882303

[189] Zerti D, et al. Transplanted pluripotent stem cell-derived photoreceptor precursors elicit conventional and unusual light responses in mice with advanced retinal degeneration. Stem Cells. 2021;39(7):882–96. 33657251 10.1002/stem.3365

[190] Cholkar K, et al. Eye: Anatomy, physiology and barriers to drug delivery. In: Ocular transporters and receptors. Elsevier; 2013. p. 1–36.

[191] Hassan TA, et al. Reconstruction of rabbit corneal epithelium using adipose and/or bone marrow stem cells. Exp Eye Res. 2025;251: 110203. 39667486 10.1016/j.exer.2024.110203

[192] Gipson, I.K. and M.A. Stepp, Anatomy and cell biology of the cornea, superficial limbus, and conjunctiva. Albert and Jakobiec's Principles and Practice of Ophthalmology, 2022: p. 3–30.

[193] Hertsenberg, A.J. and J.L. Funderburgh. Stem cells in the cornea. Prog Mol Biol Transl Sci. 2015;134:25–41. 26310147 10.1016/bs.pmbts.2015.04.002 PMC5327505

[194] Chastain JE. General considerations in ocular drug delivery. In: Ophthalmic drug delivery systems. CRC Press; 2003. p. 80–129.

[195] Danysh BP, Duncan MK. The lens capsule. Exp Eye Res. 2009;88(2):151–64. 18773892 10.1016/j.exer.2008.08.002 PMC2674021

[196] Norman RE, et al. Dimensions of the human sclera: thickness measurement and regional changes with axial length. Exp Eye Res. 2010;90(2):277–84. 19900442 10.1016/j.exer.2009.11.001

[197] Gupta MP, et al. Retinal anatomy and pathology Retinal Pharmacotherapeutics. 2016;55:7–17. 10.1159/000431128 26502225

[198] Thumann G, Hoffmann S, Hinton DR. Cell biology of the retinal pigment epithelium. In: Retina. Elsevier; 2006. p. 137–52.

[199] Strauss, O., The retinal pigment epithelium in visual function. Physiological reviews, 2005. 10.1152/physrev.00021.2004 15987797

[200] Kaufman, P.L. and A. Alm, Adler's physiology of the eye. 2003: Mosby Inc.

[201] Hildebrand, G.D. and A.R. Fielder, Anatomy and physiology of the retina. Pediatric retina, 2011: p. 39–65.

[202] Zhu M, et al. The human hyaloid system: cell death and vascular regression. Exp Eye Res. 2000;70(6):767–76. 10843781 10.1006/exer.2000.0844

[203] Wurtz, R., et al., Principles of neural science. Kande ER, Schwartz JH, Jessell TM. Central Visual Pathways. 4th Ed. New York (NY): McGraw-Hill, 2000: p. 523–547.

[204] Provis JM. Development of the primate retinal vasculature. Prog Retin Eye Res. 2001;20(6):799–821. 11587918 10.1016/s1350-9462(01)00012-x

[205] GA, C., Ocular circulation. Adler's Physiology of the Eye, 2003: p. 747–784.

[206] Alió del Barrio, J.L., et al., Corneal regeneration using adipose-derived mesenchymal stem cells. Cells, 2022. 11(16): p. 2549. 10.3390/cells11162549 PMC9406486 36010626

[207] Nurković, J.S., R. Vojinović, and Z. Dolićanin, Corneal stem cells as a source of regenerative cell-based therapy. Stem cells international, 2020. 2020. 10.1155/2020/8813447 PMC7388005 32765614

[208] Kasetsuwan, N., et al., Recurrent rates and risk factors associated with recurrent painful bullous keratopathy after primary phototherapeutic keratectomy. Clinical Ophthalmology, 2015: p. 1815–1819. 10.2147/OPTH.S89163 PMC4599175 26491241

[209] Singh M, et al. Role of corneal collagen cross-linking in bullous keratopathy: a systematic review. Indian J Ophthalmol. 2023;71(5):1706–17. 37203022 10.4103/ijo.IJO_1942_22 PMC10391445

[210] GELATT, K.N., Diseases and surgery of the canine cornea and sclera. Animal Eye Research, 2002. 21(3–4): p. 105–113.

[211] Pot SA, et al. Treatment of bullous keratopathy with corneal collagen cross-linking in two dogs. Veterinary ophthalmology. 2015;18(2):168–73. 24373539 10.1111/vop.12137

[212] Jones MK, et al. Cell-based therapeutic strategies for replacement and preservation in retinal degenerative diseases. Prog Retin Eye Res. 2017;58:1–27. 28111323 10.1016/j.preteyeres.2017.01.004 PMC5441967

[213] Holan V, Palacka K, Hermankova B. Mesenchymal stem cell-based therapy for retinal degenerative diseases: experimental models and clinical trials. Cells. 2021;10(3):588. 33799995 10.3390/cells10030588 PMC8001847

[214] Mitchell P, et al. Age-related macular degeneration. The Lancet. 2018;392(10153):1147–59. 10.1016/S0140-6736(18)31550-2 30303083

[215] McMenamin PG, Saban DR, Dando SJ. Immune cells in the retina and choroid: two different tissue environments that require different defenses and surveillance. Prog Retin Eye Res. 2019;70:85–98. 30552975 10.1016/j.preteyeres.2018.12.002 PMC7321801

[216] Leung DY, Tham CC. Normal-tension glaucoma: current concepts and approaches—a review. Clin Experiment Ophthalmol. 2022;50(2):247–59. 35040248 10.1111/ceo.14043

[217] Schnichels S, et al. Retina in a dish: cell cultures, retinal explants and animal models for common diseases of the retina. Prog Retin Eye Res. 2021;81: 100880. 32721458 10.1016/j.preteyeres.2020.100880

[218] Wang W, Lo AC. Diabetic retinopathy: pathophysiology and treatments. Int J Mol Sci. 2018;19(6):1816. 29925789 10.3390/ijms19061816 PMC6032159

[219] Kaur G, Singh NK. Inflammation and retinal degenerative diseases. Neural Regen Res. 2023;18(3):513–8. 36018156 10.4103/1673-5374.350192 PMC9727454

[220] Zarbin MA. Current concepts in the pathogenesis of age-related macular degeneration. Arch Ophthalmol. 2004;122(4):598–614. 15078679 10.1001/archopht.122.4.598

[221] Chen Y-Q, et al. The effects and underlying mechanisms of S-allyl l-cysteine treatment of the retina after ischemia/reperfusion. J Ocul Pharmacol Ther. 2012;28(2):110–7. 22054242 10.1089/jop.2011.0099

[222] Chao H-M, et al. Ferulic acid, but not tetramethylpyrazine, significantly attenuates retinal ischemia/reperfusion-induced alterations by acting as a hydroxyl radical scavenger. J Ocul Pharmacol Ther. 2008;24(5):461–72. 18788996 10.1089/jop.2008.0005

[223] Roh MI, et al. Edaravone, an ROS scavenger, ameliorates photoreceptor cell death after experimental retinal detachment. Invest Ophthalmol Vis Sci. 2011;52(6):3825–31. 21310909 10.1167/iovs.10-6797 PMC3109058

[224] Woo TT, et al. Neuroprotective effects of lutein in a rat model of retinal detachment. Graefes Arch Clin Exp Ophthalmol. 2013;251:41–51. 22899456 10.1007/s00417-012-2128-z PMC3536954

[225] Huang W, et al. Protective effects of resveratrol in experimental retinal detachment. PLoS ONE. 2013;8(9): e75735. 24040416 10.1371/journal.pone.0075735 PMC3770540

[226] Murakami Y, et al. Photoreceptor cell death and rescue in retinal detachment and degenerations. Prog Retin Eye Res. 2013;37:114–40. 23994436 10.1016/j.preteyeres.2013.08.001 PMC3871865

[227] Sève, P., et al., Uveitis: diagnostic work-up. Recommendations from an expert committee. La Revue de Medecine Interne, 2017. 39(9): p. 676–686. 10.1016/j.revmed.2017.09.015 29122311

[228] PLAVONIL, S., et al., Hydroxychloroquine in the treatment of sarcoidosis-associated uveitis and idiopathic uveitis. 2024. 10.1186/s12348-026-00598-7 PMC13407791 42207370

[229] Prete M, et al. Autoimmune uveitis: clinical, pathogenetic, and therapeutic features. Clin Exp Med. 2016;16:125–36. 25820692 10.1007/s10238-015-0345-6

[230] Louveau A, et al. CNS lymphatic drainage and neuroinflammation are regulated by meningeal lymphatic vasculature. Nat Neurosci. 2018;21(10):1380–91. 30224810 10.1038/s41593-018-0227-9 PMC6214619

[231] Chen MS, et al. Nogo-A is a myelin-associated neurite outgrowth inhibitor and an antigen for monoclonal antibody IN-1. Nature. 2000;403(6768):434–9. 10667796 10.1038/35000219

[232] Wang KC, et al. Oligodendrocyte-myelin glycoprotein is a Nogo receptor ligand that inhibits neurite outgrowth. Nature. 2002;417(6892):941–4. 12068310 10.1038/nature00867

[233] Siqueira RC. Stem cell therapy for retinal diseases: update. Stem Cell Res Ther. 2011;2:1–10. 22206617 10.1186/scrt91 PMC3340559

[234] Zarbin M. Cell-based therapy for degenerative retinal disease. Trends Mol Med. 2016;22(2):115–34. 26791247 10.1016/j.molmed.2015.12.007

[235] Shintani K, Shechtman DL, Gurwood AS. Review and update: current treatment trends for patients with retinitis pigmentosa. Optometry-Journal of the American Optometric Association. 2009;80(7):384–401. 19545852 10.1016/j.optm.2008.01.026

[236] Okamoto T, et al. Clonal heterogeneity in differentiation potential of immortalized human mesenchymal stem cells. Biochem Biophys Res Commun. 2002;295(2):354–61. 12150956 10.1016/s0006-291x(02)00661-7

[237] Russell KC, et al. Clonal analysis of the proliferation potential of human bone marrow mesenchymal stem cells as a function of potency. Biotechnol Bioeng. 2011;108(11):2716–26. 21538337 10.1002/bit.23193 PMC3178709

[238] Bennicelli JL, Bennett J. Stem cells set their sights on retinitis pigmentosa. Elife. 2013;2: e01291. 23991287 10.7554/eLife.01291 PMC3755338

[239] Kern S, et al. Comparative analysis of mesenchymal stem cells from bone marrow, umbilical cord blood, or adipose tissue. Stem cells. 2006;24(5):1294–301. 16410387 10.1634/stemcells.2005-0342

[240] Vasiliadis, A.V. and N. Galanis, Human bone marrow-derived mesenchymal stem cells from different bone sources: a panorama. Stem cell investigation, 2020. 7. 10.21037/sci-2020-013 PMC7492710 32964008

[241] Strioga M, et al. Same or not the same? Comparison of adipose tissue-derived versus bone marrow-derived mesenchymal stem and stromal cells. Stem Cells and Development. 2012;21(14):2724–52. 22468918 10.1089/scd.2011.0722

[242] Naji A, et al. Biological functions of mesenchymal stem cells and clinical implications. Cell Mol Life Sci. 2019;76:3323–48. 31055643 10.1007/s00018-019-03125-1 PMC11105258

[243] Eom YW, et al. Rapid isolation of adipose tissue-derived stem cells by the storage of lipoaspirates. Yonsei Med J. 2011;52(6):999–1007. 22028166 10.3349/ymj.2011.52.6.999 PMC3220256

[244] Meyer JS, et al. Embryonic stem cell-derived neural progenitors incorporate into degenerating retina and enhance survival of host photoreceptors. Stem cells. 2006;24(2):274–83. 16123383 10.1634/stemcells.2005-0059 PMC3381839

[245] Lin T-C, et al. Retinal stem cells and potential cell transplantation treatments. J Chin Med Assoc. 2014;77(11):556–61. 25238708 10.1016/j.jcma.2014.08.001

[246] Ripolles-Garcia A, et al. Systemic immunosuppression promotes survival and integration of subretinally implanted human ESC-derived photoreceptor precursors in dogs. Stem Cell Reports. 2022;17(8):1824–41. 35905738 10.1016/j.stemcr.2022.06.009 PMC9391525

[247] Raj SM, et al. Post-operative capsular opacification: a review. International journal of biomedical science: IJBS. 2007;3(4):237. 23675049 PMC3614664

[248] Takahashi, K. and S. Yamanaka, Induction of pluripotent stem cells from mouse embryonic and adult fibroblast cultures by defined factors. cell, 2006. 126(4): p. 663–676. 10.1016/j.cell.2006.07.024 16904174

[249] Wernig M, et al. In vitro reprogramming of fibroblasts into a pluripotent ES-cell-like state. Nature. 2007;448(7151):318–24. 17554336 10.1038/nature05944

[250] Gang J, et al, In vitro mesengenic potential of human umbilical cord blood-derived mesenchymal stem cells. Biochemical and biophysical research communications. 2004;321(1): p. 102-108. 10.1016/j.bbrc.2004.06.111 15358221

[251] Arutyunyan I, et al, Umbilical cord as prospective source for mesenchymal stem cell‐based therapy. Stem cells international, 2016;2016(1): p. 6901286. 10.1155/2016/6901286 PMC5019943 27651799

[252] Parolini, O., et al., Concise review: isolation and characterization of cells from human term placenta: outcome of the first international Workshop on Placenta Derived Stem Cells. Stem cells, 2008;26(2): p. 300-311. 10.1634/stemcells.2007-0594 17975221

[253] Liu I-H, et al. Comparison of the proliferation and differentiation ability between adult rat retinal stem cells and cerebral cortex-derived neural stem cells. Ophthalmologica. 2005;219(3):171–6. 15947503 10.1159/000085250

[254] Gronthos S, et al. Postnatal human dental pulp stem cells (DPSCs) in vitro and in vivo. Proc Natl Acad Sci. 2000;97(25):13625–30. 11087820 10.1073/pnas.240309797 PMC17626

[255] Ye J, et al. Bone marrow-derived progenitor cells promote corneal wound healing following alkali injury. Graefes Arch Clin Exp Ophthalmol. 2008;246:217–22. 18075751 10.1007/s00417-007-0716-0

[256] Kang SK, et al. Journey of mesenchymal stem cells for homing: strategies to enhance efficacy and safety of stem cell therapy. Stem cells international. 2012;2012(1): 342968. 22754575 10.1155/2012/342968 PMC3382267

[257] Lan Y, et al. Kinetics and function of mesenchymal stem cells in corneal injury. Invest Ophthalmol Vis Sci. 2012;53(7):3638–44. 22562508 10.1167/iovs.11-9311

[258] Ghasemi H. Roles of IL-6 in ocular inflammation: a review. Ocul Immunol Inflamm. 2018;26(1):37–50. 28146368 10.1080/09273948.2016.1277247

[259] Theerakittayakorn K, et al. Differentiation induction of human stem cells for corneal epithelial regeneration. Int J Mol Sci. 2020;21(21):7834. 33105778 10.3390/ijms21217834 PMC7660084

[260] Chen J, et al. Small-molecule induction promotes corneal endothelial cell differentiation from human iPS cells. Frontiers in Bioengineering and Biotechnology. 2021;9: 788987. 34976977 10.3389/fbioe.2021.788987 PMC8714889

[261] Setiawan AM, Kamarudin TA. Differentiation of human mesenchymal stem cells into corneal epithelial cells: current progress. Curr Issues Mol Biol. 2024;46(12):13281–95. 39727920 10.3390/cimb46120792 PMC11674674

[262] Konala VBR, et al. The current landscape of the mesenchymal stromal cell secretome: a new paradigm for cell-free regeneration. Cytotherapy. 2016;18(1):13–24. 26631828 10.1016/j.jcyt.2015.10.008 PMC4924535

[263] Salehi H, et al. Overview of retinal differentiation potential of mesenchymal stem cells: a promising approach for retinal cell therapy. Annals of Anatomy-Anatomischer Anzeiger. 2017;210:52–63. 10.1016/j.aanat.2016.11.010 27986614

[264] Noverina R, et al. Growth factors profile in conditioned medium human adipose tissue-derived mesenchymal stem cells (CM-hATMSCs). Clinical Nutrition Experimental. 2019;24:34–44.

[265] Taylor AW. Ocular immune privilege and transplantation. Front Immunol. 2016;7:37. 26904026 10.3389/fimmu.2016.00037 PMC4744940

[266] Keino H, Horie S, Sugita S. Immune privilege and eye-derived T-regulatory cells. J Immunol Res. 2018;2018(1):1679197. 29888291 10.1155/2018/1679197 PMC5985108

[267] de Castro LL, et al. Current understanding of the immunosuppressive properties of mesenchymal stromal cells. J Mol Med. 2019;97:605–18. 30903229 10.1007/s00109-019-01776-y

[268] Wang M, Yuan Q, Xie L. Mesenchymal stem cell-based immunomodulation: properties and clinical application. Stem cells international. 2018;2018(1):3057624. 30013600 10.1155/2018/3057624 PMC6022321

[269] Dostert G, et al. How do mesenchymal stem cells influence or are influenced by microenvironment through extracellular vesicles communication? Frontiers in Cell and Developmental Biology. 2017;5:6. 28224125 10.3389/fcell.2017.00006 PMC5293793

[270] Paulus, Y.M. and A. Sodhi, Anti-angiogenic therapy for retinal disease. Pharmacologic Therapy of Ocular Disease, 2017: p. 271–307. 10.1007/164_2016_78 PMC5451313 27783271

[271] Mansoor H, et al. Current trends and future perspective of mesenchymal stem cells and exosomes in corneal diseases. Int J Mol Sci. 2019;20(12):2853. 31212734 10.3390/ijms20122853 PMC6627168

[272] Trost, A., et al., Pericytes in the retina. Pericyte Biology in Different Organs, 2019: p. 1–26. 10.1007/978-3-030-11093-2_1 30937860

[273] Fiori A, et al. Mesenchymal stromal/stem cells as potential therapy in diabetic retinopathy. Immunobiology. 2018;223(12):729–43. 29402461 10.1016/j.imbio.2018.01.001

[274] da Silva Meirelles L, et al. The gene expression profile of non-cultured, highly purified human adipose tissue pericytes: Transcriptomic evidence that pericytes are stem cells in human adipose tissue. Exp Cell Res. 2016;349(2):239–54. 27789253 10.1016/j.yexcr.2016.10.017

[275] Sinclair KA, et al. Characterization of intercellular communication and mitochondrial donation by mesenchymal stromal cells derived from the human lung. Stem Cell Res Ther. 2016;7:1–10. 27406134 10.1186/s13287-016-0354-8 PMC4942965

[276] Feng Y, et al. Human bone marrow mesenchymal stem cells rescue endothelial cells experiencing chemotherapy stress by mitochondrial transfer via tunneling nanotubes. Stem Cells and Development. 2019;28(10):674–82. 30808254 10.1089/scd.2018.0248

[277] Mead B, Amaral J, Tomarev S. Mesenchymal stem cell–derived small extracellular vesicles promote neuroprotection in rodent models of glaucoma. Invest Ophthalmol Vis Sci. 2018;59(2):702–14. 29392316 10.1167/iovs.17-22855 PMC5795911

[278] Schwartz SD, et al. Embryonic stem cell trials for macular degeneration: a preliminary report. The Lancet. 2012;379(9817):713–20. 10.1016/S0140-6736(12)60028-2 22281388

[279] Soleimani M, et al. Applications of mesenchymal stem cells in ocular surface diseases: sources and routes of delivery. Expert Opin Biol Ther. 2023;23(6):509–25. 36719365 10.1080/14712598.2023.2175605 PMC10313829

[280] Eveleth DD. Cell-based therapies for ocular disease. J Ocul Pharmacol Ther. 2013;29(10):844–54. 24050306 10.1089/jop.2013.0028

[281] Galindo S, et al. Subconjunctival injection of mesenchymal stem cells for corneal failure due to limbal stem cell deficiency: state of the art. Stem Cell Res Ther. 2021;12:1–12. 33441175 10.1186/s13287-020-02129-0 PMC7805216

[282] Galindo S, et al. Therapeutic effect of human adipose tissue-derived mesenchymal stem cells in experimental corneal failure due to limbal stem cell niche damage. Stem Cells. 2017;35(10):2160–74. 28758321 10.1002/stem.2672

[283] Stevens S. Administering a subconjunctival injection. Community Eye Health. 2009;22(69):15. 19506718 PMC2683560

[284] Coulson-Thomas VJ, Caterson B, Kao WW-Y. Transplantation of human umbilical mesenchymal stem cells cures the corneal defects of mucopolysaccharidosis VII mice. Stem Cells. 2013;31(10):2116–26. 23897660 10.1002/stem.1481 PMC3812352

[285] Kuriyan AE, et al. Vision loss after intravitreal injection of autologous “stem cells” for AMD. N Engl J Med. 2017;376(11):1047–53. 28296617 10.1056/NEJMoa1609583 PMC5551890

[286] Peng Y, Tang L, Zhou Y. Subretinal injection: a review on the novel route of therapeutic delivery for vitreoretinal diseases. Ophthalmic Res. 2017;58(4):217–26. 28858866 10.1159/000479157

[287] Mahmood A, et al. Treatment of traumatic brain injury in adult rats with intravenous administration of human bone marrow stromal cells. Neurosurgery. 2003;53(3):697–703. 12943585 10.1227/01.neu.0000079333.61863.aa

[288] Ding SSL, et al. Empowering mesenchymal stem cells for ocular degenerative disorders. Int J Mol Sci. 2019;20(7):1784. 30974904 10.3390/ijms20071784 PMC6480671

[289] Nicoară SD, et al. Novel strategies for the improvement of stem cells’ transplantation in degenerative retinal diseases. Stem cells international. 2016;2016(1):1236721. 27293444 10.1155/2016/1236721 PMC4887645

[290] Guo Y, et al. SDF-1/CXCL12 enhances survival and chemotaxis of murine embryonic stem cells and production of primitive and definitive hematopoietic progenitor cells. Stem cells. 2005;23(9):1324–32. 16210409 10.1634/stemcells.2005-0085

[291] Ogilvie JM, Speck JD, Lett JM. Growth factors in combination, but not individually, rescue rd mouse photoreceptors in organ culture. Exp Neurol. 2000;161(2):676–85. 10686086 10.1006/exnr.1999.7291

[292] Henriksson JT, McDermott AM, Bergmanson JP. Dimensions and morphology of the cornea in three strains of mice. Invest Ophthalmol Vis Sci. 2009;50(8):3648–54. 19264894 10.1167/iovs.08-2941 PMC2752418

[293] Atalay E, et al. Animal models for limbal stem cell deficiency: a critical narrative literature review. Ophthalmol Therapy. 2024;13(3):671–96. 10.1007/s40123-023-00880-0 PMC10853161 38280103

[294] Stepp MA, et al. Wounding the cornea to learn how it heals. Exp Eye Res. 2014;121:178–93. 24607489 10.1016/j.exer.2014.02.007 PMC4072315

[295] Valdez-Garcia JE, Lozano-Ramirez JF, Zavala J. Adult white New Zealand rabbit as suitable model for corneal endothelial engineering. BMC Res Notes. 2015;8:1–4. 25648773 10.1186/s13104-015-0995-1 PMC4322652

[296] Suri R, Beg S, Kohli K. Target strategies for drug delivery bypassing ocular barriers. Journal of drug delivery science and technology. 2020;55: 101389.

[297] Leclercq B, Mejlachowicz D, Behar-Cohen F. Ocular barriers and their influence on gene therapy products delivery. Pharmaceutics. 2022;14(5):998. 35631584 10.3390/pharmaceutics14050998 PMC9143174

[298] Mölzer C, et al. Immune privilege: the microbiome and uveitis. Front Immunol. 2021;11: 608377. 33569055 10.3389/fimmu.2020.608377 PMC7868421

[299] Yañez-Soto B, et al. Interfacial phenomena and the ocular surface. Ocul Surf. 2014;12(3):178–201. 24999101 10.1016/j.jtos.2014.01.004

[300] Ambati J, et al. Diffusion of high molecular weight compounds through sclera. Invest Ophthalmol Vis Sci. 2000;41(5):1181–5. 10752958

[301] Reimondez-Troitiño S, et al. Nanotherapies for the treatment of ocular diseases. Eur J Pharm Biopharm. 2015;95:279–93. 25725262 10.1016/j.ejpb.2015.02.019

[302] Dey S, et al. Molecular evidence and functional expression of P-glycoprotein (MDR1) in human and rabbit cornea and corneal epithelial cell lines. Invest Ophthalmol Vis Sci. 2003;44(7):2909–18. 12824231 10.1167/iovs.02-1142

[303] Dalkara D, et al. Inner limiting membrane barriers to AAV-mediated retinal transduction from the vitreous. Mol Ther. 2009;17(12):2096–102. 19672248 10.1038/mt.2009.181 PMC2814392

[304] HS Boddu, S., H. Gupta, and S. Patel, Drug delivery to the back of the eye following topical administration: an update on research and patenting activity. Recent patents on drug delivery & formulation, 2014. 8(1): p. 27–36. 10.2174/1872211308666140130093301 24475918

[305] Paolicelli P, et al. Chitosan nanoparticles for drug delivery to the eye. Expert Opin Drug Deliv. 2009;6(3):239–53. 19290841 10.1517/17425240902762818

[306] de la Fuente M, et al. Chitosan-based nanostructures: a delivery platform for ocular therapeutics. Adv Drug Deliv Rev. 2010;62(1):100–17. 19958805 10.1016/j.addr.2009.11.026

[307] Souza JG, et al. Topical delivery of ocular therapeutics: carrier systems and physical methods. J Pharm Pharmacol. 2014;66(4):507–30. 24635555 10.1111/jphp.12132

